# Coupled Proliferation and Apoptosis Maintain the Rapid Turnover of Microglia in the Adult Brain

**DOI:** 10.1016/j.celrep.2016.12.041

**Published:** 2017-01-10

**Authors:** Katharine Askew, Kaizhen Li, Adrian Olmos-Alonso, Fernando Garcia-Moreno, Yajie Liang, Philippa Richardson, Tom Tipton, Mark A. Chapman, Kristoffer Riecken, Sol Beccari, Amanda Sierra, Zoltán Molnár, Mark S. Cragg, Olga Garaschuk, V. Hugh Perry, Diego Gomez-Nicola

**Affiliations:** 1 Biological Sciences, University of Southampton, Southampton General Hospital, Southampton SO16 6YD, UK; 2 Institute of Physiology II, University of Tübingen, 72074 Tübingen, Germany; 3 Department of Physiology, Anatomy and Genetics, University of Oxford, Oxford OX1 3PA, UK; 4 Antibody and Vaccine Group, Cancer Sciences Unit, University of Southampton, Southampton SO16 6YD, UK; 5 Research Department Cell and Gene Therapy, University Medical Center Hamburg-Eppendorf, 20246 Hamburg, Germany; 6 Achucarro Basque Center for Neuroscience, Ikerbasque Foundation, University of the Basque Country (UPV/EHU), 48940 Leioa, Bizkaia, Spain

**Keywords:** self-renewal, BrdU, CSF1R, CX3CR1, Macgreen, Vav-Bcl2, RNA-seq

## Abstract

Microglia play key roles in brain development, homeostasis, and function, and it is widely assumed that the adult population is long lived and maintained by self-renewal. However, the precise temporal and spatial dynamics of the microglial population are unknown. We show in mice and humans that the turnover of microglia is remarkably fast, allowing the whole population to be renewed several times during a lifetime. The number of microglial cells remains steady from late postnatal stages until aging and is maintained by the spatial and temporal coupling of proliferation and apoptosis, as shown by pulse-chase studies, chronic in vivo imaging of microglia, and the use of mouse models of dysregulated apoptosis. Our results reveal that the microglial population is constantly and rapidly remodeled, expanding our understanding of its role in the maintenance of brain homeostasis.

## Highlights

• The microglial population is formed without the perinatal infiltration of monocytes
• The microglial density remains remarkably stable over a mouse or human lifetime
• In the mouse and human brain, microglia turn over several times during a lifetime
• Microglia self-renewal is maintained by coupled proliferation and apoptosis

## Graphical Abstract

**Figure:**
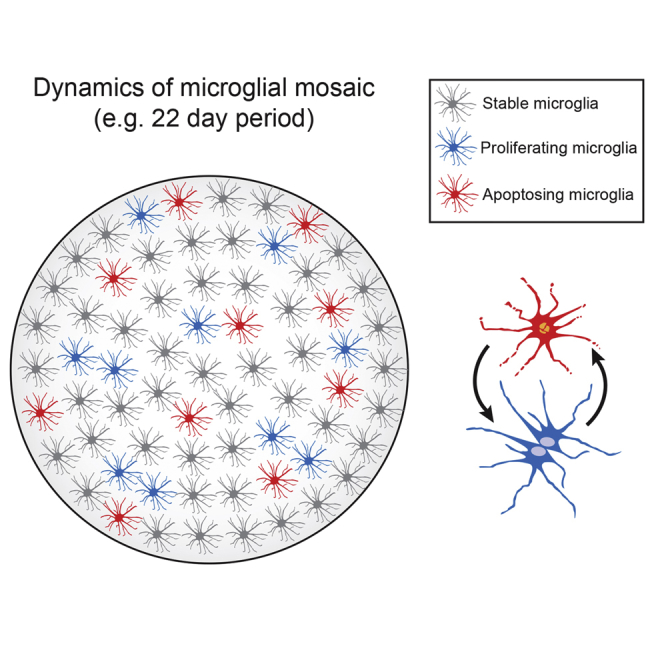

## Teaser

The mechanism or mechanisms underlying microglial homeostasis are unknown. Askew et al. show that microglia self-renewal is maintained by coupled proliferation and apoptosis, resulting in a stable microglia number over a mouse or human lifetime.

## Introduction

Microglial cells are the brain’s resident innate immune cells, with proposed key roles in brain communication and the control of inflammation in brain disease (Gomez-Nicola and Perry, 2015), the developmental control of neurogenesis (Cunningham et al., 2013), wiring (Squarzoni et al., 2014) and synaptic pruning (Paolicelli et al., 2011), the monitoring of synaptic activity (Wake et al., 2009) and the regulation of adult neurogenesis (Sierra et al., 2010). Microglia account for 5%–12% of the total number of glial cells in the mouse brain (Lawson et al., 1990) and 0.5%–16.6% of the total number of cells in the human brain (Mittelbronn et al., 2001), depending on the region studied.

Microglia are derived from the yolk sac at embryonic day (E) 8.5 (Ginhoux et al., 2010), a lineage distinct from most other tissue-resident macrophages (Hoeffel et al., 2015), and they acquire their definitive local density soon after birth, after a wave of microglial proliferation at early postnatal stages (Nikodemova et al., 2015). However, it is unclear whether proliferation alone can account for the rapid increase in microglial numbers and suggests the possibility of additional recruitment and differentiation from blood-derived monocytes perinatally (Ginhoux et al., 2013), although the contribution of monocytes has not been observed in fate mapping studies (Hoeffel et al., 2015, Sheng et al., 2015).

In the adult, it has been suggested that the microglial population is long lived and maintained by self-renewal (Lawson et al., 1992), although the dynamics of the microglial population in the adult brain are largely unknown. However, evidence arising from manipulations of the numbers of resident microglial cells highlights that this population can be rapidly reconstituted by the proliferation of resident cells after genetic ablation by using the Cx3cr1CreER-based system (Bruttger et al., 2015), by pharmacological elimination (Elmore et al., 2014), or by infiltrating monocytes after the death of microglia induced using the CD11b-TK system (Varvel et al., 2012). Microglia are rarely replaced by bone marrow (BM)-derived progenitors in health or disease (Gomez-Nicola and Perry, 2015). These observations suggest that microglia resemble the behavior of other tissue-resident macrophage populations, like lung or BM macrophages, which are maintained by self-renewal in the steady state (Hashimoto et al., 2013). Although these studies suggest that microglia are a dynamic population and give some clues about the molecular determinants of the repopulation response, we do not know the rules governing the homeostatic maintenance of microglia during an organism’s lifetime.

In this study, we show that the adult microglial population is formed without a contribution from circulating progenitors. We show that in the adult mouse and human brain, microglia display a high proliferation rate that accounts for several rounds of renewal of the whole population during the organism’s lifetime. This proliferation is temporally and spatially coupled to intrinsic apoptosis, resulting in the maintenance of a relatively steady number of cells from early postnatal stages through to aging. Our results reveal highly dynamic but tightly regulated control of microglial cell numbers, expanding our understanding of the functions of microglia in the healthy and diseased brain.

## Results

### A Perinatal Wave of Infiltrating Monocytes Does Not Contribute to the Adult Microglial Population

**Figure 1:**
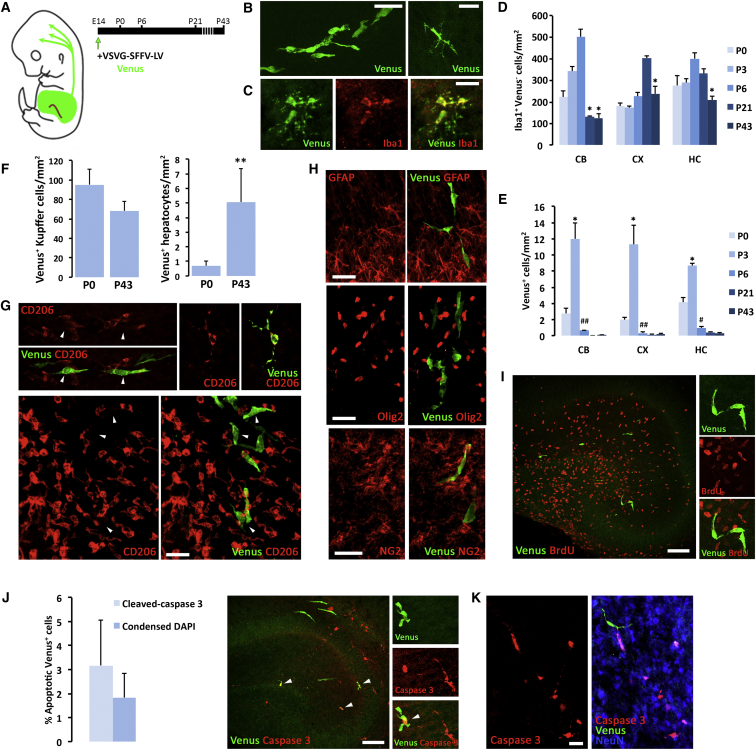
A Wave of Infiltrating Monocytes Invades the Brain at Early Postnatal Stages to Be Rapidly Depleted and Not Contributing to the Adult Microglial Population (A) Experimental design, illustrating the tracing of late embryonic hematopoiesis by the intra-utero marking of liver progenitors with VSVG-SFFV lentiviral vectors (E14) and subsequent analysis of brain infiltration (P0–P43). (B and C) Representative examples of Venus^+^ (green) infiltrating cells at P3 (cerebellum), with migratory (bipolar, elongated) (B) or ramified (multiple radially orientated processes) (B, right) morphologies (C). Iba1 expression is shown in red in differentiated ramified cells. (D and E) Time-course analysis of the number of resident microglia (Iba1^+^Venus^−^) and infiltrating monocytes (Venus^+^) in the postnatal cerebellum (CB), cortex (CX), and hippocampus (HC). At all ages tested, Venus^+^ cells (E) represent only a minority of all Iba1^+^ cells (D). (F–H) Phenotypic characterization of Venus^+^ cells at P3 by confocal microscopy (G and H). Venus^+^ cells (arrowheads) are CD206^low^ (red, F) and GFAP^−^, Olig2^−^, and NG2^−^ (red, H). (I) Representative example of the absence of cell proliferation (BrdU^+^; red) in Venus^−^ cells in the mouse postnatal hippocampus (P3). (J) Quantification of the apoptosis of Venus^+^ cells in the brain (cortex, hippocampus, and cerebellum) at P3, analyzed as expression of cleaved caspase-3 or condensation of chromatin (DAPI). A representative example of the expression of cleaved caspase-3 (red) in Venus^+^ cells (green) is shown. (K) Expression of cleaved caspase-3 in NeuN^+^ neurons at P3. Venus^+^ cells are shown in green. Scale bars are 20 μm in (B), (C), (G), (H), and (K) and 100 μm in (I) and (J). Data shown in (D), (E), (F), and (J) are represented as mean ± SEM (n = 6). Statistical differences: (D) CB ^∗^p < 0.05 versus P6, CX ^∗^p < 0.05 versus P21, HC ^∗^p < 0.05 versus P6. (E) ^∗^p < 0.05 versus P0, #p < 0.05 versus P3, ##p < 0.01 versus P3. (F) ^∗∗^p < 0.01. Data were analyzed with a two-way ANOVA and a post hoc Tukey test (D and E) or a t test (F).

Although evidence supports the concept that the adult microglial population is generated from yolk sac emigrants (Ginhoux et al., 2010), followed by a wave of microglial proliferation (Nikodemova et al., 2015), it is unclear whether this alone accounts for the total increase in microglial cell numbers. Contrasting reports described the infiltration of blood-derived monocytes into the brain at perinatal stages (Alliot et al., 1999, Tambuyzer et al., 2009), although monocytes have been shown not to contribute to the adult microglial population (Hoeffel et al., 2015, Sheng et al., 2015). To address this question, we used an approach to label and track hematopoietic cells during embryonic development that is based on the in utero intra-liver delivery of lentiviral LeGO vectors driving the expression of the fluorescent protein Venus at E14, a stage when the liver is the main hematopoietic organ (Figure 1A). This method allows rapid, selective, and minimally invasive tracing of cells from the hematopoietic lineage and further analysis in target organs. Intra-liver tracing at E14 and subsequent analysis of the brain from postnatal day (P) 0 onward allowed the visualization of waves of infiltrating monocytes (Venus^+^), acquiring migratory phenotypes (bipolar, elongated; 87.9% of all Venus^+^ cells at P3) or ramified phenotypes (multiple radially orientated processes; 12.0% of all Venus^+^ cells at P3) within the brain’s parenchyma (Figures 1B and 1C). The visualization of the differentiation of Venus^+^ cells into ramified Iba1-expressing morphologies supported the use of this tracking method for long-term purposes, because the expression of the Venus transgene was not affected by phenotypic changes with postnatal age (Figure 1C). Venus^+^ cells were not found in the perivascular space of the blood vessels or in the meninges. This, together with the morphologies observed in Figure 1C, supports that Venus^+^ cells infiltrate the parenchyma proper. The quantification of the total Venus^+^ cells (ramified + migratory) (Figures 1B and 1C) showed the time course of infiltration (Figures 1D and 1E). This wave of infiltration was coincident in time with the expansion of the resident microglial population (Iba1^+^Venus^−^) followed by further refinement (Figure 1D), in accordance with previously reported data (Nikodemova et al., 2015). After a peak of infiltration at P3, the infiltrated monocytes were found to radically decrease in number until only rare cells survive in the adult brain (Figure 1E). These cells were not observed when vectors were delivered to the amniotic sac, supporting the specificity of the intra-liver approach. Intra-liver injections were comparable in different litters, as shown by a comparable degree of labeling of Kupffer cells and hepatocytes in the liver (Figure 1F), preserved over time. Venus^+^ cells in the brain parenchyma were identified at P3 to be CD206^low^ when compared with choroid plexus or perivascular macrophages (Figure 1G) and GFAP^−^Olig2^−^NG2^−^ (Figure 1H), supporting their monocytic lineage. Venus^+^ cells were also defined as non-proliferative cells (Venus^+^ bromodeoxyuridine [BrdU]^−^) in the different regions analyzed (Figure 1I).

The analysis of cell death in the Venus^+^ population revealed an apoptotic response from P3, as identified by the expression of cleaved caspase-3 or chromatin condensation (non-significant difference between activated caspase-3^+^ cells versus cells with condensed nuclei stained with DAPI) (Figure 1J). We found that at P3, 1.83% (condensed DAPI) or 3.17% (act-caspase-3^+^) of Venus^+^ cells were apoptotic, with small variations across regions. Other cells, mostly neurons, were also found to be activated caspase-3^+^ (Figure 1K) because of postnatal circuitry refinement. Given that the average time for an apoptotic cell to be removed from the brain is about 80 min (Sierra et al., 2013), and assuming that the clearance rates remain constant until adulthood, we estimated that the infiltrated monocytes could be removed from the brain parenchyma within approximately 42–72 hr. Although this is an estimation based on the mean rate of observed apoptosis, it helps explain the drop in Venus^+^ cell numbers detected from P3 to P6 (Figure 1E).

In light of these data, we conclude that the adult microglial population is composed exclusively from yolk sac-derived cells, without the contribution of hematopoietic-derived monocytes infiltrating at perinatal stages.

### The Number of Microglial Cells Remains Stable throughout Life in Mice and Humans

**Figure 2:**
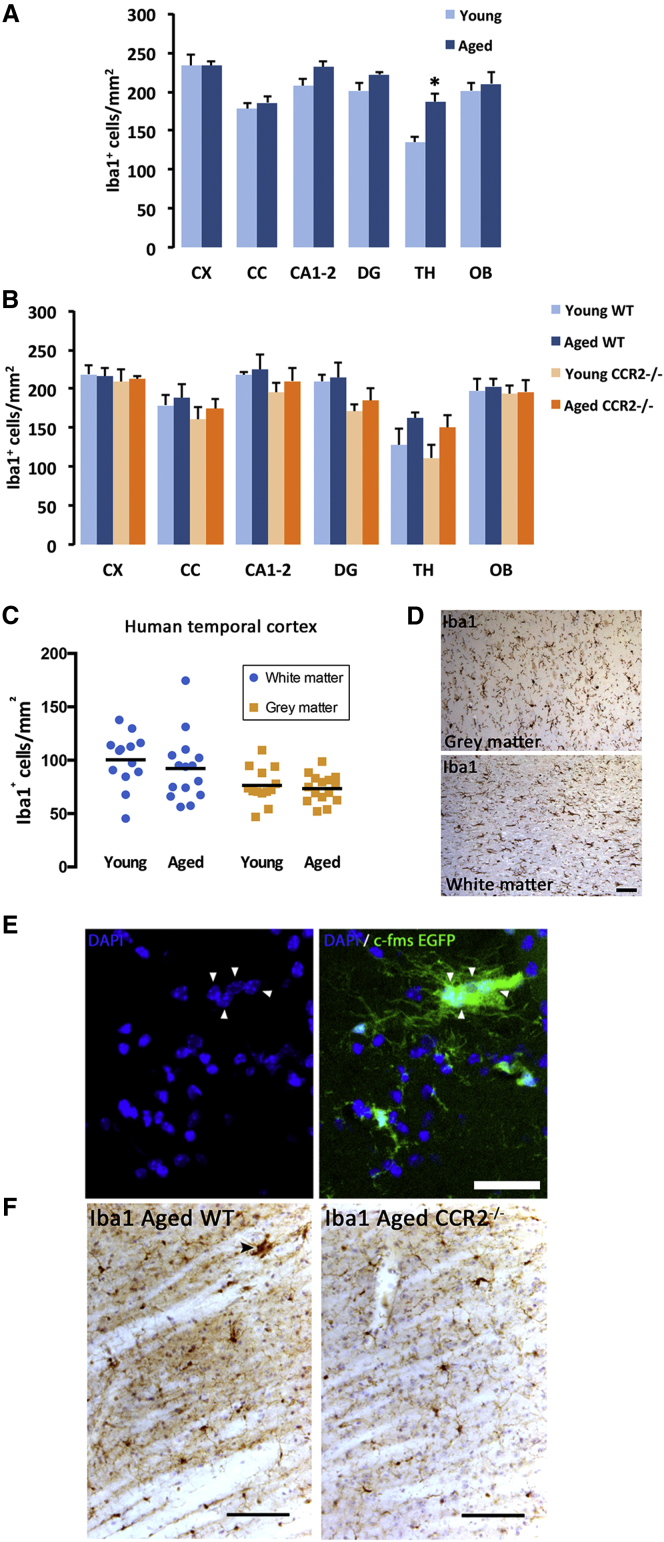
The Density of Microglial Cells Remains Steady Through the Lifetime, without a Significant Contribution of Circulating Monocytes (A) Quantification of microglial density (Iba1^+^ cells) across brain regions (CX, cortex; CC, corpus callosum; CA1–2, hippocampal CA1–CA2; DG, dentate gyrus; TH, thalamus; OB, olfactory bulb) in young (4–6 months) and aged (18–24 months) mice. (B) Quantification of microglial density (Iba1^+^ cells) across brain regions (A) in young (4–6 months) and aged (18–24 months) wild-type (WT) or CCR2^−/−^ mice. (C) Quantification of microglial density (Iba1^+^ cells) in the white and gray matter of the human temporal cortex in young or aged individuals. (D) Representative images of Iba1 staining in human temporal cortex. (E) Representative example of a multinucleated microglial aggregate (c-fms EGFP) in aging mice. (F) Representative examples of multinucleated microglial aggregates in aging WT mice, absent from CCR2^−/−^ mice. Scale bars are 50 μm in (D) and (E) and 50 μm in (F). Data shown are represented as mean ± SEM. n = 7 (A and B), n = 15 (C). Statistical differences: ^∗^p < 0.05. Data were analyzed with a two-way ANOVA and a post hoc Tukey test (A–C).

We next investigated the regional and temporal changes in the number of microglial cells in a select number of brain regions to understand their population dynamics. The density of murine microglial cells (Iba1^+^) remained remarkably stable throughout, with little change from the young (4–6 months) to the aged (18–24 months) brains in all areas analyzed except the thalamus, where an increased number was found with aging (Figure 2A). Microglial cells were denser in gray matter-enriched versus white matter-enriched areas, as previously shown (Lawson et al., 1990). To better understand whether the maintenance of microglial numbers was achieved by local self-renewal or by a contribution from circulating monocytes, we compared the microglial density in young versus aged CCR2^−/−^ and wild-type (WT) mice. CCR2^−/−^ monocytes have deficient egress from the bone marrow, leading to fewer circulating monocytes (Serbina and Pamer, 2006) and making them a valuable model in which to study the role of recruited monocytes (Gomez-Nicola and Perry, 2015). The contribution of patrolling monocytes (CX3CR1^+^/CCR2^−^) was not studied by our approach and cannot be excluded, although these cells have been shown to infiltrate the CNS only under pathological conditions (Shechter et al., 2013). Neither young nor aged CCR2^−/−^ mice had a different number of microglial cells when compared to WT mice (Figure 2B), suggesting that circulating monocytes do not contribute significantly to the microglial population during a healthy lifetime.

A similar picture was observed when we compared young (20–35 years old) versus aged (58–76 years old) human cases. The density of microglia in the gray or white matter of the temporal cortex was found to be unchanged with aging (Figure 2C). Microglial cell density was greater in the white matter than in the gray matter (Figures 2C and 2D), in agreement with previous findings (Mittelbronn et al., 2001). This pattern of distribution opposes that previously found in rodents (Lawson et al., 1990), indicating species-specific regional differences in the microglial population.

Altogether, these data demonstrate that the density of microglial cells is remarkably stable in young and aged brains from both mice and humans.

The analysis of microglia in aged mice led to the identification of small numbers of multinucleated microglial aggregates (Figure 2E), previously described in the aged rat brain (Perry et al., 1993). Multinucleated microglial aggregates were more frequent in the aged thalamus and cerebellum (Figure S1A) and expressed major histocompatibility complex class II (MHC class II) (Figure S1B), as well as CD45 (data not shown). Using confocal microscopy, we could identify aggregates containing up to ten nuclei within the same cytoplasmic syncytium (Figure S1C). To better understand whether failed cytokinesis after increased proliferation was the origin of these aggregates, we performed analysis of proliferation after repeated BrdU incorporation using confocal microscopy (Figure S1D). The incorporation of BrdU was minimal in these aggregates (0.48% were BrdU^+^ and showed only two cells per aggregate) (Figure S1D), ruling out the hypothesis of failed cytokinesis. Our next hypothesis was that these aggregates could have a peripheral origin, which was confirmed after observing that aged CCR2^−/−^ mice were devoid of multinucleated microglial aggregates (Figure 2F).

### Microglia Have a High Proliferation Rate in the Mouse and Human Brain

**Figure 3:**
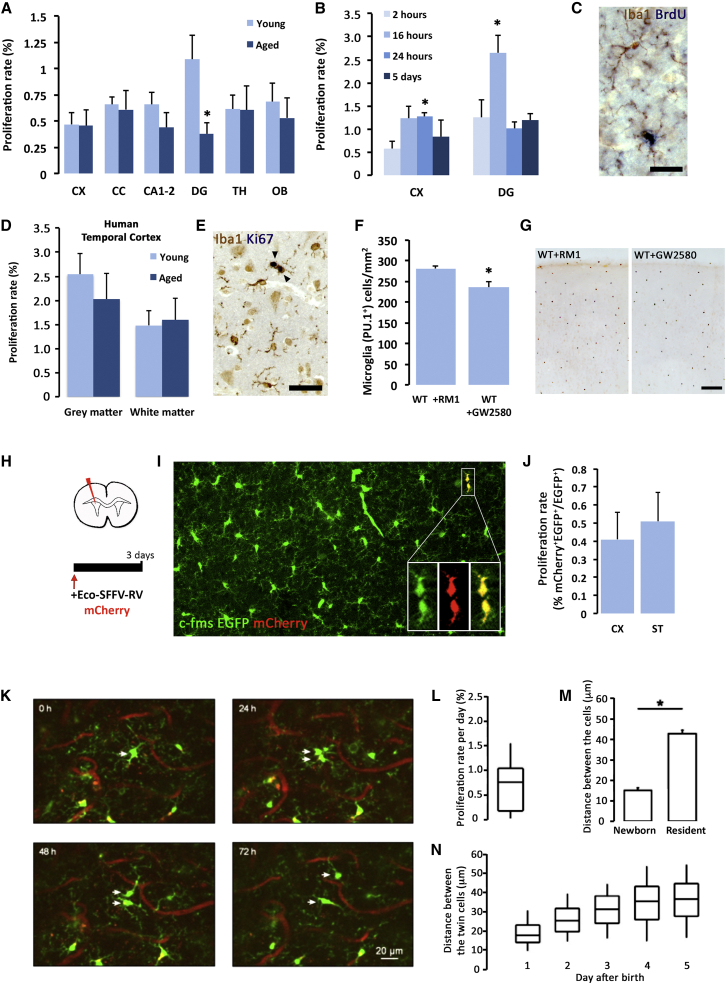
Proliferation of Microglia in the Adult Mouse and Human Brain (A) Analysis of the proliferation (proliferation rate, %) of microglia across brain regions (CX, cortex; CC, corpus callosum; CA1–2, hippocampal CA1–CA2; DG, dentate gyrus; TH, thalamus; OB, olfactory bulb) in young (4–6 months) and aged (18–24 months) mice. (B) Time-course analysis of microglial proliferation (proliferation rate, %) and death in the mouse cortex (CX) and dentate gyrus (DG). (C) Representative example of a proliferating microglial cell (Iba1^+^, brown), incorporating BrdU (blue). (D and E) Analysis of the proliferation (proliferation rate, %) of microglia in the human white or gray matter of the temporal cortex, analyzed as expression of Ki67 (blue) in Iba1^+^ cells (brown), as shown in the representative example (E). (H–J) Analysis of microglial proliferation by tracing c-fms EGFP mice with Eco-SFFV mCherry γ-retroviral vectors (Eco-SFFV-RV mCherry). (H) Experimental scheme. (I) Representative image of the tracing of proliferating microglia by Eco-SFFV-RV (mCherry, red) in the cortex of c-fms EGFP mice (green). (J) Analysis of the proliferation (proliferation rate, % mCherry^+^EGFP^+^/total EGFP^+^) of microglia (CX, cortex; ST, striatum) in c-fms EGFP mice.(K–N) Analysis of microglial proliferation by two-photon imaging of CX_3_CR1^GFP/+^ mice. (K) Maximal intensity projection (MIP) images of the same field of view (142–153 μm depth, 1 μm step) in a CX_3_CR1^GFP/+^ mouse taken at different time points as indicated (see timestamps, relative time). Arrows point to a proliferating microglial cell and its progeny. (L) Proliferation rate of microglia (median ± interquartile range [IQR]; n = 669 cells, 9 fields of view [FOVs], and 4 mice). (M) Mean distance between the centers of two neighboring cells for resident cells and for newborn cells during the first 24 hr of their life (mean ± SEM; n = 62 cells, 9 FOVs, and 4 mice). (N) Distance between the twin microglial cells as a function of their age (median ± IQR; n = 31 pairs of twin cells, 8 FOVs, and 4 mice). Scale bars are 20 μm in (A) and (C), 50 μm in (E), and 100 μm in (G). Data shown are represented as mean ± SEM. n = 8 (A and B), n = 15 (D), n = 6 (F), n = 5 (J). Statistical differences: (A–J) ^∗^p < 0.05; (M) ^∗^p < 0.001, Student’s t test. Data were analyzed with a two-way ANOVA and a post hoc Tukey test (A and B) or a Student’s t test (F and J).

Although it is often assumed that the microglial population is maintained by a slow turnover of long-lived resident cells, little formal evidence exists (reviewed in Gomez-Nicola and Perry, 2015). Earlier work from Lawson et al. (1992), using H^3^ thymidine combined with immunohistochemistry for F4/80, demonstrated that microglia proliferate in the healthy brain but do so more slowly than other tissue macrophages: 0.05% of the microglia were proliferating at a given time, 20 times less than the lowest labeling index for any other resident macrophage populations studied (Lawson et al., 1992). We set out to analyze the proliferation of resident microglia by using more sensitive techniques (BrdU incorporation detected in Iba1^+^ cells by double immunohistochemistry of diaminobenzidine [DAB] and alkaline phosphatase [AP]) (Olmos-Alonso et al., 2016). We found that microglial proliferation rates in the adult brain were approximately ten times higher (Figures 3A–3C) than those previously reported by Lawson et al. (1992). On average, 0.69% of the total microglial cells were proliferating (Iba1^+^BrdU^+^) after a single pulse of BrdU. This rate was particularly high in the dentate gyrus (DG), the only area where we also found that aging had an impact on the proliferation rate (Figures 3A–3C). We ensured that these rates were not underestimated by the dose of BrdU, because we had performed a dose-response analysis of microglial proliferation confirming that the BrdU dose (7.5 mg/mL) used was optimal (Figures S2A and S2B). We next performed a time-course analysis of proliferation and division after a single pulse of BrdU in microglial cells (Figure 3B). In the cortex, we could detect the duplication of the proliferating population from 16 hr after the BrdU pulse, indicating successful cell-cycle exit and cell division (Figure 3B). Return to the baseline number of Iba1^+^BrdU^+^ cells was observed from 24 hr. Considering that the S phase of mammalian cells comprises ∼50% of the duration of the cell cycle, with G_2_/M only taking a few hours (Cameron and Greulich, 1963), this allows an estimate of a cell-cycle length (*Tc*) of 32 hr. This would be in agreement with reported cell-cycle lengths of macrophages, which vary depending on the differentiation stage from 20 to 40 hr (Kueh et al., 2013). If the S phase spans ∼50% of the cell-cycle length, our data from BrdU labeling would only detect half of the dividing population. This indicates that ∼1.38% of the population will be proliferating at a given time (*F* = fraction of cells in a cell cycle). If we use these rates to calculate the time needed for the entire rodent microglial population to renew (*X*), with the equation

$$X=\frac{100xTc}{F}$$

we can estimate that the population renews once every 2,318 hr (∼96 days), allowing as many as six cycles of complete renewal during an animal’s lifetime (average 21 months). However, these calculations are based on estimations of *Tc* and require further specific study.

The proliferative cycle was quicker in the DG, where the initial duplication returned to baseline before 24 hr (Figure 3B). In addition to revealing the higher proliferative activity of microglia in the DG, these data strongly suggest that microglial death must be tightly temporally and spatially coupled to proliferation to maintain the stable density of microglial cells, as discussed later.

Higher figures were observed when analyzing the proliferation of human microglia (on average, 2% of the microglial population proliferating at a given time), according to double staining of Iba1 and Ki67 (Figures 3D and 3E). This rate is 2.9 times higher than that observed for mice described earlier (0.69%). However, Ki67 expression is not directly comparable to BrdU incorporation. This difference might be explained by how Ki67 would label not only the S phase but also other cell-cycle phases except G_0_. This means the labeling of Ki67 is approximately two times higher than that of BrdU (Kee et al., 2002), which only labels the S phase, comprising ∼50% of the duration of the cell cycle (Cameron and Greulich, 1963). If cell-cycle length remains constant in mammals (32 hr, as noted earlier), this would allow an estimation of hundreds of cycles of complete renewal during a lifetime (average 80 years).

To further explore age-related changes in microglial proliferation, we studied the expression of genes related to the colony stimulating factor 1 receptor (CSF1R)-driven proliferative response (Gómez-Nicola et al., 2013). We found a significant reduction in the expression of *PU.1* and *IRF8* in aging brains and a non-significant trend toward a reduction in relevant genes like *CSF1*, *CSF1R*, *C/EBPα*, *CD34*, or *RUNX1* (Figure S3). To further address the significance of the CSF1R pathway in controlling microglial turnover, we administered young mice a diet containing GW2580, a specific CSF1R inhibitor previously shown to cause blockade of microglial proliferation, but not microglia survival (Gómez-Nicola et al., 2013, Uitdehaag et al., 2011, De Lucia et al., 2016, Olmos-Alonso et al., 2016), in contrast to the microglia-depleting effects caused by the CSF1R inhibitor PLX3397 (Elmore et al., 2014). Treatment with GW2580 for 3 months decreased the total number of microglial cells (PU.1^+^) by 17% (Figures 3F and 3G), supporting the relevance of the CSF1R pathway in controlling the homeostatic maintenance of microglial turnover.

To provide an independent method to validate our analysis of microglial proliferation in mice, we took advantage of the ability of γ-retroviral vectors to selectively transduce proliferating glial cells (Gomez-Nicola et al., 2014). We delivered an Eco-SFFV γ-retroviral vector driving the expression of mCherry to the lateral ventricle of CSF1R promotor (c-fms) EGFP mice, allowing diffusion to adjacent areas (cortex and striatum) due to the initially injected volume (5 μL) (Figure 3H). We analyzed the incorporation of Eco-SFFV-RV (retroviral vector) mCherry 3 days after injection to allow the expression of detectable levels of mCherry (Gomez-Nicola et al., 2014) and the potential visualization of pairs of cells before postdivision microglial death (Figure 3B). We found a limited number of microglial cells (EGFP^+^) expressing mCherry, presenting as typical microglial duplets (Figure 3I). The quantification of proliferating microglial cells (mCherry^+^EGFP^+^) offered a proliferation rate (Figure 3J) similar to that previously described by analyzing the incorporation of BrdU in Iba1 cells (Figure 3A), validating our previous findings.

For direct visualization of microglial turnover, we used chronic live imaging of the olfactory bulb microglia in CX_3_CR1^GFP/+^ mice, coupled to repeated blood vessel imaging (Figure S4A) (Kovalchuk et al., 2015). To control for potential interference of the implantation of the chronic window on the microglial behavior, mice were analyzed 3–4 weeks after surgery to allow initial inflammation to resolve. After this, imaged microglia were typical highly branched, CD11b^low^ and CD68^−^ (Figures S4B and S4C), and therefore considered surveillant microglia. Repeated live imaging of microglia allowed the identification of cell division (duplication) (Figure 3K) or death (disappearance) (see Figure 6A later) and defined the proliferation rate of microglia at 0.79% per day (Figure 3L), similar to the rate we found with Iba1/BrdU staining (Figure 3A). During the first 24 hr after division, paired microglia were found at a significantly closer distance than resident non-dividing microglia (Figure 3M), suggesting that these cells were generated from the same proliferating cell. During the following days, the cells migrated away from each other and reached cell-to-cell dispersion similar to the rest of the microglial population within 3–4 days (Figure 3N). These data confirm the high rates of microglial proliferation detected by Iba1/BrdU staining and suggest that the territories occupied by microglia change upon cell division, probably affecting the performance of local homeostatic functions.

Thus, using three independent lines of evidence, our data show that microglia proliferate in the adult mouse and human brain at a high rate, allowing several cycles of renewal of the whole population during the organism’s lifetime.

### Microglial Turnover Is Not Maintained by Nestin^+^ Precursors

**Figure 4:**
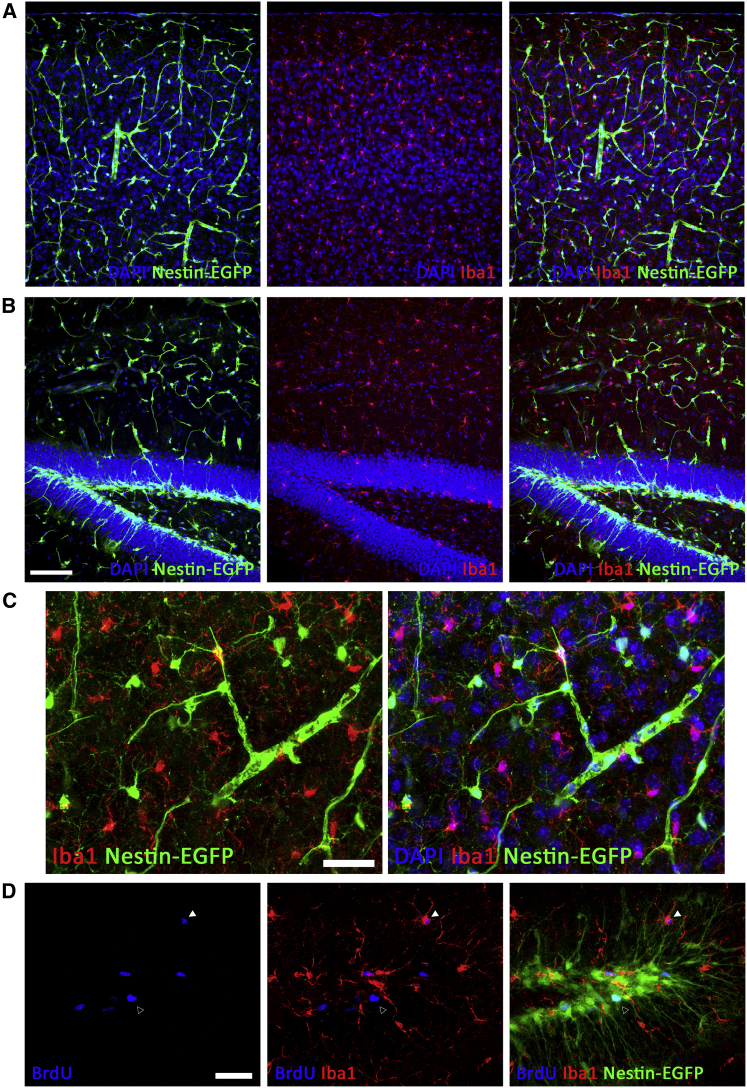
The Homeostatic Turnover of Microglia Is Not Maintained by Nestin^+^ Precursors (A–C) Immunofluorescent detection and confocal analysis of Iba1^+^ microglia (red) in nestin-EGFP (green) mice in the cortex (A and C) or hippocampal dentate gyrus (B). (D) Triple immunofluorescence for BrdU (blue), Iba1^+^ (microglia, red), and nestin-EGFP (green) in the dentate gyrus. An open arrowhead indicates a BrdU^+^Iba1^−^Nestin^+^ cell, while a white arrowhead indicates a BrdU^+^Iba1^+^Nestin^−^ cell. Scale bars are 50 μm in (A) and (B) and 20 μm in (C) and (D). n = 5.

In light of findings suggesting that Nestin^+^ microglial precursors may be involved in the repopulation response after pharmacological microglial ablation (Elmore et al., 2014) or transgenic microglial ablation (Bruttger et al., 2015), we aimed to study whether microglial proliferation in the steady state was maintained by a subpopulation of stem cell-like microglia. We analyzed Nestin-GFP mice as an optimal reporter mouse for the expression of nestin (Mignone et al., 2004), and although we showed the previously reported expression in pericytes (Figures 4A–4C), neural stem cells (Figure 4B), and oligodendrocyte precursor cells (Figures 4A and 4C) (Mignone et al., 2004), we did not find evidence of nestin expression in microglia (Iba1^+^) (Figures 4A–4C). We specifically studied the expression of nestin in proliferating microglia (Iba1^+^BrdU^+^; 24 hr postinjection) and found no evidence of nestin^+^ microglia (Figure 4D). We therefore conclude that microglial proliferation is not maintained by Nestin^+^ microglial precursors in the steady state.

### Microglial Proliferation and Apoptosis Are Temporally and Spatially Coupled to Maintain Microglial Homeostasis

**Figure 5:**
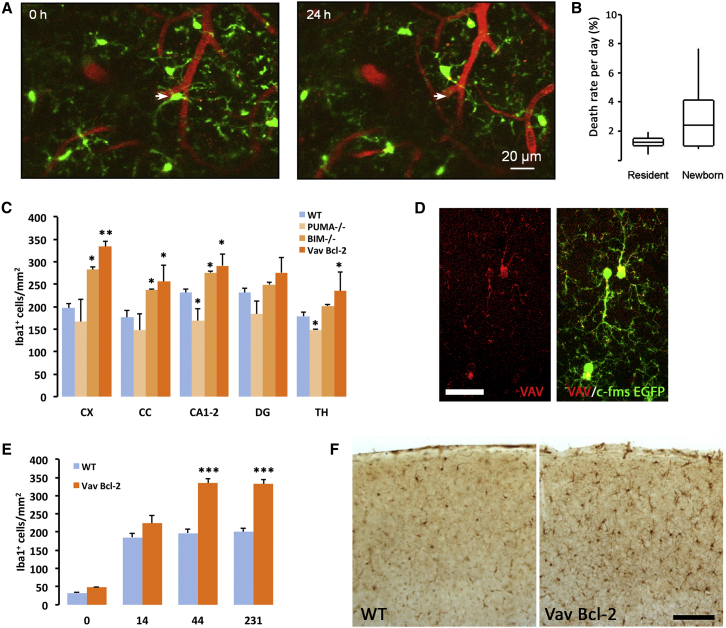
The Turnover of Microglia Is Balanced by Apoptosis (A) Maximal intensity projection (MIP) images of the same field of view (88–106 μm depth, 2 μm step) in a CX_3_CR1^GFP/+^ mouse. Arrows point to a disappearing (i.e., dying) microglial cell. (B) Death rate of microglia (median ± IQR; n = 669 cells, 9 FOVs, and 4 mice). (C) Microglial density across regions (CX, cortex; CC, corpus callosum; CA1–2, hippocampal CA1–CA2; DG, dentate gyrus; TH, thalamus) in wild-type (WT), PUMA^−/−^, BIM^−/−^, and Vav-Bcl2 mice. (D) Expression of Vav (red) in microglia (c-fms EGFP, green), analyzed by confocal microscopy. (E) Time-course analysis of postnatal (P0–P231) microglial density in wild-type (WT) and Vav-Bcl2 mice. (F) Representative example of microglial cells (Iba1^+^) in the cortex of WT and Vav-Bcl2 mice. Scale bars are 20 μm in (A) and (D) and 100 μm in (F). Data shown in (C) and (E) are represented as mean ± SEM. n = 4 WT mice, 3 PUMA^−/−^ mice, 4 BIM^−/−^ mice, and 7 Vav-Bcl2 mice (C); n = 4 (E). Statistical differences: ^∗^p < 0.05, ^∗∗^p < 0.01, ^∗∗∗^p < 0.001. Data were analyzed with a two-way ANOVA and a post hoc Tukey test (C and E).

The time-course analysis of microglial proliferation (Figure 3B) suggested that microglial cell death plays a key role in maintaining the stable number of microglia over time. Given the difficulties of analyzing microglial apoptosis by traditional methods (Sierra et al., 2013), we set out to address this point by using live imaging of microglia in CX_3_CR1^GFP/+^ mice. Under these conditions, microglial death is defined as a disappearance of a cell within the network of relatively immobile neighboring cells (Figure 5A), with the blood vessel pattern providing additional landmarks. The death rate of resident microglia was found to be 1.23% per day, while the death rate for newborn (recently divided) microglia was 2.40% (Figure 5B). For newborn microglial cells, the death rate was highest during the first 5 days after division (5.0% ± 3.5%), significantly higher than the death rate in the resident adult cell population.

To further study the relevance of microglial apoptosis in the maintenance of the population, we studied the numbers of microglia in three mouse models defective in intrinsic apoptosis (PUMA^−/−^, BIM^−/−^, and Vav-Bcl2). While the first two have ubiquitous deletion of the pro-apoptotic Bcl-2 homology domain 3 (BH3)-only molecules PUMA or BIM, the Vav-Bcl2 mice have the anti-apoptotic molecule Bcl2 overexpressed only in cells of the myeloid lineage. When compared to WT mice, both BIM^−/−^ and Vav-Bcl2 mice were found to have a significant increase in the number of microglial cells (Figure 5C). PUMA^−/−^ showed no difference, or even a reduction, in the number of microglia (Figure 5C), in agreement with previous findings in the eye (Zhang et al., 2012) and suggesting that microglial apoptosis is PUMA independent. Because Vav-Bcl2 mice provide a robust block in intrinsic apoptosis only in lymphoid and myeloid lineage cells (Egle et al., 2004), which in the brain is restricted to microglia, we decided to focus on the study of this model (Figure 5D). A time course of postnatal development of the microglial population in Vav-Bcl2 mice showed that the increase in the number of microglial cells is reached early in life (P44) and remains stable until middle age (before the onset of other health defects) (Figures 5E and 5F) (Egle et al., 2004). This stabilization of increased density, caused by deficient microglial apoptosis, is perhaps explained by the inability of the parenchyma to accommodate more cells, suggesting contact inhibition mechanisms are in place.

**Figure 6:**
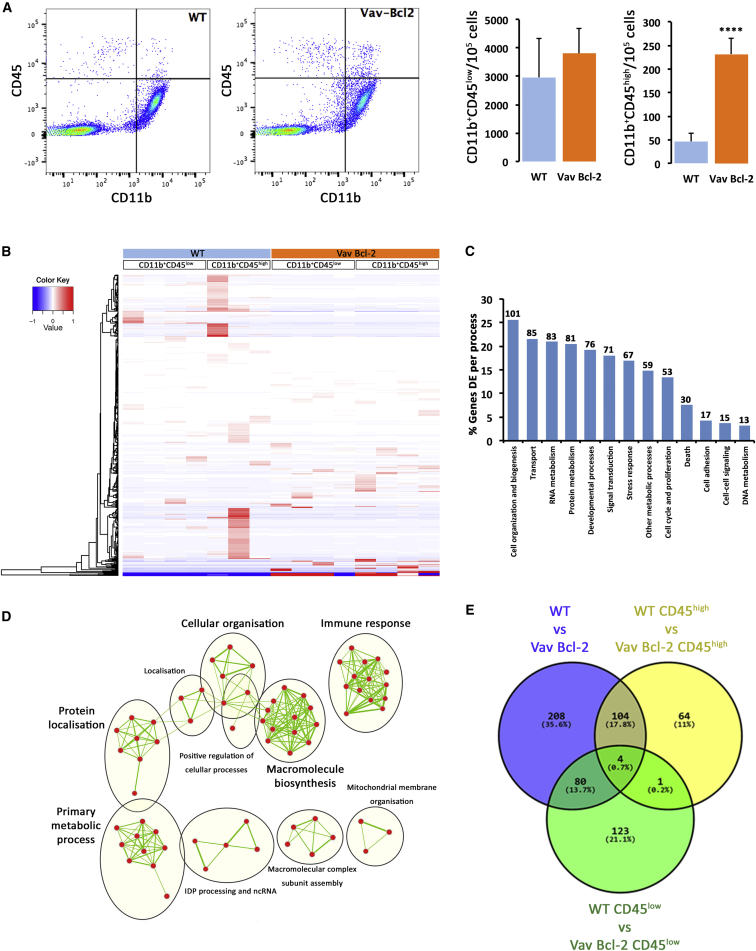
Transcriptomic Profiling of Microglia from WT and Vav-Bcl2 Mice (A) Flow cytometry analysis and sorting of microglia from WT and Vav-Bcl2 mice. Crosshair in flow cytometry analysis and sorting plots shows gating parameters used to define CD11b^+^CD45^low^ and CD11b^+^CD45^high^ subpopulations and subsequent sorting. Statistical differences: ^∗∗∗∗^p < 0.0001. Data were analyzed with a t test (A). (B) Heatmap representation of genes showing a significant (p < 0.01; >10-fold change) change in Vav-Bcl2 versus WT microglia (combined CD45^+^). Clustering of genes by expression profile is shown on the left. (C) Clustered representation (GO Slim) of GO processes significantly altered in Vav-Bcl2 compared to WT microglia. Number of genes altered per cluster is shown on top of the bars. (D) Enrichment map of GO terms, where red nodes represent GO terms and green edges represent shared genes (thicker lines indicate more shared genes). (E) Venn diagram representing the intersection of the transcriptional variability observed when comparing total (blue), CD45^low^ (green), or CD45^high^ (yellow) Vav-Bcl2 to WT microglia.

To understand the impact of apoptosis blockade on microglial phenotype, we isolated microglia by flow cytometry analysis and sorting (FACS) and analyzed their transcriptomic profile by RNA sequencing (RNA-seq) (Figure 6). Flow cytometry analysis showed a significant increase in the population of CD11b^+^CD45^high^ cells in Vav-Bcl2 mice when compared to WT littermates (Figure 6A), identifying this subpopulation of microglia as the biggest contributor to increased numbers observed previously. Isolation of CD11b^+^CD45^low^ and CD11b^+^CD45^high^ subpopulations from WT and Vav-Bcl2 followed by RNA-seq profiling rendered a total of 137 genes statistically (p < 0.01; fold change > 10) upregulated in Vav-Bcl2 versus WT and 259 genes statistically downregulated in Vav-Bcl2 versus WT microglia (Figure 6B; Table S1). Gene Ontology analysis revealed that differentially expressed genes were particularly associated with gene ontology (GO) Slim terms involved in metabolic processes and biogenesis (Figure 6C). Clustering of GO terms (based on genes shared between each GO category) revealed GO processes previously identified to be upregulated in microglia (Grabert et al., 2016), including immune response and macromolecule biosynthesis (Figure 6D). Vav-Bcl2 mice also showed a significant alteration of genes clustered under the processes of the cell cycle, proliferation, and death, confirming the expected effects of Bcl-2 upregulation (Figure 6C). Vav-Bcl2 microglia had significant repression (>200-fold) of the pro-apoptotic gene Bad and significant upregulation (17-fold) of the anti-apoptotic gene Api5 (Table S1). In addition, Vav-Bcl2 microglia showed significant repression of cell-cycle-promoting genes like Mad2l1, Mdm2, Cdca3, Cdk1, Cdc20, and Cdc20b (all > 25-fold downregulated) (Table S1). These changes confirm the anti-apoptotic effects of Bcl-2 overexpression in microglia but also suggest impaired cell-cycle regulation as an associated effect. A Venn diagram showing the relations between the gene sets of CD45^low^ and CD45^high^ identifies the CD11b^+^CD45^low^ subpopulation as the major contributor to the transcriptional variability observed between WT and Vav-Bcl2 microglia (Figure 6E).

Although Vav-Bcl2 mice had significant alteration in the number and phenotype of microglia through most of their adult life, they did not display gross deficiencies in the astrocyte populations (Figure S5A) or the neuronal populations (Figure S5B) and only showed minor differences in age-dependent changes in synaptic density (Figure S5C). Vav-Bcl2 mice showed no differences in behavioral performance when compared to WT mice (Figures S6A and S6B).

**Figure 7:**
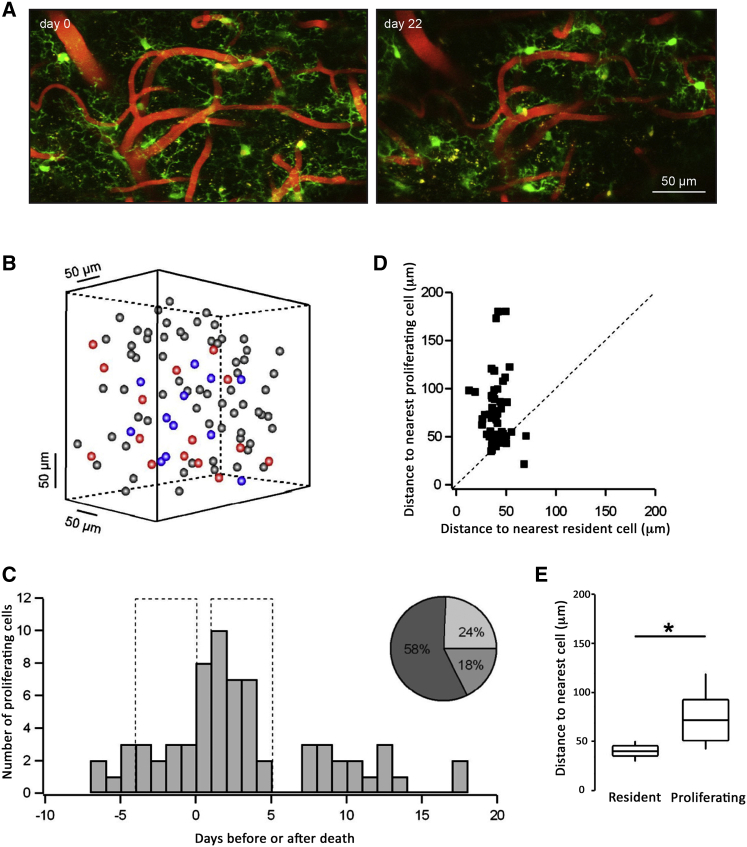
Temporal and Spatial Coupling of Microglial Proliferation and Death (A) MIP images of a sample field of view (50–80 μm depth, 1 μm step) in a CX_3_CR1^GFP/+^ mouse taken at the beginning (left, day 0) and at the end (right, day 22) of the imaging period. Bone growth occurred in the lower right corner of the latter image. (B) 3D matrix illustrating the history of cells in the sample field of view (317 × 317 × 160 μm) during the 22-day-long imaging period. Stable cells are shown in gray, cells that are going to die are shown in red, and cells that are going to divide are shown in blue. This FOV includes the cells shown in (A). (C) Temporal relationship between death and proliferation events (n = 68 cells, 9 FOVs, and 4 mice). The time when a cell dies is set as day 0 (reference point), and the relative time when proliferation occurs in its vicinity (≤200 μm) is calculated. The pie chart illustrates the fractions of cells proliferating in the vicinity of a dying cell 4 days before (light gray), during (gray), or 4 days after (dark gray) the death of the reference cell. (D) Spatial relationship between a dead cell and the nearest proliferating or resident cell (n = 53 dead cells, 9 FOVs, and 4 mice). (E) Summary of the data shown in (D) (median ± IQR; n = 53 cells, 9 FOVs, and 4 mice). Statistical differences: ^∗^p < 0.001, Wilcoxon signed-ranks test. Scale bar in (A) is 50 μm.

We next analyzed the temporal and spatial relationship between death and proliferation events in vivo. We observed rapid reorganization of the microglial landscape (Figure 7A). This is exemplified by the representation of the history of microglia during the 22-day-long imaging period in a sample field of view, with stable cells shown in gray, cells going to die shown in red, and cells going to divide shown in blue (Figure 7B). Microglial proliferation and apoptosis were spatially and temporally coupled, because many more cells proliferated in the vicinity (≤200 μm) of a dying cell immediately after its death (Figure 7C). Some of the proliferating cells were immediately adjacent to the dying cells, whereas others were located more distantly (Figures 7B and 7D). The median distance between the dying and the nearest proliferating cell was 72.95 ± 43.13 μm (n = 19 cell pairs), almost double the distance between the dying and the nearest resident cell (37.94 ± 11.74 μm, n = 31 cell pairs) (Figures 7D and 7E). Overall, proliferating cells were found to be the second-closest neighbors to dying cells.

In summary, our data indicate that the microglial population undergoes constant and rapid remodeling based on temporal and spatial coupling of proliferation and apoptosis, providing a mechanism for the homeostasis of the population through life. This constant renewal causes not only the individual cellular players to change but also their spatial layout to be rapidly modified.

## Discussion

During the last decade, the study of microglial cells and neuroinflammation has experienced a revolution. Minimally invasive methods have revealed microglia to be highly dynamic in their interaction with the microenvironment, responding to inflammatory signals (Nimmerjahn et al., 2005, Davalos et al., 2005) and interacting with neuronal circuits at the synaptic level (Tremblay et al., 2010, Wake et al., 2009). Microglia can sculpt the brain and affect its physiology, because they have been observed to contain phagocytic inclusions with features of axonal terminals, dendritic spines, or unneeded neuronal progenitors (Tremblay et al., 2010, Paolicelli et al., 2011, Sierra et al., 2010). Our data demonstrate that microglial cells are actively renewed and that the brain population is maintained by a finely tuned balance of proliferation and apoptosis.

It has long been assumed that microglia are long-lived cells. At the population level, microglia are long lived, but at the individual cell level, they are not. The microglial landscape changes radically within a few weeks, with cells dying, other taking their place, and their absolute position changing. This renewing landscape will likely influence the interpretation of phenomena such as microglial priming, in which the microglial response is exaggerated (stronger than that observed in stimulus-naive microglia) to a secondary insult. This is perhaps best illustrated when the first (priming) and second stimuli are separated by prolonged periods of time in the context of adult responses to early-life infections (Bilbo and Schwarz, 2009), delayed inflammation after traumatic brain injury (TBI) (Johnson et al., 2013), or the onset of age-related amyloid deposition after gestational inflammation (Krstic et al., 2012). There, microglial priming implies the need for a “microglial memory” of the first stimulus to elicit an exaggerated response to the second stimulus. We suggest that our findings could either support the hypothesis that microglial priming is achieved through epigenetic (inheritable) changes (Schwarz et al., 2011) or suggest that microglia memory could be stored elsewhere in the neural or glial network. We believe that the parameters defined here will stimulate a reinterpretation of many of the functions of microglia in health and disease.

That adult microglial population is maintained at least partly by self-renewal has been largely assumed for more than 2 decades, since our group reported on the proliferation of resident microglia (Lawson et al., 1992). Using [3H]-thymidine incorporation and detection with autoradiography, Lawson et al. (1992) described a very low turnover rate for microglia, with ∼0.05% of the cells dividing at a time. Although a probable underestimation, acknowledged at the time due to the relative insensitivity of the method, these studies were never revisited using the gold standard in the field: incorporation of BrdU. We now show, using three independent methods, that the proliferation index of microglia is much higher than expected (more than ten times higher), and an average of 0.69% microglial cells are in S phase at a given time. This rate would allow for an estimation of the brain’s microglial population being renewed every ∼95 days, allowing several cycles of renewal within the lifetime of a mouse. Higher rates are found in the human brain (average 2%), with a more dramatic consequence on the turnover cycles, leading to estimates that microglia would cycle hundreds of times during 80 years of life. However, the estimation of the turnover rate of human microglia would need alternative methods to provide a more accurate calculation and allow extrapolation to the average human population.

This homeostatic microglial proliferation is balanced by the opposing force of microglial apoptosis. The apoptotic cascade controlling microglial death seems to be dependent on the pro-apoptotic molecule BIM but not PUMA, as shown from the analysis of knockout (KO) mice. A report demonstrated that deficiency in PUMA leads to decreased numbers of both retinal and brain microglia because of unexpected roles of this protein in promoting cell survival (Zhang et al., 2012), consistent with our present findings showing a decreased density of microglia in PUMA^−/−^ mice. Microglial death can be counteracted by the inhibition of mitochondrial apoptosis as indicated by the overexpression of Bcl2, leading to increased microglial numbers. Surprisingly, our results support the hypothesis that the brain can only accommodate certain number of microglial cells, because deficient microglial death causes the increased microglial numbers to plateau after postnatal development. In the normal brain, the microglial population displays a mosaic-like organization in which processes of individual cells avoid contact with one another, being disrupted only with the emergence of changes related to pathology, age, or systemic influences (Gomez-Nicola and Perry, 2015). Transcriptomic profiling of microglia from Vav-Bcl2 mice highlights profound alterations of their functional profile, including altered metabolism and immune response, providing a link between homeostatic microglial apoptosis and phenotypic profile. A more detailed future study of the mechanisms by which altered microglia turnover could affect basic microglia functions, including those controlling their inflammatory properties, will provide valuable insights into understanding the maintenance of the microglial population in health and disease.

The sub-regional analysis of microglial turnover highlighted the DG as a particularly active anatomical region. In the DG, microglial proliferation is higher, is quicker, and decays more rapidly with age. An age-dependent decrease in proliferation is also observed in the population of DG neural progenitors (Kuhn et al., 1996), and this can be correlated with the decrease of microglial proliferation, because microglial phagocytosis of progenitors is coupled to neurogenic activity (Sierra et al., 2010). Thus, aging would lead to a decrease in DG microglial proliferation indirectly, through a decline in neurogenesis. A more direct effect of aging on microglia residing within the DG would imply microglial senescence. Replicative senescence, the loss of mitotic potential accompanied by significant telomere shortening, occurs once a cell has undergone approximately 50 replications, the so-called Hayflick limit (Hayflick, 1965). Thus, we hypothesize that the increased microglial turnover in the DG will lead to a quicker extinction of the proliferative capacity of these cells, as observed in our data. The microglial population at the DG seems particularly susceptible to telomere shortening, as highlighted using a mouse model of telomere dysfunction (telomerase RNA component [TERC] KO) (Khan et al., 2015), supporting the hypothesis that increased microglial division can lead to replicative senescence.

In an attempt to reconcile conflicting evidence about the contribution of circulating monocytes to the composition of the microglial population at perinatal stages (Ginhoux et al., 2013), we developed a cell-tracking approach based on the intra-liver tracing of embryonic hematopoiesis. Our results support the existence of a wave of monocytes that infiltrate the brain, peaking at P3, but indicate that these are rapidly depleted by apoptosis and do not contribute to the final microglial population, which we can now confirm is exclusively formed by yolk sac-derived progenitors. The elimination of infiltrated macrophages is coincident with a wave of microglial proliferation, followed by a further selection process before the final number of cells is achieved (Nikodemova et al., 2015). The functional significance of this wave of liver-derived monocytes is unknown, and should be the subject of future research, but the temporal coincidence with the refinement of the microglial numbers prompts speculation that these cells could trigger the death of a subpopulation of yolk sac-derived microglia.

The changes in microglial morphology that occur during aging are well documented; however, a particular morphological change that has received little attention to date is the formation of giant, multinucleated microglial aggregates in aged mice, such as those observed in our study. Previous studies have shown that microglia form aggregates with multiple nuclei, which can include more than 20 individual cells, under certain inflammatory conditions (Fendrick et al., 2007, Perry et al., 1993). Our data show that these aggregates are not generated by failed cytokinesis after division. Similar structures are observed in the context of a repopulation paradigm after genetic ablation of microglia (Bruttger et al., 2015). However, these clusters are BrdU^+^ (Bruttger et al., 2015), transient, and not fused, suggesting they serve as pools of repopulating microglia, whereas we here observe a fusion or aggregation of groups of cells. Given the territorial nature and lack of contact between microglial cells in younger brains, this is seen as an aberrant morphological development and represents a significant change in the phenotype and function of microglia in aging. With our data, we can provide clear evidence that these aggregates likely originate from the incorporation of circulating monocytes into the brain parenchyma, but further research is needed to fully understand their function.

One question raised by our data relates to the molecular regulation of the self-renewal process. We provide evidence for a necessary, but not sufficient, role of CSF1R in controlling microglial turnover in homeostasis, as shown from our mRNA studies and from the pharmacological inhibition of the CSF1R tyrosine kinase activity with GW2580. Elimination of microglia can be achieved by acute treatment with a highly potent CSF1R/c-kit/FLT3/PDGFRβ inhibitor, highlighting the relevance of these receptors in maintaining microglial numbers (Elmore et al., 2014). However, other systems may have to be in place to fully control microglial turnover. Recent studies, using genetic ablation of microglia, show that interleukin-1 receptor (IL-1R) plays a crucial role in the replenishment process (Bruttger et al., 2015). Although a repopulation process cannot be compared to the homeostatic maintenance of the microglial population, complementary systems (CSF1R, IL-1R, or others) must be in place to ensure the stability of this important population of non-neuronal cells in the CNS.

In light of the current data, we conclude that the turnover of the microglial population is a highly dynamic process, made possible by the finely tuned temporal and spatial balance of microglial proliferation and apoptosis. Our data question the view of microglia as a long-lived population, almost never renewed in the adult brain, and propose a more dynamic scenario, which will help uncover the key microglial functions in the healthy and the diseased brain.

## Experimental Procedures

### Experimental Mice

All experimental procedures were either approved by a local ethical review committee and conducted in accordance with personal and project licenses under the UK Animals (Scientific Procedures) Act (1986) or performed in accordance with institutional animal welfare guidelines and were approved by the government of Baden-Wurttemberg, Germany. Details of experimental mice can be found in the Supplemental Information.

To analyze cell proliferation, mice received injections of intraperitoneal (i.p.) BrdU (Sigma-Aldrich; 7.5 mg/mL, 0.1 mL/10 g weight in sterile saline). A dose-response experiment was performed using 3.75, 7.5, or 15 mg/mL BrdU.

### Postmortem Human Brain Samples

For immunohistochemical analysis, human brain autopsy tissue samples (n = 15 per group) (temporal cortex, paraffin-embedded, formalin-fixed, 96% formic acid-treated, 6 μm sections) from the National CJD Surveillance Unit Brain Bank were obtained from Alzheimer’s disease or variant Creutzfeldt-Jakob disease (vCJD) non-diseased age- and sex-matched young (age 20–35) or aged (age 58–79) controls, from whom consent for use of autopsy tissues for research had been obtained. Ethical permission for research on autopsy materials stored in the National CJD Surveillance Unit was obtained from Lothian Region Ethics Committee.

### In Utero Intra-liver Tracing of Embryonic Hematopoiesis

Cell tracking was performed by the administration of VSVG-SFFV-Venus or VSVG-SFFV-mCherry lentiviral vectors. Details on the design, production, and application of these vectors can be found in the Supplemental Information.

### Chronic Cranial Window Implantation

The chronic cranial window was installed as previously described (Kovalchuk et al., 2015). A detailed description of the method can be found in the Supplemental Information.

### Two-Photon Imaging

Microglia expressing EGFP in the olfactory bulb (OB) of CX_3_CR1^GFP/+^ mice were imaged once a day for 10 to 22 days by means of two-photon microscopy. Details of the method can be found in the Supplemental Information.

### Immunohistochemistry

Coronal hippocampal sections were cut from paraformaldehyde-fixed, frozen, or fresh brains. Mice perfusion, tissue processing, and immunohistochemical analysis were performed as previously described (Gómez-Nicola et al., 2013), with details found in the Supplemental Information.

### Statistical Analysis

Data were expressed as mean ± SEM and analyzed with the GraphPad Prism 5 software package. When normality and homoscedasticity assumptions were reached, we applied the two-tailed Fisher t test, one-way or two-way ANOVA, followed by the Tukey post hoc test for multiple comparisons. For the analysis of two-photon imaging for normally distributed data, mean ± SEM was calculated, and the Student’s t test was used for comparison of two groups. For data that were not normally distributed, median ± 1 interquartile range was presented as a boxplot, and 10 to 90 percentiles were shown as whiskers. For comparisons between non-parametrically distributed groups, we used the Mann-Whitney test. The Wilcoxon matched pairs test was used for nonparametric comparison of two paired groups. All statistical tests were two sided. Differences were considered significant for p < 0.05.

## Author Contributions

D.G.-N., V.H.P., and O.G. conceived and designed the study. K.A., Y.L., A.O.-A., F.G.-M., K.L., P.R., T.T., M.A.C., and D.G.-N. performed the in vivo experimental work and analyzed the experimental data. K.R., Z.M., A.S., S.B., M.S.C., O.G., and V.H.P. provided reagents, equipment, and/or experimental samples. D.G.-N. supervised the study and wrote the manuscript. All authors edited and approved the manuscript.

## Supplemental Information

Document S1. Supplemental Experimental Procedures and Figures S1–S6

Table S1. List of 396 Genes Differentially Expressed between Vav-Bcl2 and WT, Related to Figure 6

Document S2. Article plus Supplemental Information
