# Supplementary material for: Coupled Proliferation and Apoptosis Maintain the Rapid Turnover of Microglia in the Adult Brain
Source: Cell Rep. 2017 Jan 10;18(2):391–405. doi: 10.1016/j.celrep.2016.12.041 (PMC5263237; doi:10.1016/j.celrep.2016.12.041)
Supplement: Document S1. Supplemental Experimental Procedures and Figures S1–S6 [file mmc1.pdf]

Cell Reports, Volume *18*

Supplemental Information

Coupled Proliferation and Apoptosis Maintain the Rapid Turnover of Microglia in the Adult Brain

Katharine Askew, Kaizhen Li, Adrian Olmos-Alonso, Fernando Garcia-Moreno, Yajie Liang, Philippa Richardson, Tom Tipton, Mark A. Chapman, Kristoffer Riecken, Sol Beccari, Amanda Sierra, Zoltán Molnár, Mark S. Cragg, Olga Garaschuk, V. Hugh Perry, and Diego Gomez-Nicola

Supplemental figures

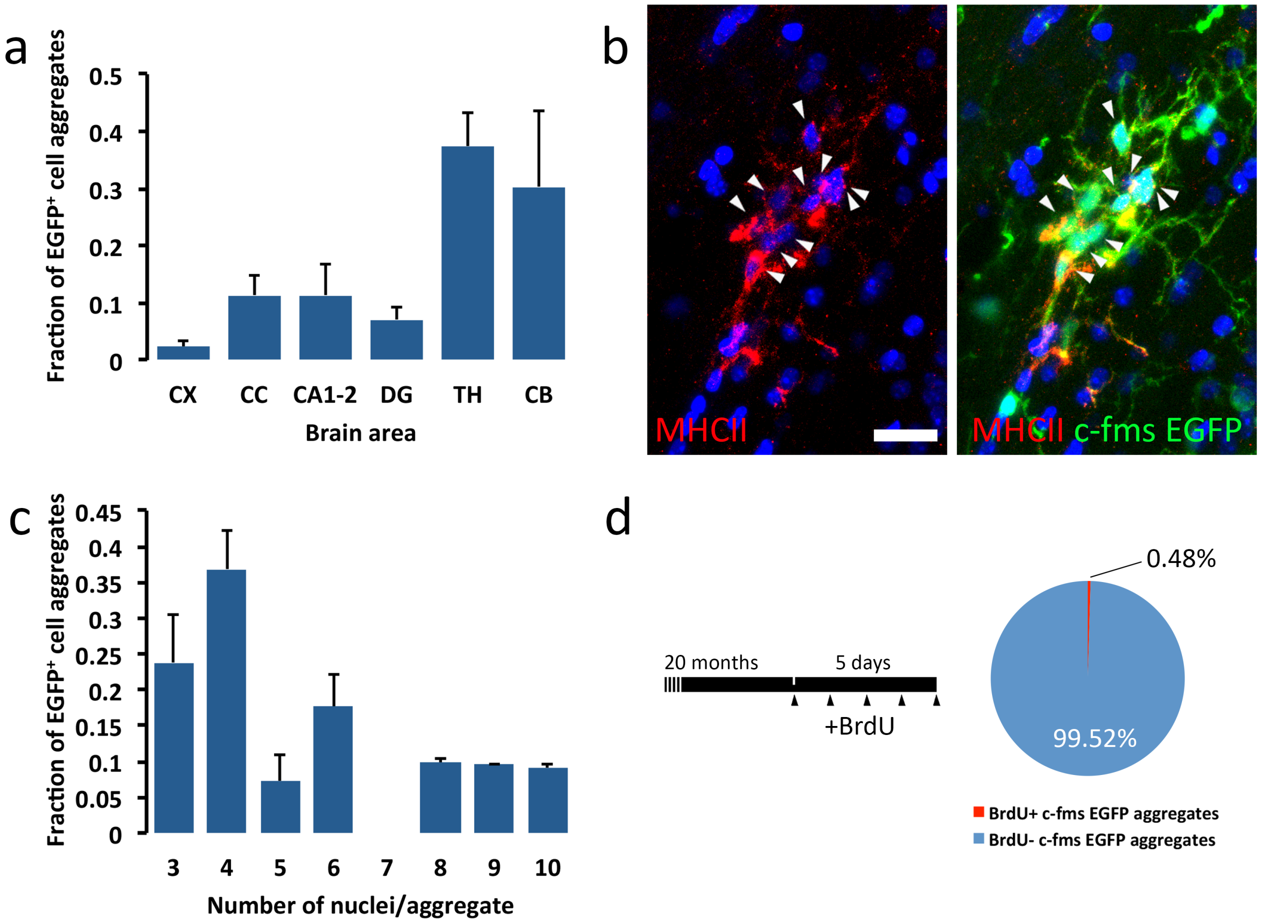

Figure S1 (related to Figure 2). Characterization of multinucleated microglial aggregates in the mouse aged brain.

(a) Distribution of microglial aggregates (c-fms EGFP^{+}) across brain regions (CX, cortex; CC, corpus callosum; CA1-2, hippocampal CA1-2; DG, dentate gyrus; TH, thalamus; CB, cerebellum) in aged (18-24 months) mice. **(b)** Expression of MHCII (red) in microglial aggregates (c-fms EGFP^{+}). Arrowheads indicate microglial nuclei. **(c)** Composition of the microglial aggregates (number of nuclei per aggregate). **(d)** Analysis of cell proliferation in microglial aggregates, after repeated dosing of BrdU (see experimental design). Scale bar in (**b**) 20mm.

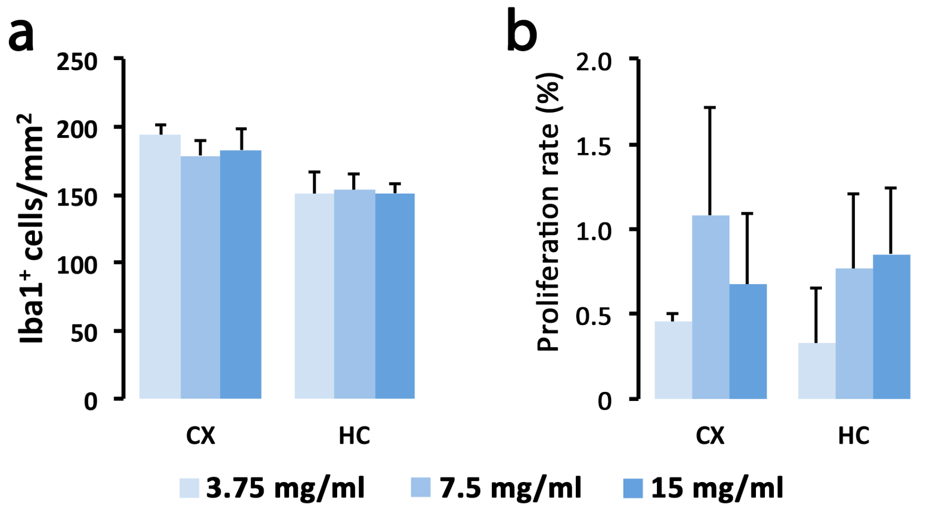

Figure S2 (related to Figure 3). Dose-response analysis of the incorporation of BrdU in microglial cells.

(a) Microglial density in the cortex (CX) or hippocampus (HC) after dosage with increasing doses of BrdU (3.75, 7.5 or 15 mg/kg). **(b)** Microglial proliferation ($Iba1^{+}BrdU^{+}$; proliferation rate, %) in the cortex (CX) or hippocampus (HC) after dosage with increasing doses of BrdU (3.75, 7.5 or 15 mg/Kg). N=4.

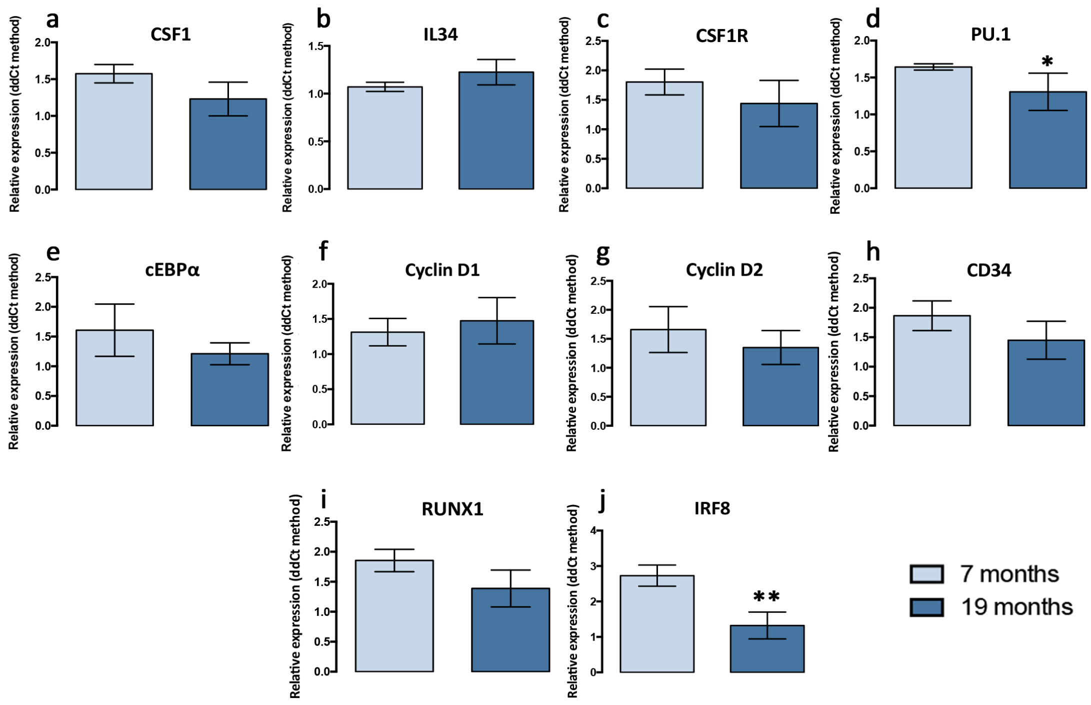

Figure S3 (related to Figure 3). Gene expression of the components of the CSF1R pathway and proliferation markers in the aged mouse brain.

(**a-j**) RT-PCR analysis of the mRNA expression of CSF1 **(a)**, IL34 **(b)**, CSF1R **(c)**, PU.1 **(d)**, C/EBPα **(e)**, Cyclin D1 **(f)**, Cyclin D2 **(g)**, CD34 **(h)**, RUNX1 **(i)** and IRF8 **(j)** in the brains of young (7 months) or aged (19 months) WT mice. Expression of mRNA represented as mean±SEM and indicated as relative expression compared to the housekeeping gene (GAPDH) using the 2-ΔΔCT method. Statistical differences: *$p<0.05$, **$p<0.01$. Data were analysed with a T-test. N=6.

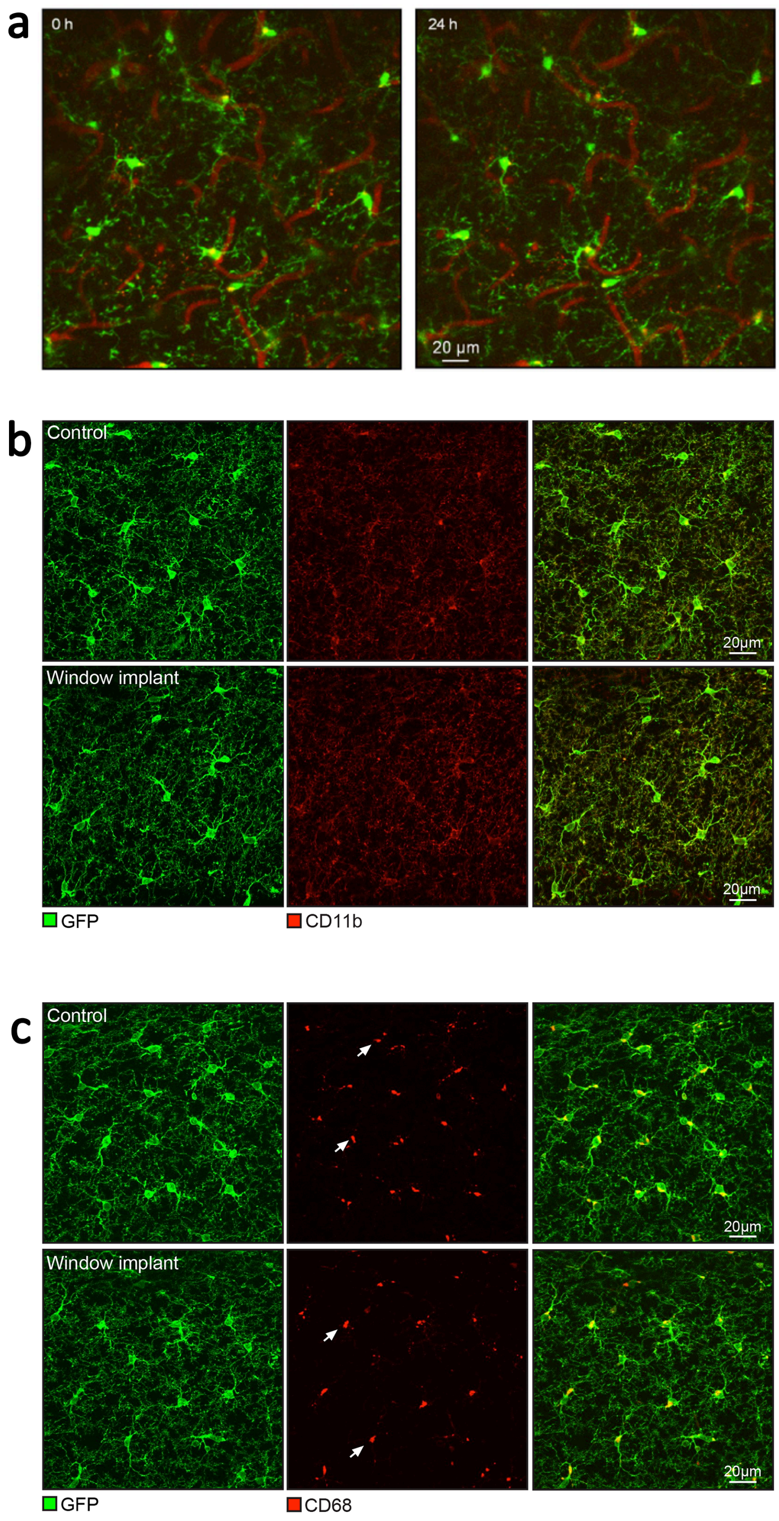

Figure S4 (related to Figure 3). Chronic in vivo imaging of olfactory bulb microglia.

(**a**) Maximal intensity projection (MIP) images of the same field of view (95 - 115 µm depth, step 1 µm) in the olfactory bulb of a $CX_3CR1^{GFP/+}$ mouse taken 24 hours apart. For repeated blood vessel imaging, sulforhodamine B (0.2 ml, 1 mM in PBS) was injected intraperitoneally at the beginning of each imaging session (as described in Kovalchuk et al., 2015). Scale bar 20mm. (**b, c**) Maximum intensity projection images (0-30 µm, 1 µm step) of the glomerular layer of the olfactory bulb in fixed slices obtained from control (upper panels) and window-implanted (lower panels)

$CX_3CR1^{GFP/+}$ transgenic mice. The GFP signal was enhanced by anti-GFP antibody and a secondary antibody AF 488 (green). Anti-CD11b (**a**) and anti-CD68 (**b**) antibodies are coupled to a secondary antibody AF 594 (red). White arrowheads point to CD68-positive lysosomes (Safaiyan et al., 2016). Images are representative for 20-30 fields of view imaged in 2 mice per group. All mice were littermates from the same litter. Scale bar is 20 µm.

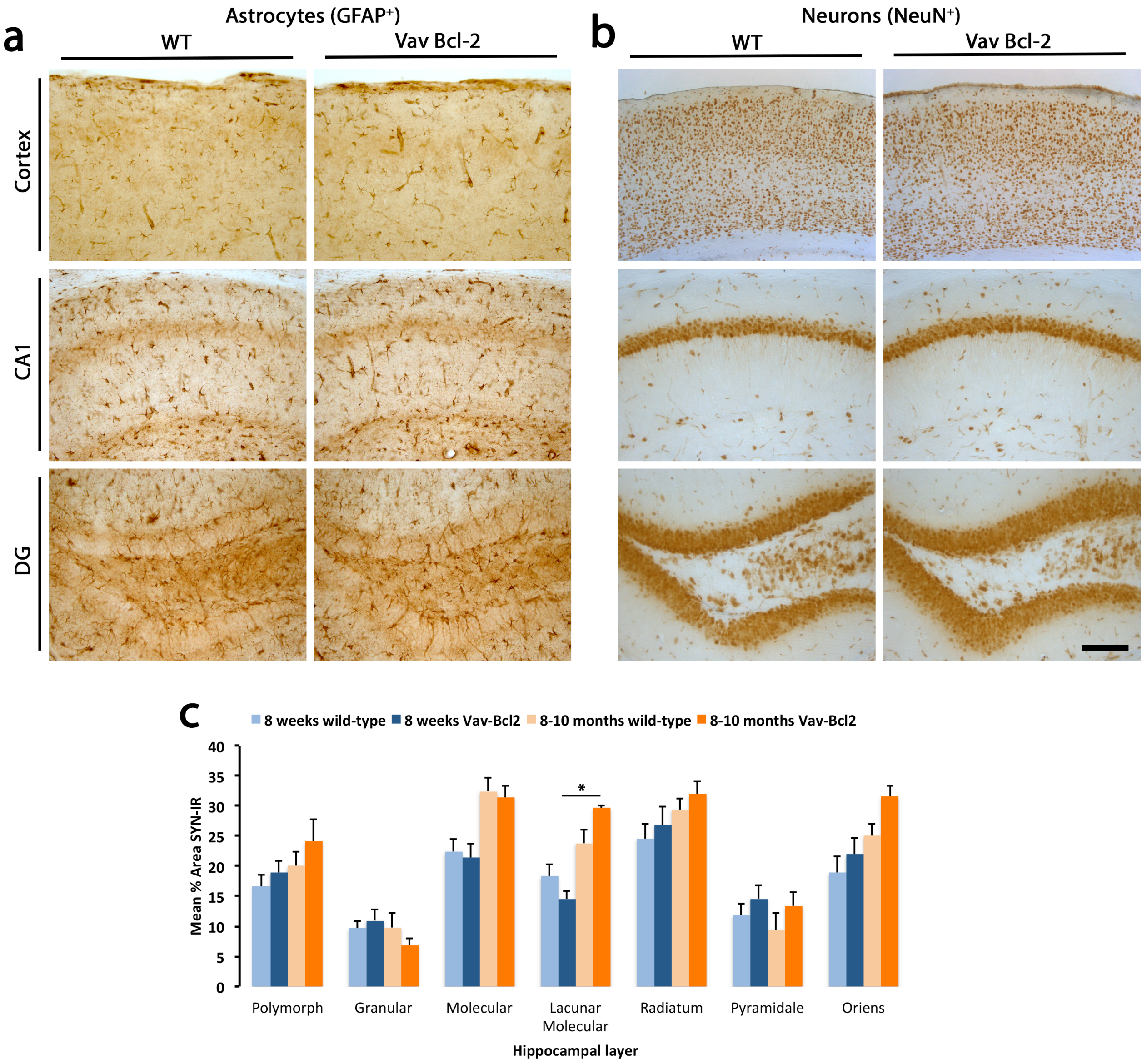

Figure S5 (related to Figure 5). Characterization of the astrocyte and neuronal populations in Vav-Bcl2 mice. **(a)** Analysis of the distribution and morphology of astrocytes ($GFAP^+$) in the cortex, hippocampal CA1-2 and dentate gyrus (DG) of wild-type and Vav-Bcl2 mice. **(b)** Analysis of the density and layering of neurons ($NeuN^+$) in the cortex, hippocampal CA1-2 and dentate gyrus (DG) of wild-type and Vav-Bcl2 mice. Scale bar in (**a, b**) 100mm (in **b**). (**c**) Immunohistochemical analysis and quantification of protein levels of synaptophysin in the hippocampus of WT and Vav-Bcl2 mice at 8 weeks and 8-10 months of age. Synaptophysin levels represented as mean±SEM of % $Synaptophysin^+$ area. Statistical differences: *$p<0.05$. Data were analysed with a two-way ANOVA and a post-hoc Tukey test. N=5.

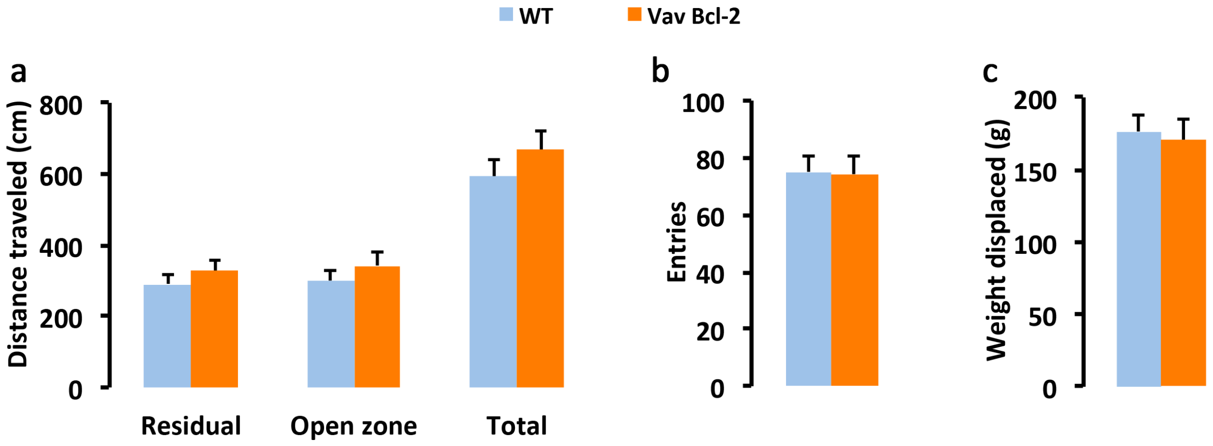

Figure S6 (related to Figure 5). Analysis of the behaviour of Vav-Bcl2 mice.

(**a, b**) Analysis of the behaviour in the open field, measured as total distanced travelled (**a**) or number of entries in the open zone (**b**) of WT and Vav-Bcl2 mice. Exploratory activity was measured as distance travelled (cm) in the open field test, analysing the locomotor activity in an open zone vs residual zone as a correlate of anxiety. (**c**) Burrowing behaviour, a measure of anhedonic behaviour, was measured as weight displaced (g) out of the tube in 24h. N=6

Table S1 (related to Figure 6). List of 396 genes differentially expressed between Vav Bcl-2 and WT.

logFC = log fold change (where positive values indicate VAV expression upregulated relative to WT and negative values indicate VAV expresssion downregulated). logCPM = log counts per million. P value - raw P value, FDR = False discovery rate corrected P value.

Supplemental Experimental Procedures

Experimental mice

C57BL/6, c-fms EGFP (macgreen)(Sasmono et al., 2003), $CCR2^{-/-}$(Menzies et al., 2012), $PUMA^{-/-}$ (Villunger et al., 2003), $BIM^{-/-}$ (Bouillet et al., 1999) and Vav Bcl2 mice (Egle et al., 2004) were bred and maintained at the local facilities of the University of Southampton (UK), while nestin-EGFP (Encinas et al., 2006) were maintained at the University of the Basque Country EHU/UPV (Leioa, Spain). Young (4-6 months) and aged (18-24months) mice were used in this study. For *in utero* intra-liver tracing experiments adult C57BL/6 mice were obtained from a local breeding colony at the University of Oxford (based on the Harlan (UK) strain). The day when vaginal plug was detected was referred to as E0. For live imaging experiments, we used 8 to 12 month-old $CX_3CR1^{GFP/+}$ mice (Jung et al., 2000), bred and maintained at the University of Tübingen (Germany).
All mice were maintained on a 12/12-hour light/dark cycle (7 AM, lights on) and provided with *ad libitum* access to food and water.
For the evaluation of the effects of treatment with GW2580 mice were 6 months of age when treatment began (N=6). Mice were fed with a control diet (RM1) or a diet containing GW2580 (Modified LabDiet® PicoLab EURodent Diet 14%, 5L0W (5LF2) with 0.1% (1000 ppm) GW2580 (LC Laboratories); TestDiet) for 3 months.
Sample size for each experiment was determined after performing power calculations, in order to achieve a significant difference of $p<0.05$, in light of a retrospective analysis of our previous results, to reach a power between 0.80-0.90, depending on the specific experimental conditions. The calculations are the customary ones based on normal distributions and were performed following statistical advice from the Research design and methodology Department of the University of Southampton.
The experiments were designed in compliance with the ARRIVE guidelines, including control groups for all experiments, randomizing the procedures and applying double-blinded analysis when possible.

***In utero* intra-liver tracing of embryonic haematopoiesis**

Design, production and application of the vectors was performed as previously described in detail(Gomez-Nicola et al., 2014) and derives from the previously described lentiviral gene-ontology vectors (LeGO (Weber et al., 2008)). Vector maps and sequence data for all vectors are available upon request; more information can be found at http://www.LentiGO-Vectors.de. Viral vectors were prepared with fast green, to allow visualization upon delivery. Exposure of living embryos was performed as previously described for *in utero* electroporation (Garcia-Moreno et al., 2014). Briefly, E14 pregnant mice were anesthetized by inhalation of isofluorane administered in conjunction with 100% oxygen. After midline laparotomy, the uterine horns were exposed out of the abdominal cavity and constantly warmed

and hydrated with pre-warmed PBS. The embryos were trans-illuminated. Embryonic livers were distinguished by their characteristic dark brown colour in the embryos' abdomen and injected with a glass electrode until fast green solution was visible. Each embryo was injected with a volume less than 1 µl comprising the mixture of viral particles (titer 10^9 particles/mL). In control experiments, the viral solution was injected in the amniotic sac, observing no subsequent cell labelling in the brain. Buprenorhpine (vetergesic; 0.05 mg/kg) and meloxicam (metacam; 0.2 mg/kg) were administered to the pregnant mice prior to and after surgery, respectively. Injected embryos were examined at different postnatal stages (P0, P3, P6, P21, P43), focusing our analysis on the cortex, hippocampus and cerebellum.

Chronic cranial window implantation

Briefly, mice were anaesthetized through intraperitoneal injection (i.p.) of ketamine/xylazine (80/4 µg/g body weight, BW, FAGRON, Barsbuettel, Germany, and Sigma, Sigma-Aldrich, St Louis, MO, respectively). Anesthetic depth was monitored by toe pinch throughout the surgery and additional ketamine/xylazine (40/2 µg/g of BW) was injected when necessary. Dexamethasone (2 µg/g of BW) was administered i.p. before the surgery and lidocaine (50 µl, AstraZeneca, Wedel, Germany) was applied subcutaneously before removing the scalp over the OB. The skull covering both OBs was cleaned and dried. A round groove (3 mm in diameter) was made with a microdrill over the two hemispheres of the OB through repeated drilling. Then the skull overlying both OBs was carefully removed, leaving the dura intact. The surface of the dura was rinsed with sterile extracellular solution (125 mM NaCl, 4.5 mM KCl, 26 mM $NaHCO_3$, 1.25 mM NaH_2PO_4, 2 mM $CaCl_2$, 1 mM $MgCl_2$ and 20 mM glucose, pH 7.4) and was then covered with a 3 mm glass coverslip (Warner Instruments, Hamden, CT). The margin between the coverslip and the skull was sealed with cyanoacrylate glue. The remaining exposed area over the OBs was covered with dental cement. Postoperative care included an analgesic dose of carprofen (5 µg/g of BW) for 3 days subcutaneously and the antibiotic baytril (Bayer, Leverkusen, Germany; 1:100 v/v) in drinking water for 10 days. After the surgery, animals were allowed to recover for at least 3 weeks and were subsequently examined for window clarity. For mice with good quality windows, a metal bar for head fixation was glued to the caudal part of the skull with dental acrylic and cement.

To assess the impact of chronic window implantation on the microglial phenotype, we performed staining on free-floating cryoslices (thickness 50 µm) at room temperature. The sections were treated with a blocking solution (5% normal donkey serum and 1% Triton-X 100 in PBS) for 2h to prevent non-specific background staining. After blocking the slices were exposed overnight to primary antibodies diluted in the blocking solution as indicated below. The following primary antibodies were used: anti-GFP (1:1000; Rockland), anti-CD11b (1:1500; AbD SeroTec), anti-CD68 (1:1000; AbD SeroTec). Thereafter the sections were rinsed in PBS three times for 10 min and incubated with AF 488- and AF 594-conjugated secondary antibodies (1:1000 in PBS +2% bovine serum albumin; Invitrogen) for 2h in

darkness. Afterwards, the sections were washed three times in PBS, transferred to Superfrost Plus charged glass slides (Langenbrink) and mounted in Vectashield Mounting Medium (Vector Laboratories).

Tracing of proliferation with γ-retroviral vectors

The delivery of Eco-SFFV mCherry γ-retroviral vectors was used to trace microglial proliferation in macgreen mice. The viral vector is a derivative of RSF91.GFP.pre* (Schambach et al., 2006), design and production was performed as previously described(Gomez-Nicola et al., 2014). Mice were anaesthetised with a ketamine/rompun mixture (85 and 13 mg/kg), and 5µl (10^9 particles/ml) of the viral particles were injected stereotaxically in the lateral ventricle at the following coordinates from bregma: anteroposterior, −0.1 mm; lateral, ±0.9 mm; depth, -2.2 mm.

Two-photon Imaging

Mice with cranial windows were anesthetized with isofluorane (2.5% in oxygen) and placed on a warming pad. The head of the mouse was fixed to X–Y microscope stage. During the experiment, concentration of isoflurane was between 0.8 and 1.5% enabling a breathing rate at ~100 breaths/min. The body temperature of the animal was kept between 36 and 37°C. Two-photon imaging was performed with a two-photon laser-scanning microscope (Olympus Fluoview 1000, Olympus, Tokyo, Japan) coupled to a Mai Tai Deep See Laser (Spectra Physics, Mountain View, CA). The emitted light was collected with a Zeiss ×20 water-immersion objective (NA 1.00). For precise cell identification across imaging sessions, angiography was performed through i.p. injection of a fluorescent dye sulforhodamine B (1mM in PBS, 0.1ml per 10g of BW, Sigma-Aldrich). 900nm excitation light (with laser power of 10-40 mW on the top of the specimen) was used for simultaneous excitation of eGFP and sulforhodamine B. The emission signals were split by a 570 nm dichroic mirror.

Behavioural tests

Vav Bcl2 or WT mice were tested on behavioural tasks at 4 months of age: open-field locomotor and exploratory activity and burrowing activity (anhedonic behaviour). The open-field tests were carried out using activity monitor software (Med Associated Inc.). These tests were performed as previous described (Gomez-Nicola et al., 2013).

Immunohistochemistry

We used the following primary antibodies: rabbit anti-Iba1 (Wako), mouse anti-human Ki67 (Dako), mouse anti-BrdU (DSHB), goat anti-Vav (Santa Cruz Biotechnologies), chicken anti-GFP/Venus (Aves Labs), rabbit anti-cleaved caspase 3 (Millipore), rat anti-MHCII (EBioscience), rabbit anti-GFAP (Dako), rabbit anti-Olig2 (Santa Cruz Biotechnologies), rabbit anti-NG2 (Millipore), rat anti-CD206 (AbD Serotec), mouse anti-NeuN (Millipore), mouse

anti-synaptophysin (SY38; Merck Millipore) and rabbit anti-PU.1 (Cell Signalling). Following primary antibody incubation, the sections were washed and incubated with the appropriate biotinylated secondary antibody (Vector Labs), and/or with the appropriate Alexa 405, 488 or 568 conjugated secondary antibody or streptavidin (Molecular Probes). For co-labelling of Iba1/Ki67 or Iba1/BrdU, following primary antibody sections were incubated with an anti-rabbit biotinylated secondary antibody (for Iba1 detection) and the ImmPRESS-AP Anti-Mouse (alkaline phosphatase) Polymer Detection Kit (for Ki67 or BrdU detection). For light microscopy, the sections were visualized using diaminobenzidine (DAB) precipitation or BCIP/NBT AP reaction, in a Leica CTR 5000 microscope, coupled to a Leica DFC300FX microscope camera. For PU.1 visualization, the DAB signal was enhanced with 0.05% Nickel Ammonium Sulfate, producing a black precipitate. After immunofluorescence labelling, nuclei were visualized by DAPI staining and the sections were mounted with Mowiol/DABCO (Sigma-Aldrich) mixture. The sections were visualized on a Leica TCS-SP5 confocal system, coupled to a Leica CTR6500 microscope.

The general immunohistochemistry protocol was modified for the detection of BrdU, adding a DNA denaturation step with 2N HCl (30min, 37°C), as previously described(Gomez-Nicola et al., 2013).

The protocol used for immunohistochemistry on human sections was a modification of the general protocol (DAB+AP), with antigen unveiling in citrate buffer being performed for 25min, as previously described(Olmos-Alonso et al., 2016).

Analysis of gene expression by RT-PCR

Young or aged mice WT mice (n=8/group) were processed to obtain samples from the hippocampus by dissection under a microscope, after intracardiac perfusion with heparinized 0.9% saline. RNA was extracted using Trizol (Life Technologies), quantified using Nanodrop (Thermo Scientific), to be retrotranscribed using the iScript cDNA Synthesis Kit (Bio-Rad), following manufacturer's instructions, after checking its integrity by electrophoresis in a 2% agarose gel. cDNA libraries were analysed by qPCR using the iTaq Universal SYBR Green supermix (Bio-Rad) and the following custom designed gene-specific primers (Sigma-Aldrich): csf1 (NM_007778.4; FW, agtattgccaaggaggtgtcag, RV, atctggcatgaagtctccattt), il34 (NM_001135100.1; FW, ctttgggaaacgagaatttggaga, RV, gcaatcctgtagttgatggggaag), csf1r (NM_001037859.2; FW, gcagtaccaccatccacttgta, RV, gtgagacactgtccttcagtgc), pu.1 (NM_011355.1; FW, cagaagggcaaccgcaagaa, RV, gccgctgaactggtaggtga), c/ebpa (NM_007678.3; FW, agcttacaacaggccaggtttc, RV, cggctggcgacatacagtac), runx1 (NM_001111021; FW, caggcaggacgaatcacact, RV, ctcgtgctggcatctctcat), irf8 (NM_008320; FW, cggggctgatctgggaaaat, RV, cacagcgtaacctcgtcttc), cd34 (NM_133654.3; FW, gccctacaggagaaaggctgggt, RV, gcccctcgggtcacattggc), cyclin D1 (NM_007631.2; FW, ggctcctctcatggcgctgc, RV, gtggcatgcacaacaggccg) and cyclin D2 (NM_009829; FW, tcgatgggctgcgttgcgtt, RV, gggagcctgcgtcaaagggg). Quality of the primers and the PCR reaction were evaluated by electrophoresis in a 1.5% agarose gel, checking the PCR product

size. Data were analysed using the 2-ΔΔCt method with Primer Opticon 3 software, using gapdh (NM_008084.2; FW, tgaacgggaagctcactgg, RV, tccaccaccctgttgctgta) as a housekeeping gene.

Microglial Fluorescent activated cell sorting (FACS) and RNAseq

Mice were terminally anaesthetised with an overdose of sodium pentobarbital and transcardially perfused with 0.9% saline solution. Brains were harvested and dissociated mechanically followed by enzymatic digestion using the MACS Neural Tissue Dissociation Kit (Miltenyi Biotech) then passed through a cell strainer of 70μm mesh (BD2 Falcon) with FACS buffer (PBS with 1% Fetal Bovine Serum (FBS) and 1mM EDTA (Sigma Aldrich)). The cell suspension was separated by 37% Percoll gradient centrifugation at 500 g for 30 min at 18°C (no brake).

Samples were immunostained with primary antibodies directed against CD11b (clone: M1/70) and CD45 (clone: 30-F11) (BD Biosciences) at 1:500 dilution at 4°C for 30 minutes. Cells were washed then analysed and sorted using a FACS Aria II (BD Biosciences). Data were acquired with FACSDiva software (Becton Dickinson). Post acquisition analysis was performed using FlowJo software version 10.2 (Tree Star).

cDNA synthesis from small pools of cells was performed at the Oxford Genomics Centre (Wellcome Trust Centre for Human Genetics) following the Smart-seq2 method (Picelli et al., 2013) and libraries prepared using Nextera XT (Illumina) with 0.25ng cDNA input and 12 PCR cycles. All libraries were pooled and sequenced on one lane HiSeq4000 at 75bp paired end. Data have been uploaded to the NCBI Short Read Archive under project PRJNA356215. Adapter-trimmed paired fastq reads were aligned to the mouse genome (GRCm38) using Hisat (Kim et al., 2015) with option --dta (increasing the stringency of transcript anchors) and outputted to a bam file using samtools (ver 1.1; (Li et al., 2009)). MarkDuplicates in Picard (https://broadinstitute.github.io/picard/) was used to identify and remove duplicate reads and featureCounts (Liao et al., 2014) within the Subread package (Liao et al., 2013) was used to provide gene counts. Gene counts from each of the libraries were combined and used to investigate differential expression (DE) with edgeR (Robinson et al., 2010). Comparisons were made between all WT and all VAV mice as well as between WT ($CD45^{high}$) and VAV ($CD45^{high}$), and WT ($CD45^{low}$) and VAV ($CD45^{low}$) and genes with a P value < 0.01 and a fold change of >10 were considered significantly DE. Applying an FDR considerably limited the number of genes identified as significantly DE, probably because of the low sample size. This was deemed too strict given our goal was to identify GO pathways which were affected in the VAV mice and was not to identify individual genes. Heatmaps were generated using the script *analyze_diff_expr.pl* in Trinity (Haas et al., 2013) and a matrix of TMM-normalised counts. Gene Ontology (GO) terms over-represented in the list of DE genes were identified using the MGI Gene Ontology Term Finder (http://www.informatics.jax.org/gotools/MGI_Term_Finder.html). Clustering of GO terms was created using Enrichment map v 2.1 (Merico et al., 2010) in Cytoscape 3.4.0 (Cline et al., 2007).

Image Analysis

For the analysis of two-photon imaging experiments Fluoview 3.0 Viewer (Olympus) was used to view stacks from the same FOV. Angiography through sulforhodamine B provides a stable landmark to distinguish immobile cells from cells that are new-born or gone. The death and proliferation rate in each FOV was presented as values normalized by the total number of cells and the number of days of observation in the same FOV. The centre to centre distance between two microglia cells was calculated by 3D ROI manager (Ollion et al., 2013) plugin of ImageJ software (http://rsb.info.nih.gov/ij/, National Institutes of Health). The centre-to-centre distance between the twin cells w was measured from the first day of the separation of the two cell bodies. The 3D matrix of the sample FOV showing the starting position of each microglia cell was generated by TrakEM2 (Cardona et al., 2012) ImageJ plugin.

For the analysis of Venus$^+$ cells in the postnatal brain (cortex, hippocampus and cerebellum), all cells were counted in a minimum of 5 sections per mouse, n=4 mice per groups. For the histological methods, the quantification of antigen positive cells (i.e. Iba1$^+$) in the different areas (n=4 fields/mouse, n=4-8 mice/group) was performed after DAB immunohistochemistry. The number of double positive cells (i.e. Iba1$^+$BrdU$^+$) in the specific area (n=4 fields/mouse, n=4-8 mice/group) was performed after double immunohistochemistry with DAB-AP. Data were represented as number of positive cells/mm^2. The quantification of antigen positive cells (i.e. Iba1$^+$Ki67$^+$ or Iba1$^+$) in human brains was performed in the white or grey matter of the temporal cortex after DAB/AP immunohistochemistry (n=20 fields/brain, n=9-10 brains/group). The proliferative rate of microglia was calculated as the % Iba1$^+$/BrdU$^+$ cells out of the total number of Iba1$^+$ cells. The quantification of the intensity of signal (i.e. synaptophysin) was performed after immunofluorescence, and presented as % stained area. All quantifications were performed with the help of the ImageJ image analysis software.

Supplemental references

BOUILLET, P., METCALF, D., HUANG, D. C., TARLINTON, D. M., KAY, T. W., KONTGEN, F., ADAMS, J. M. & STRASSER, A. 1999. Proapoptotic Bcl-2 relative Bim required for certain apoptotic responses, leukocyte homeostasis, and to preclude autoimmunity. *Science,* 286**,** 1735-8.

CARDONA, A., SAALFELD, S., SCHINDELIN, J., ARGANDA-CARRERAS, I., PREIBISCH, S., LONGAIR, M., TOMANCAK, P., HARTENSTEIN, V. & DOUGLAS, R. J. 2012. TrakEM2 software for neural circuit reconstruction. *PLoS One,* 7**,** e38011.

CLINE, M. S., SMOOT, M., CERAMI, E., KUCHINSKY, A., LANDYS, N., WORKMAN, C., CHRISTMAS, R., AVILA-CAMPILO, I., CREECH, M., GROSS, B., HANSPERS, K., ISSERLIN, R., KELLEY, R., KILLCOYNE, S., LOTIA, S., MAERE, S., MORRIS, J., ONO, K., PAVLOVIC, V., PICO, A. R., VAILAYA, A., WANG, P. L., ADLER, A., CONKLIN, B. R., HOOD, L., KUIPER, M., SANDER, C., SCHMULEVICH, I., SCHWIKOWSKI, B., WARNER, G. J., IDEKER, T. & BADER, G. D. 2007. Integration of biological networks and gene expression data using Cytoscape. *Nat Protoc,* 2**,** 2366-82.

EGLE, A., HARRIS, A. W., BATH, M. L., O'REILLY, L. & CORY, S. 2004. VavP-Bcl2 transgenic mice develop follicular lymphoma preceded by germinal center hyperplasia. *Blood,* 103**,** 2276-83.

ENCINAS, J. M., VAAHTOKARI, A. & ENIKOLOPOV, G. 2006. Fluoxetine targets early progenitor cells in the adult brain. *Proc Natl Acad Sci U S A,* 103**,** 8233-8.

GARCIA-MORENO, F., VASISTHA, N. A., BEGBIE, J. & MOLNAR, Z. 2014. CLoNe is a new method to target single progenitors and study their progeny in mouse and chick. *Development,* 141**,** 1589-98.

GOMEZ-NICOLA, D., FRANSEN, N. L., SUZZI, S. & PERRY, V. H. 2013. Regulation of microglial proliferation during chronic neurodegeneration. *J Neurosci,* 33**,** 2481-93.

GOMEZ-NICOLA, D., RIECKEN, K., FEHSE, B. & PERRY, V. H. 2014. In-vivo RGB marking and multicolour single-cell tracking in the adult brain. *Sci Rep,* 4**,** 7520.

HAAS, B. J., PAPANICOLAOU, A., YASSOUR, M., GRABHERR, M., BLOOD, P. D., BOWDEN, J., COUGER, M. B., ECCLES, D., LI, B., LIEBER, M., MACMANES, M. D., OTT, M., ORVIS, J., POCHET, N., STROZZI, F., WEEKS, N., WESTERMAN, R., WILLIAM, T., DEWEY, C. N., HENSCHEL, R., LEDUC, R. D., FRIEDMAN, N. & REGEV, A. 2013. De novo transcript sequence reconstruction from RNA-seq using the Trinity platform for reference generation and analysis. *Nat Protoc,* 8**,** 1494-512.

JUNG, S., ALIBERTI, J., GRAEMMEL, P., SUNSHINE, M. J., KREUTZBERG, G. W., SHER, A. & LITTMAN, D. R. 2000. Analysis of fractalkine receptor CX(3)CR1 function by targeted deletion and green fluorescent protein reporter gene insertion. *Mol Cell Biol,* 20**,** 4106-14.

KIM, D., LANGMEAD, B. & SALZBERG, S. L. 2015. HISAT: a fast spliced aligner with low memory requirements. *Nat Methods,* 12**,** 357-60.

LI, H., HANDSAKER, B., WYSOKER, A., FENNELL, T., RUAN, J., HOMER, N., MARTH, G., ABECASIS, G., DURBIN, R. & GENOME PROJECT DATA PROCESSING, S. 2009. The Sequence Alignment/Map format and SAMtools. *Bioinformatics,* 25**,** 2078-9.

LIAO, Y., SMYTH, G. K. & SHI, W. 2013. The Subread aligner: fast, accurate and scalable read mapping by seed-and-vote. *Nucleic Acids Res,* 41**,** e108.

LIAO, Y., SMYTH, G. K. & SHI, W. 2014. featureCounts: an efficient general purpose program for assigning sequence reads to genomic features. *Bioinformatics,* 30**,** 923-30.

MENZIES, F. M., KHAN, A. H., HIGGINS, C. A., NELSON, S. M. & NIBBS, R. J. 2012. The chemokine receptor CCR2 is not required for successful initiation of labor in mice. *Biol Reprod,* 86**,** 118.

MERICO, D., ISSERLIN, R., STUEKER, O., EMILI, A. & BADER, G. D. 2010. Enrichment map: a network-based method for gene-set enrichment visualization and interpretation. *PLoS One,* 5**,** e13984.

OLLION, J., COCHENNEC, J., LOLL, F., ESCUDE, C. & BOUDIER, T. 2013. TANGO: a generic tool for high-throughput 3D image analysis for studying nuclear organization. *Bioinformatics,* 29**,** 1840-1.

OLMOS-ALONSO, A., SCHETTERS, S. T., SRI, S., ASKEW, K., MANCUSO, R., VARGAS-CABALLERO, M., HOLSCHER, C., PERRY, V. H. & GOMEZ-NICOLA, D. 2016. Pharmacological targeting of CSF1R inhibits microglial proliferation and prevents the progression of Alzheimer's-like pathology. *Brain,* 139**,** 891-907.

PICELLI, S., BJORKLUND, A. K., FARIDANI, O. R., SAGASSER, S., WINBERG, G. & SANDBERG, R. 2013. Smart-seq2 for sensitive full-length transcriptome profiling in single cells. *Nat Methods,* 10**,** 1096-8.

ROBINSON, M. D., MCCARTHY, D. J. & SMYTH, G. K. 2010. edgeR: a Bioconductor package for differential expression analysis of digital gene expression data. *Bioinformatics,* 26**,** 139-40.

SAFAIYAN, S., KANNAIYAN, N., SNAIDERO, N., BRIOSCHI, S., BIBER, K., YONA, S., EDINGER, A. L., JUNG, S., ROSSNER, M. J. & SIMONS, M. 2016. Age-related myelin degradation burdens the clearance function of microglia during aging. *Nat Neurosci,* 19**,** 995-8.

SASMONO, R. T., OCEANDY, D., POLLARD, J. W., TONG, W., PAVLI, P., WAINWRIGHT, B. J., OSTROWSKI, M. C., HIMES, S. R. & HUME, D. A. 2003. A macrophage colony-stimulating factor receptor-green fluorescent protein transgene is expressed throughout the mononuclear phagocyte system of the mouse. *Blood,* 101**,** 1155-1163.

SCHAMBACH, A., MUELLER, D., GALLA, M., VERSTEGEN, M. M., WAGEMAKER, G., LOEW, R., BAUM, C. & BOHNE, J. 2006. Overcoming promoter competition in packaging cells improves production of self-inactivating retroviral vectors. *Gene Ther,* 13**,** 1524-33.

VILLUNGER, A., MICHALAK, E. M., COULTAS, L., MULLAUER, F., BOCK, G., AUSSERLECHNER, M. J., ADAMS, J. M. & STRASSER, A. 2003. p53- and drug-induced apoptotic responses mediated by BH3-only proteins puma and noxa. *Science,* 302**,** 1036-8.

WEBER, K., BARTSCH, U., STOCKING, C. & FEHSE, B. 2008. A multicolor panel of novel lentiviral "gene ontology" (LeGO) vectors for functional gene analysis. *Mol Ther,* 16**,** 698-706.
