# Supplementary material for: Coupled Proliferation and Apoptosis Maintain the Rapid Turnover of Microglia in the Adult Brain
Source: Cell Rep. 2017 Jan 10;18(2):391–405. doi: 10.1016/j.celrep.2016.12.041 (PMC5263237; doi:10.1016/j.celrep.2016.12.041)
Supplement: Document S2. Article plus Supplemental Information [file mmc3.pdf]

Cell Reports

Article

Coupled Proliferation and Apoptosis Maintain the Rapid Turnover of Microglia in the Adult Brain

Graphical Abstract

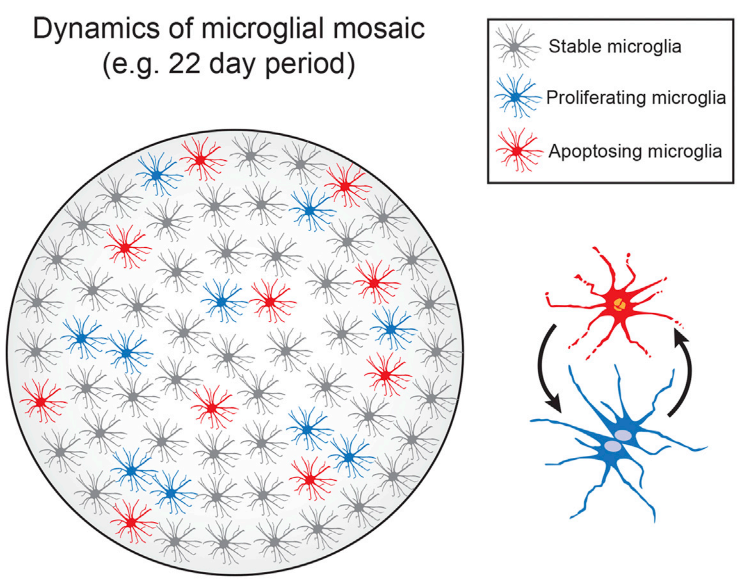

Highlights

- The microglial population is formed without the perinatal infiltration of monocytes
- The microglial density remains remarkably stable over a mouse or human lifetime
- In the mouse and human brain, microglia turn over several times during a lifetime
- Microglia self-renewal is maintained by coupled proliferation and apoptosis

Authors

Katharine Askew, Kaizhen Li, Adrian Olmos-Alonso, ..., Olga Garaschuk, V. Hugh Perry, Diego Gomez-Nicola

Correspondence

d.gomez-nicola@soton.ac.uk

In Brief

The mechanism or mechanisms underlying microglial homeostasis are unknown. Askew et al. show that microglia self-renewal is maintained by coupled proliferation and apoptosis, resulting in a stable microglia number over a mouse or human lifetime.

Askew et al., 2017, Cell Reports *18*, 391–405
January 10, 2017 © 2017 The Author(s).
http://dx.doi.org/10.1016/j.celrep.2016.12.041

Cell Reports
Article

OPEN ACCESS
CellPress

Coupled Proliferation and Apoptosis Maintain the Rapid Turnover of Microglia in the Adult Brain

Katharine Askew,[1] Kaizhen Li,[2] Adrian Olmos-Alonso,[1] Fernando Garcia-Moreno,[3] Yajie Liang,[2] Philippa Richardson,[1] Tom Tipton,[4] Mark A. Chapman,[1] Kristoffer Riecken,[5] Sol Beccari,[6] Amanda Sierra,[6] Zoltán Molnár,[3] Mark S. Cragg,[4] Olga Garaschuk,[2] V. Hugh Perry,[1] and Diego Gomez-Nicola[1,7,*]
[1]Biological Sciences, University of Southampton, Southampton General Hospital, Southampton SO16 6YD, UK
[2]Institute of Physiology II, University of Tübingen, 72074 Tübingen, Germany
[3]Department of Physiology, Anatomy and Genetics, University of Oxford, Oxford OX1 3PA, UK
[4]Antibody and Vaccine Group, Cancer Sciences Unit, University of Southampton, Southampton SO16 6YD, UK
[5]Research Department Cell and Gene Therapy, University Medical Center Hamburg-Eppendorf, 20246 Hamburg, Germany
[6]Achucarro Basque Center for Neuroscience, Ikerbasque Foundation, University of the Basque Country (UPV/EHU), 48940 Leioa, Bizkaia, Spain
[7]Lead Contact
*Correspondence: d.gomez-nicola@soton.ac.uk
http://dx.doi.org/10.1016/j.celrep.2016.12.041

SUMMARY

Microglia play key roles in brain development, homeostasis, and function, and it is widely assumed that the adult population is long lived and maintained by self-renewal. However, the precise temporal and spatial dynamics of the microglial population are unknown. We show in mice and humans that the turnover of microglia is remarkably fast, allowing the whole population to be renewed several times during a lifetime. The number of microglial cells remains steady from late postnatal stages until aging and is maintained by the spatial and temporal coupling of proliferation and apoptosis, as shown by pulse-chase studies, chronic in vivo imaging of microglia, and the use of mouse models of dysregulated apoptosis. Our results reveal that the microglial population is constantly and rapidly remodeled, expanding our understanding of its role in the maintenance of brain homeostasis.

INTRODUCTION

Microglial cells are the brain's resident innate immune cells, with proposed key roles in brain communication and the control of inflammation in brain disease (Gomez-Nicola and Perry, 2015), the developmental control of neurogenesis (Cunningham et al., 2013), wiring (Squarzoni et al., 2014) and synaptic pruning (Paolicelli et al., 2011), the monitoring of synaptic activity (Wake et al., 2009) and the regulation of adult neurogenesis (Sierra et al., 2010). Microglia account for 5%–12% of the total number of glial cells in the mouse brain (Lawson et al., 1990) and 0.5%–16.6% of the total number of cells in the human brain (Mittelbronn et al., 2001), depending on the region studied.

Microglia are derived from the yolk sac at embryonic day (E) 8.5 (Ginhoux et al., 2010), a lineage distinct from most other tissue-resident macrophages (Hoeffel et al., 2015), and they acquire their definitive local density soon after birth, after a wave of microglial proliferation at early postnatal stages (Nikodemova et al., 2015). However, it is unclear whether proliferation alone can account for the rapid increase in microglial numbers and suggests the possibility of additional recruitment and differentiation from blood-derived monocytes perinatally (Ginhoux et al., 2013), although the contribution of monocytes has not been observed in fate mapping studies (Hoeffel et al., 2015; Sheng et al., 2015).

In the adult, it has been suggested that the microglial population is long lived and maintained by self-renewal (Lawson et al., 1992), although the dynamics of the microglial population in the adult brain are largely unknown. However, evidence arising from manipulations of the numbers of resident microglial cells highlights that this population can be rapidly reconstituted by the proliferation of resident cells after genetic ablation by using the Cx3cr1CreER-based system (Bruttger et al., 2015), by pharmacological elimination (Elmore et al., 2014), or by infiltrating monocytes after the death of microglia induced using the CD11b-TK system (Varvel et al., 2012). Microglia are rarely replaced by bone marrow (BM)-derived progenitors in health or disease (Gomez-Nicola and Perry, 2015). These observations suggest that microglia resemble the behavior of other tissue-resident macrophage populations, like lung or BM macrophages, which are maintained by self-renewal in the steady state (Hashimoto et al., 2013). Although these studies suggest that microglia are a dynamic population and give some clues about the molecular determinants of the repopulation response, we do not know the rules governing the homeostatic maintenance of microglia during an organism's lifetime.

In this study, we show that the adult microglial population is formed without a contribution from circulating progenitors. We show that in the adult mouse and human brain, microglia display a high proliferation rate that accounts for several rounds of renewal of the whole population during the organism's lifetime. This proliferation is temporally and spatially coupled to intrinsic apoptosis, resulting in the maintenance of a relatively steady

CrossMark

This is an open access article under the CC BY license (http://creativecommons.org/licenses/by/4.0/).

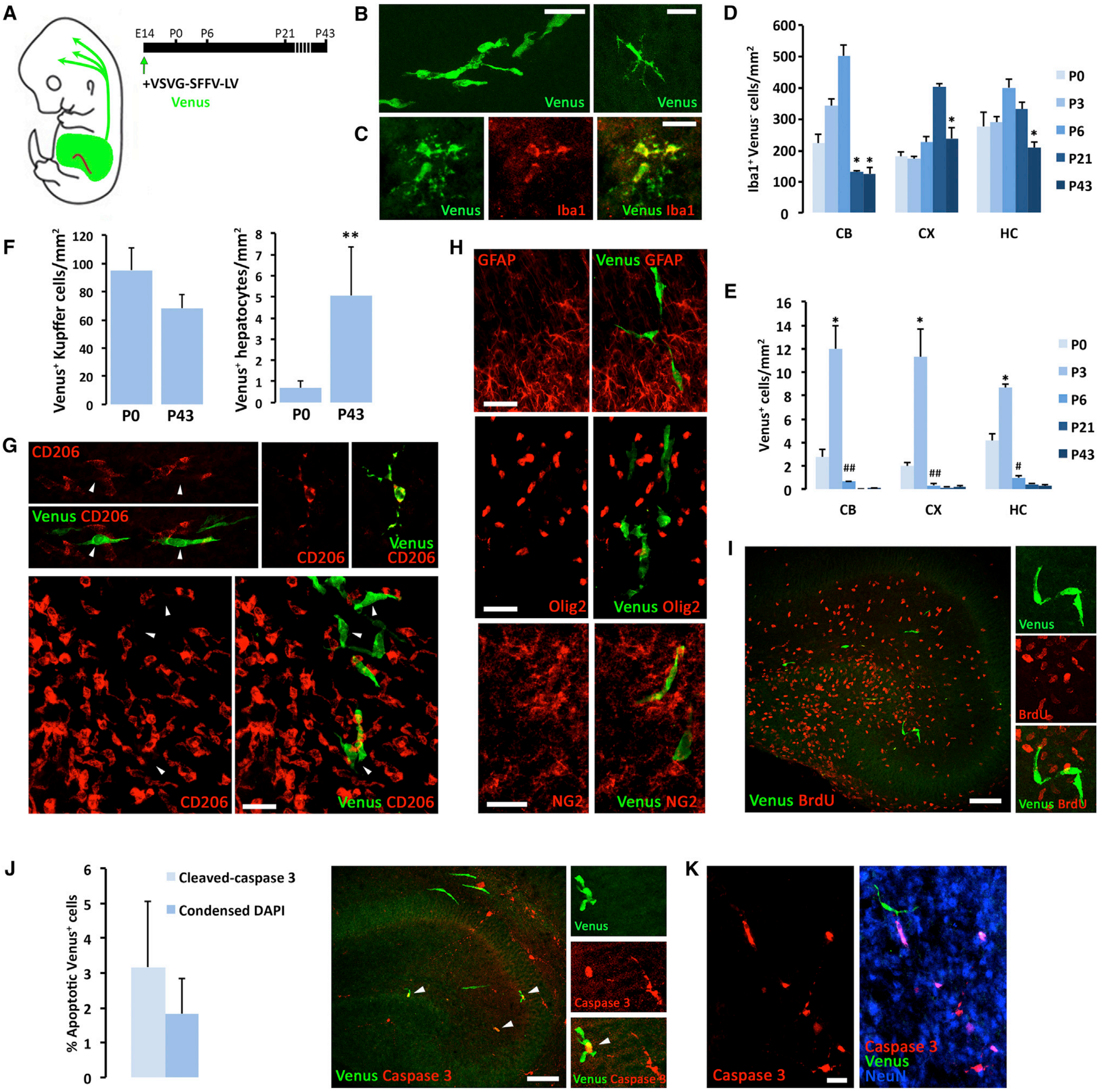

Figure 1. A Wave of Infiltrating Monocytes Invades the Brain at Early Postnatal Stages to Be Rapidly Depleted and Not Contributing to the Adult Microglial Population

(A) Experimental design, illustrating the tracing of late embryonic hematopoiesis by the intra-utero marking of liver progenitors with VSVG-SFFV lentiviral vectors (E14) and subsequent analysis of brain infiltration (P0–P43).

(B and C) Representative examples of Venus$^+$ (green) infiltrating cells at P3 (cerebellum), with migratory (bipolar, elongated) (B) or ramified (multiple radially orientated processes) (B, right) morphologies (C). Iba1 expression is shown in red in differentiated ramified cells.

(D and E) Time-course analysis of the number of resident microglia (Iba1$^+$Venus$^-$) and infiltrating monocytes (Venus$^+$) in the postnatal cerebellum (CB), cortex (CX), and hippocampus (HC). At all ages tested, Venus$^+$ cells (E) represent only a minority of all Iba1$^+$ cells (D).

(F–H) Phenotypic characterization of Venus$^+$ cells at P3 by confocal microscopy (G and H). Venus$^+$ cells (arrowheads) are CD206^{low} (red, F) and GFAP$^-$, Olig2$^-$, and NG2$^-$ (red, H).

(I) Representative example of the absence of cell proliferation (BrdU$^+$; red) in Venus$^-$ cells in the mouse postnatal hippocampus (P3).

(J) Quantification of the apoptosis of Venus$^+$ cells in the brain (cortex, hippocampus, and cerebellum) at P3, analyzed as expression of cleaved caspase-3 or condensation of chromatin (DAPI). A representative example of the expression of cleaved caspase-3 (red) in Venus$^+$ cells (green) is shown.

(legend continued on next page)

number of cells from early postnatal stages through to aging. Our results reveal highly dynamic but tightly regulated control of microglial cell numbers, expanding our understanding of the functions of microglia in the healthy and diseased brain.

RESULTS

A Perinatal Wave of Infiltrating Monocytes Does Not Contribute to the Adult Microglial Population

Although evidence supports the concept that the adult microglial population is generated from yolk sac emigrants (Ginhoux et al., 2010), followed by a wave of microglial proliferation (Nikodemova et al., 2015), it is unclear whether this alone accounts for the total increase in microglial cell numbers. Contrasting reports described the infiltration of blood-derived monocytes into the brain at perinatal stages (Alliot et al., 1999; Tambuyzer et al., 2009), although monocytes have been shown not to contribute to the adult microglial population (Hoeffel et al., 2015; Sheng et al., 2015). To address this question, we used an approach to label and track hematopoietic cells during embryonic development that is based on the in utero intra-liver delivery of lentiviral LeGO vectors driving the expression of the fluorescent protein Venus at E14, a stage when the liver is the main hematopoietic organ (Figure 1A). This method allows rapid, selective, and minimally invasive tracing of cells from the hematopoietic lineage and further analysis in target organs. Intra-liver tracing at E14 and subsequent analysis of the brain from postnatal day (P) 0 onward allowed the visualization of waves of infiltrating monocytes (Venus$^+$), acquiring migratory phenotypes (bipolar, elongated; 87.9% of all Venus$^+$ cells at P3) or ramified phenotypes (multiple radially orientated processes; 12.0% of all Venus$^+$ cells at P3) within the brain's parenchyma (Figures 1B and 1C). The visualization of the differentiation of Venus$^+$ cells into ramified Iba1-expressing morphologies supported the use of this tracking method for long-term purposes, because the expression of the Venus transgene was not affected by phenotypic changes with postnatal age (Figure 1C). Venus$^+$ cells were not found in the perivascular space of the blood vessels or in the meninges. This, together with the morphologies observed in Figure 1C, supports that Venus$^+$ cells infiltrate the parenchyma proper. The quantification of the total Venus$^+$ cells (ramified + migratory) (Figures 1B and 1C) showed the time course of infiltration (Figures 1D and 1E). This wave of infiltration was coincident in time with the expansion of the resident microglial population (Iba1$^+$Venus$^-$) followed by further refinement (Figure 1D), in accordance with previously reported data (Nikodemova et al., 2015). After a peak of infiltration at P3, the infiltrated monocytes were found to radically decrease in number until only rare cells survive in the adult brain (Figure 1E). These cells were not observed when vectors were delivered to the amniotic sac, supporting the specificity of the intra-liver approach. Intra-liver injections were comparable in different litters, as shown by a comparable degree of labeling of Kupffer cells and hepatocytes in the liver (Figure 1F), preserved over time. Venus$^+$ cells in the brain parenchyma were identified at P3 to be CD206^{low} when compared with choroid plexus or perivascular macrophages (Figure 1G) and GFAP$^-$Olig2$^-$NG2$^-$ (Figure 1H), supporting their monocytic lineage. Venus$^+$ cells were also defined as non-proliferative cells (Venus$^+$ bromodeoxyuridine [BrdU]$^-$) in the different regions analyzed (Figure 1I).

The analysis of cell death in the Venus$^+$ population revealed an apoptotic response from P3, as identified by the expression of cleaved caspase-3 or chromatin condensation (non-significant difference between activated caspase-3$^+$ cells versus cells with condensed nuclei stained with DAPI) (Figure 1J). We found that at P3, 1.83% (condensed DAPI) or 3.17% (act-caspase-3$^+$) of Venus$^+$ cells were apoptotic, with small variations across regions. Other cells, mostly neurons, were also found to be activated caspase-3$^+$ (Figure 1K) because of postnatal circuitry refinement. Given that the average time for an apoptotic cell to be removed from the brain is about 80 min (Sierra et al., 2013), and assuming that the clearance rates remain constant until adulthood, we estimated that the infiltrated monocytes could be removed from the brain parenchyma within approximately 42–72 hr. Although this is an estimation based on the mean rate of observed apoptosis, it helps explain the drop in Venus$^+$ cell numbers detected from P3 to P6 (Figure 1E).

In light of these data, we conclude that the adult microglial population is composed exclusively from yolk sac-derived cells, without the contribution of hematopoietic-derived monocytes infiltrating at perinatal stages.

The Number of Microglial Cells Remains Stable throughout Life in Mice and Humans

We next investigated the regional and temporal changes in the number of microglial cells in a select number of brain regions to understand their population dynamics. The density of murine microglial cells (Iba1$^+$) remained remarkably stable throughout, with little change from the young (4–6 months) to the aged (18–24 months) brains in all areas analyzed except the thalamus, where an increased number was found with aging (Figure 2A). Microglial cells were denser in gray matter-enriched versus white matter-enriched areas, as previously shown (Lawson et al., 1990). To better understand whether the maintenance of microglial numbers was achieved by local self-renewal or by a contribution from circulating monocytes, we compared the microglial density in young versus aged CCR2$^{-/-}$ and wild-type (WT) mice. CCR2$^{-/-}$ monocytes have deficient egress from the bone marrow, leading to fewer circulating monocytes (Serbina and Pamer, 2006) and making them a valuable model in which to study the role of recruited monocytes (Gomez-Nicola and Perry, 2015). The contribution of patrolling monocytes (CX3CR1$^+$/CCR2$^-$) was not studied by our approach and cannot be excluded, although these cells have been shown to infiltrate the CNS only under pathological conditions (Shechter et al., 2013). Neither young nor aged CCR2$^{-/-}$ mice had a different

(K) Expression of cleaved caspase-3 in NeuN$^+$ neurons at P3. Venus$^+$ cells are shown in green.

Scale bars are 20 μm in (B), (C), (G), (H), and (K) and 100 μm in (I) and (J). Data shown in (D), (E), (F), and (J) are represented as mean ± SEM (n = 6). Statistical differences: (D) CB *$p < 0.05$ versus P6, CX *$p < 0.05$ versus P21, HC *$p < 0.05$ versus P6. (E) *$p < 0.05$ versus P0, #$p < 0.05$ versus P3, ##$p < 0.01$ versus P3. (F) **$p < 0.01$. Data were analyzed with a two-way ANOVA and a post hoc Tukey test (D and E) or a t test (F).

OPEN
ACCESS
CellPress

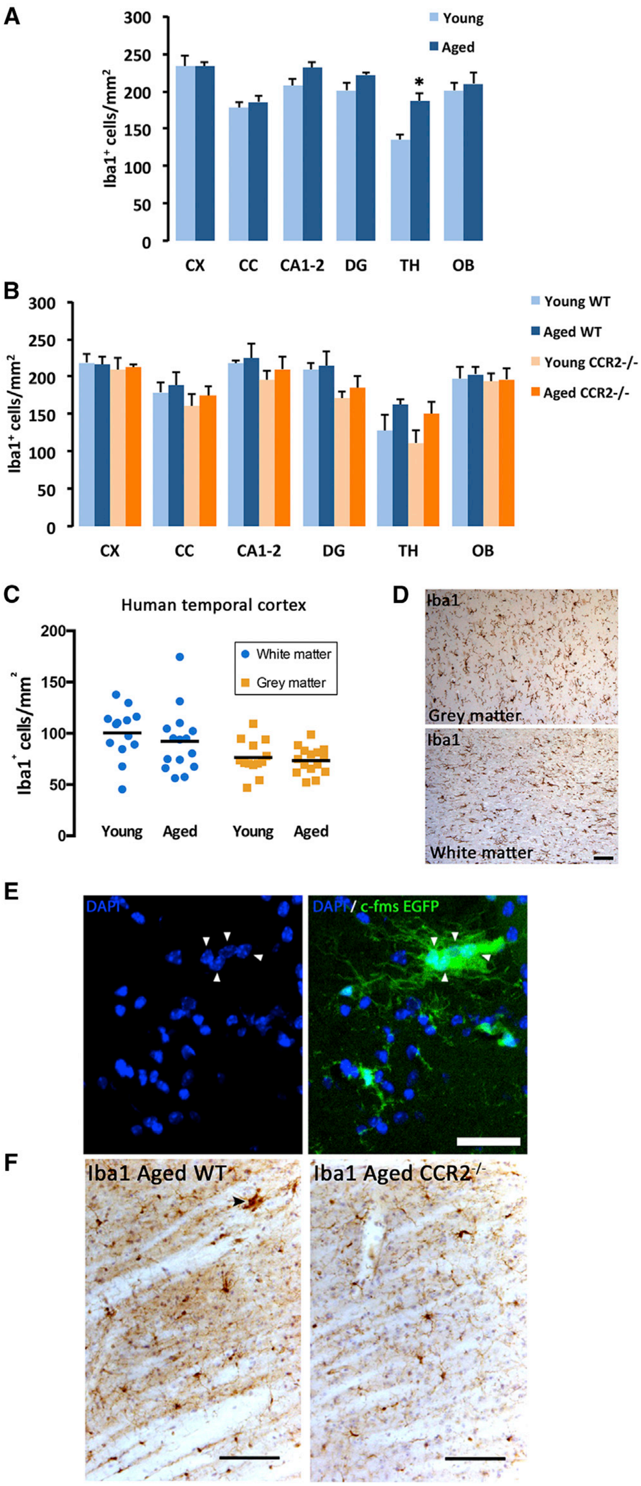

Figure 2. The Density of Microglial Cells Remains Steady Through the Lifetime, without a Significant Contribution of Circulating Monocytes

(A) Quantification of microglial density (Iba1$^+$ cells) across brain regions (CX, cortex; CC, corpus callosum; CA1–2, hippocampal CA1–CA2; DG, dentate gyrus; TH, thalamus; OB, olfactory bulb) in young (4–6 months) and aged (18–24 months) mice.

(B) Quantification of microglial density (Iba1$^+$ cells) across brain regions (A) in young (4–6 months) and aged (18–24 months) wild-type (WT) or CCR2$^{-/-}$ mice.

(C) Quantification of microglial density (Iba1$^+$ cells) in the white and gray matter of the human temporal cortex in young or aged individuals.

(D) Representative images of Iba1 staining in human temporal cortex.

(E) Representative example of a multinucleated microglial aggregate (c-fms EGFP) in aging mice.

(F) Representative examples of multinucleated microglial aggregates in aging WT mice, absent from CCR2$^{-/-}$ mice.

Scale bars are 50 μm in (D) and (E) and 50 μm in (F). Data shown are represented as mean ± SEM. n = 7 (A and B), n = 15 (C). Statistical differences: $^*p < 0.05$. Data were analyzed with a two-way ANOVA and a post hoc Tukey test (A–C).

number of microglial cells when compared to WT mice (Figure 2B), suggesting that circulating monocytes do not contribute significantly to the microglial population during a healthy lifetime.

A similar picture was observed when we compared young (20–35 years old) versus aged (58–76 years old) human cases. The density of microglia in the gray or white matter of the temporal cortex was found to be unchanged with aging (Figure 2C). Microglial cell density was greater in the white matter than in the gray matter (Figures 2C and 2D), in agreement with previous findings (Mittelbronn et al., 2001). This pattern of distribution opposes that previously found in rodents (Lawson et al., 1990), indicating species-specific regional differences in the microglial population.

Altogether, these data demonstrate that the density of microglial cells is remarkably stable in young and aged brains from both mice and humans.

The analysis of microglia in aged mice led to the identification of small numbers of multinucleated microglial aggregates (Figure 2E), previously described in the aged rat brain (Perry et al., 1993). Multinucleated microglial aggregates were more frequent in the aged thalamus and cerebellum (Figure S1A) and expressed major histocompatibility complex class II (MHC class II) (Figure S1B), as well as CD45 (data not shown). Using confocal microscopy, we could identify aggregates containing up to ten nuclei within the same cytoplasmic syncytium (Figure S1C). To better understand whether failed cytokinesis after increased proliferation was the origin of these aggregates, we performed analysis of proliferation after repeated BrdU incorporation using confocal microscopy (Figure S1D). The incorporation of BrdU was minimal in these aggregates (0.48% were BrdU$^+$ and showed only two cells per aggregate) (Figure S1D), ruling out the hypothesis of failed cytokinesis. Our next hypothesis was that these aggregates could have a peripheral origin, which was confirmed after observing that aged CCR2$^{-/-}$ mice were devoid of multinucleated microglial aggregates (Figure 2F).

Microglia Have a High Proliferation Rate in the Mouse and Human Brain

Although it is often assumed that the microglial population is maintained by a slow turnover of long-lived resident cells, little formal evidence exists (reviewed in Gomez-Nicola and Perry, 2015). Earlier work from Lawson et al. (1992), using H^3 thymidine combined with immunohistochemistry for F4/80, demonstrated

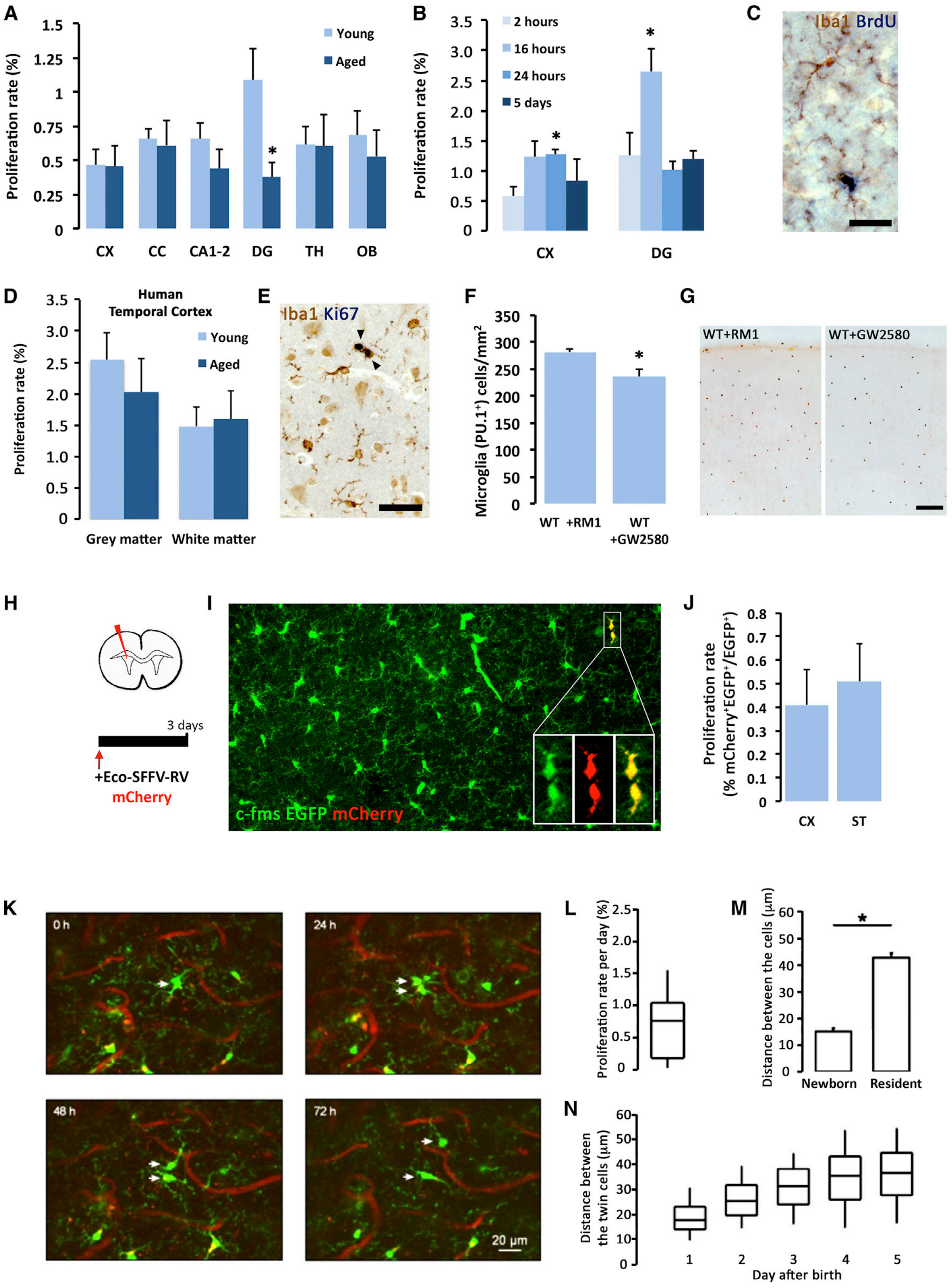

Figure 3. Proliferation of Microglia in the Adult Mouse and Human Brain

(A) Analysis of the proliferation (proliferation rate, %) of microglia across brain regions (CX, cortex; CC, corpus callosum; CA1–2, hippocampal CA1–CA2; DG, dentate gyrus; TH, thalamus; OB, olfactory bulb) in young (4–6 months) and aged (18–24 months) mice.

(B) Time-course analysis of microglial proliferation (proliferation rate, %) and death in the mouse cortex (CX) and dentate gyrus (DG).

(C) Representative example of a proliferating microglial cell (Iba1$^+$, brown), incorporating BrdU (blue).

(legend continued on next page)

that microglia proliferate in the healthy brain but do so more slowly than other tissue macrophages: 0.05% of the microglia were proliferating at a given time, 20 times less than the lowest labeling index for any other resident macrophage populations studied (Lawson et al., 1992). We set out to analyze the proliferation of resident microglia by using more sensitive techniques (BrdU incorporation detected in $Iba1^+$ cells by double immunohistochemistry of diaminobenzidine [DAB] and alkaline phosphatase [AP]) (Olmos-Alonso et al., 2016). We found that microglial proliferation rates in the adult brain were approximately ten times higher (Figures 3A–3C) than those previously reported by Lawson et al. (1992). On average, 0.69% of the total microglial cells were proliferating ($Iba1^+BrdU^+$) after a single pulse of BrdU. This rate was particularly high in the dentate gyrus (DG), the only area where we also found that aging had an impact on the proliferation rate (Figures 3A–3C). We ensured that these rates were not underestimated by the dose of BrdU, because we had performed a dose-response analysis of microglial proliferation confirming that the BrdU dose (7.5 mg/mL) used was optimal (Figures S2A and S2B). We next performed a time-course analysis of proliferation and division after a single pulse of BrdU in microglial cells (Figure 3B). In the cortex, we could detect the duplication of the proliferating population from 16 hr after the BrdU pulse, indicating successful cell-cycle exit and cell division (Figure 3B). Return to the baseline number of $Iba1^+BrdU^+$ cells was observed from 24 hr. Considering that the S phase of mammalian cells comprises ~50% of the duration of the cell cycle, with G_2/M only taking a few hours (Cameron and Greulich, 1963), this allows an estimate of a cell-cycle length (*Tc*) of 32 hr. This would be in agreement with reported cell-cycle lengths of macrophages, which vary depending on the differentiation stage from 20 to 40 hr (Kueh et al., 2013). If the S phase spans ~50% of the cell-cycle length, our data from BrdU labeling would only detect half of the dividing population. This indicates that ~1.38% of the population will be proliferating at a given time (*F* = fraction of cells in a cell cycle). If we use these rates to calculate the time needed for the entire rodent microglial population to renew (*X*), with the equation

$$X = \frac{100xTc}{F}$$

we can estimate that the population renews once every 2,318 hr (~96 days), allowing as many as six cycles of complete renewal during an animal's lifetime (average 21 months). However, these calculations are based on estimations of *Tc* and require further specific study.

The proliferative cycle was quicker in the DG, where the initial duplication returned to baseline before 24 hr (Figure 3B). In addition to revealing the higher proliferative activity of microglia in the DG, these data strongly suggest that microglial death must be tightly temporally and spatially coupled to proliferation to maintain the stable density of microglial cells, as discussed later.

Higher figures were observed when analyzing the proliferation of human microglia (on average, 2% of the microglial population proliferating at a given time), according to double staining of Iba1 and Ki67 (Figures 3D and 3E). This rate is 2.9 times higher than that observed for mice described earlier (0.69%). However, Ki67 expression is not directly comparable to BrdU incorporation. This difference might be explained by how Ki67 would label not only the S phase but also other cell-cycle phases except G_0. This means the labeling of Ki67 is approximately two times higher than that of BrdU (Kee et al., 2002), which only labels the S phase, comprising ~50% of the duration of the cell cycle (Cameron and Greulich, 1963). If cell-cycle length remains constant in mammals (32 hr, as noted earlier), this would allow an estimation of hundreds of cycles of complete renewal during a lifetime (average 80 years).

To further explore age-related changes in microglial proliferation, we studied the expression of genes related to the colony stimulating factor 1 receptor (CSF1R)-driven proliferative response (Gómez-Nicola et al., 2013). We found a significant reduction in the expression of *PU.1* and *IRF8* in aging brains and a non-significant trend toward a reduction in relevant genes like *CSF1*, *CSF1R*, *C/EBPα*, *CD34*, or *RUNX1* (Figure S3). To further address the significance of the CSF1R pathway in controlling microglial turnover, we administered young mice a diet containing GW2580, a specific CSF1R inhibitor previously shown to cause blockade of microglial proliferation, but not microglia survival (Gómez-Nicola et al., 2013; Uitdehaag et al., 2011; De Lucia et al., 2016; Olmos-Alonso et al., 2016), in contrast to the microglia-depleting effects caused by the CSF1R inhibitor PLX3397 (Elmore et al., 2014). Treatment with GW2580 for 3 months decreased the total number of microglial cells ($PU.1^+$) by 17% (Figures 3F and 3G), supporting the relevance of the CSF1R pathway in controlling the homeostatic maintenance of microglial turnover.

(D and E) Analysis of the proliferation (proliferation rate, %) of microglia in the human white or gray matter of the temporal cortex, analyzed as expression of Ki67 (blue) in $Iba1^+$ cells (brown), as shown in the representative example (E).

(H–J) Analysis of microglial proliferation by tracing c-fms EGFP mice with Eco-SFFV mCherry γ-retroviral vectors (Eco-SFFV-RV mCherry). (H) Experimental scheme. (I) Representative image of the tracing of proliferating microglia by Eco-SFFV-RV (mCherry, red) in the cortex of c-fms EGFP mice (green). (J) Analysis of the proliferation (proliferation rate, % $mCherry^+EGFP^+$/total $EGFP^+$) of microglia (CX, cortex; ST, striatum) in c-fms EGFP mice.(K–N) Analysis of microglial proliferation by two-photon imaging of $CX_3CR1^{GFP/+}$ mice. (K) Maximal intensity projection (MIP) images of the same field of view (142–153 μm depth, 1 μm step) in a $CX_3CR1^{GFP/+}$ mouse taken at different time points as indicated (see timestamps, relative time). Arrows point to a proliferating microglial cell and its progeny. (L) Proliferation rate of microglia (median ± interquartile range [IQR]; n = 669 cells, 9 fields of view [FOVs], and 4 mice). (M) Mean distance between the centers of two neighboring cells for resident cells and for newborn cells during the first 24 hr of their life (mean ± SEM; n = 62 cells, 9 FOVs, and 4 mice). (N) Distance between the twin microglial cells as a function of their age (median ± IQR; n = 31 pairs of twin cells, 8 FOVs, and 4 mice).

Scale bars are 20 μm in (A) and (C), 50 μm in (E), and 100 μm in (G). Data shown are represented as mean ± SEM. n = 8 (A and B), n = 15 (D), n = 6 (F), n = 5 (J). Statistical differences: (A–J) *$p < 0.05$; (M) *$p < 0.001$, Student's t test. Data were analyzed with a two-way ANOVA and a post hoc Tukey test (A and B) or a Student's t test (F and J).

To provide an independent method to validate our analysis of microglial proliferation in mice, we took advantage of the ability of γ-retroviral vectors to selectively transduce proliferating glial cells (Gomez-Nicola et al., 2014). We delivered an Eco-SFFV γ-retroviral vector driving the expression of mCherry to the lateral ventricle of CSF1R promotor (c-fms) EGFP mice, allowing diffusion to adjacent areas (cortex and striatum) due to the initially injected volume (5 μL) (Figure 3H). We analyzed the incorporation of Eco-SFFV-RV (retroviral vector) mCherry 3 days after injection to allow the expression of detectable levels of mCherry (Gomez-Nicola et al., 2014) and the potential visualization of pairs of cells before postdivision microglial death (Figure 3B). We found a limited number of microglial cells (EGFP$^+$) expressing mCherry, presenting as typical microglial duplets (Figure 3I). The quantification of proliferating microglial cells (mCherry$^+$EGFP$^+$) offered a proliferation rate (Figure 3J) similar to that previously described by analyzing the incorporation of BrdU in Iba1 cells (Figure 3A), validating our previous findings.

For direct visualization of microglial turnover, we used chronic live imaging of the olfactory bulb microglia in $CX_3CR1^{GFP/+}$ mice, coupled to repeated blood vessel imaging (Figure S4A) (Kovalchuk et al., 2015). To control for potential interference of the implantation of the chronic window on the microglial behavior, mice were analyzed 3–4 weeks after surgery to allow initial inflammation to resolve. After this, imaged microglia were typical highly branched, CD11b^{low} and CD68$^-$ (Figures S4B and S4C), and therefore considered surveillant microglia. Repeated live imaging of microglia allowed the identification of cell division (duplication) (Figure 3K) or death (disappearance) (see Figure 6A later) and defined the proliferation rate of microglia at 0.79% per day (Figure 3L), similar to the rate we found with Iba1/BrdU staining (Figure 3A). During the first 24 hr after division, paired microglia were found at a significantly closer distance than resident non-dividing microglia (Figure 3M), suggesting that these cells were generated from the same proliferating cell. During the following days, the cells migrated away from each other and reached cell-to-cell dispersion similar to the rest of the microglial population within 3–4 days (Figure 3N). These data confirm the high rates of microglial proliferation detected by Iba1/BrdU staining and suggest that the territories occupied by microglia change upon cell division, probably affecting the performance of local homeostatic functions.

Thus, using three independent lines of evidence, our data show that microglia proliferate in the adult mouse and human brain at a high rate, allowing several cycles of renewal of the whole population during the organism's lifetime.

Microglial Turnover Is Not Maintained by Nestin$^+$ Precursors

In light of findings suggesting that Nestin$^+$ microglial precursors may be involved in the repopulation response after pharmacological microglial ablation (Elmore et al., 2014) or transgenic microglial ablation (Bruttger et al., 2015), we aimed to study whether microglial proliferation in the steady state was maintained by a subpopulation of stem cell-like microglia. We analyzed Nestin-GFP mice as an optimal reporter mouse for the expression of nestin (Mignone et al., 2004), and although we showed the previously reported expression in pericytes (Figures 4A–4C), neural stem cells (Figure 4B), and oligodendrocyte precursor cells (Figures 4A and 4C) (Mignone et al., 2004), we did not find evidence of nestin expression in microglia (Iba1$^+$) (Figures 4A–4C). We specifically studied the expression of nestin in proliferating microglia (Iba1$^+$BrdU$^+$; 24 hr postinjection) and found no evidence of nestin$^+$ microglia (Figure 4D). We therefore conclude that microglial proliferation is not maintained by Nestin$^+$ microglial precursors in the steady state.

Microglial Proliferation and Apoptosis Are Temporally and Spatially Coupled to Maintain Microglial Homeostasis

The time-course analysis of microglial proliferation (Figure 3B) suggested that microglial cell death plays a key role in maintaining the stable number of microglia over time. Given the difficulties of analyzing microglial apoptosis by traditional methods (Sierra et al., 2013), we set out to address this point by using live imaging of microglia in $CX_3CR1^{GFP/+}$ mice. Under these conditions, microglial death is defined as a disappearance of a cell within the network of relatively immobile neighboring cells (Figure 5A), with the blood vessel pattern providing additional landmarks. The death rate of resident microglia was found to be 1.23% per day, while the death rate for newborn (recently divided) microglia was 2.40% (Figure 5B). For newborn microglial cells, the death rate was highest during the first 5 days after division (5.0% $\pm$ 3.5%), significantly higher than the death rate in the resident adult cell population.

To further study the relevance of microglial apoptosis in the maintenance of the population, we studied the numbers of microglia in three mouse models defective in intrinsic apoptosis (PUMA$^{-/-}$, BIM$^{-/-}$, and Vav-Bcl2). While the first two have ubiquitous deletion of the pro-apoptotic Bcl-2 homology domain 3 (BH3)-only molecules PUMA or BIM, the Vav-Bcl2 mice have the anti-apoptotic molecule Bcl2 overexpressed only in cells of the myeloid lineage. When compared to WT mice, both BIM$^{-/-}$ and Vav-Bcl2 mice were found to have a significant increase in the number of microglial cells (Figure 5C). PUMA$^{-/-}$ showed no difference, or even a reduction, in the number of microglia (Figure 5C), in agreement with previous findings in the eye (Zhang et al., 2012) and suggesting that microglial apoptosis is PUMA independent. Because Vav-Bcl2 mice provide a robust block in intrinsic apoptosis only in lymphoid and myeloid lineage cells (Egle et al., 2004), which in the brain is restricted to microglia, we decided to focus on the study of this model (Figure 5D). A time course of postnatal development of the microglial population in Vav-Bcl2 mice showed that the increase in the number of microglial cells is reached early in life (P44) and remains stable until middle age (before the onset of other health defects) (Figures 5E and 5F) (Egle et al., 2004). This stabilization of increased density, caused by deficient microglial apoptosis, is perhaps explained by the inability of the parenchyma to accommodate more cells, suggesting contact inhibition mechanisms are in place.

To understand the impact of apoptosis blockade on microglial phenotype, we isolated microglia by flow cytometry analysis and sorting (FACS) and analyzed their transcriptomic profile by RNA sequencing (RNA-seq) (Figure 6). Flow cytometry analysis showed a significant increase in the population of

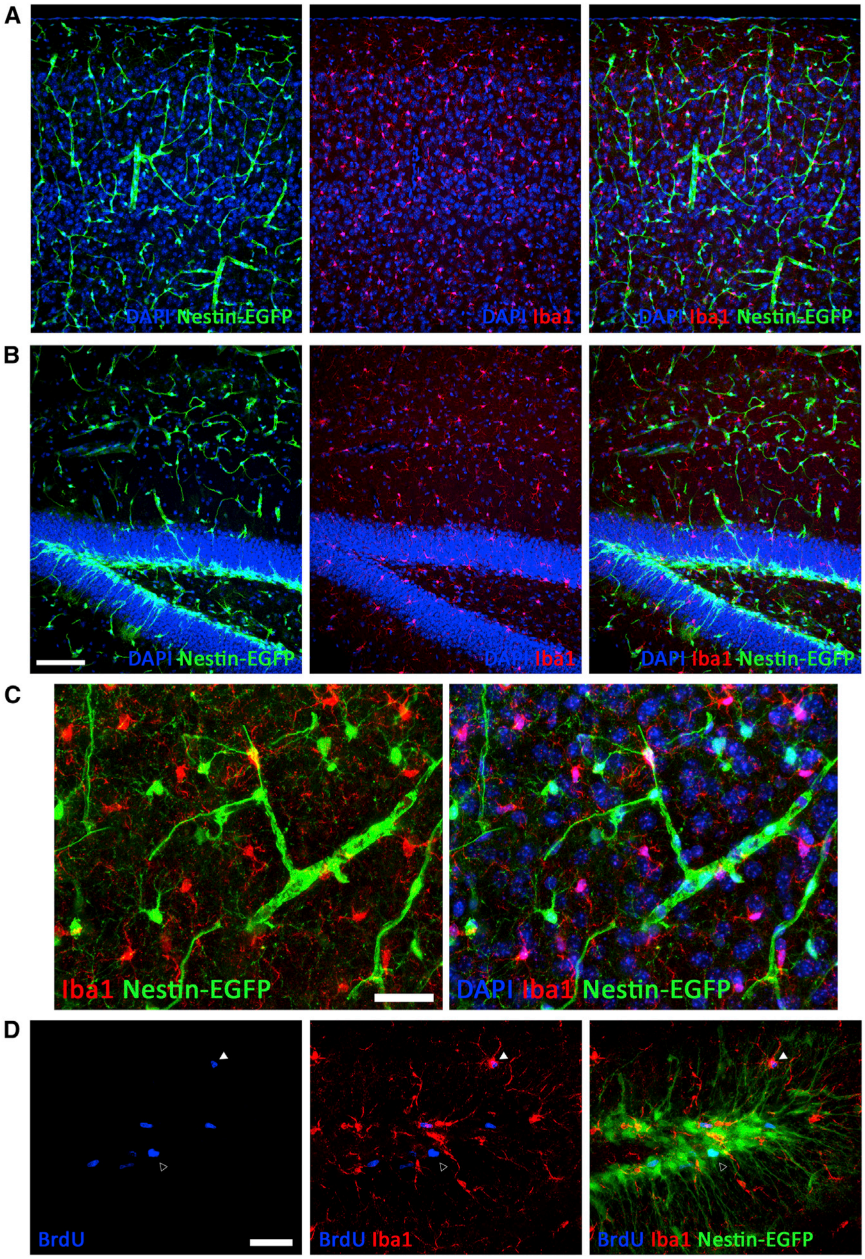

Figure 4. The Homeostatic Turnover of Microglia Is Not Maintained by Nestin$^+$ Precursors

(A–C) Immunofluorescent detection and confocal analysis of Iba1$^+$ microglia (red) in nestin-EGFP (green) mice in the cortex (A and C) or hippocampal dentate gyrus (B).

(D) Triple immunofluorescence for BrdU (blue), Iba1$^+$ (microglia, red), and nestin-EGFP (green) in the dentate gyrus. An open arrowhead indicates a BrdU$^+$Iba1$^-$Nestin$^+$ cell, while a white arrowhead indicates a BrdU$^+$Iba1$^+$Nestin$^-$ cell.

Scale bars are 50 μm in (A) and (B) and 20 μm in (C) and (D). n = 5.

CD11b$^+$CD45^{high} cells in Vav-Bcl2 mice when compared to WT littermates (Figure 6A), identifying this subpopulation of microglia as the biggest contributor to increased numbers observed previously. Isolation of CD11b$^+$CD45^{low} and CD11b$^+$CD45^{high} subpopulations from WT and Vav-Bcl2 followed by RNA-seq profiling rendered a total of 137 genes statistically ($p < 0.01$; fold change > 10) upregulated in Vav-Bcl2 versus WT and 259 genes statistically downregulated in Vav-Bcl2 versus WT microglia (Figure 6B; Table S1). Gene Ontology analysis revealed that differentially expressed genes were particularly associated with gene ontology (GO) Slim terms involved in metabolic processes and biogenesis (Figure 6C). Clustering of GO terms (based on genes shared between each GO category) revealed GO processes previously identified to be upregulated in microglia (Grabert et al., 2016), including immune response and macromolecule biosynthesis (Figure 6D). Vav-Bcl2 mice also showed a significant alteration of genes clustered under the processes of the cell cycle, proliferation, and death, confirming the expected effects of Bcl-2 upregulation (Figure 6C). Vav-Bcl2 microglia had significant repression (>200-fold) of the pro-apoptotic gene Bad and significant upregulation (17-fold) of the anti-apoptotic gene Api5 (Table S1). In addition, Vav-Bcl2 microglia showed significant repression of cell-cycle-promoting genes like Mad2l1, Mdm2, Cdca3, Cdk1, Cdc20, and Cdc20b (all > 25-fold downregulated) (Table S1). These changes confirm the anti-apoptotic effects of Bcl-2 overexpression in microglia but also suggest impaired cell-cycle regulation as an associated effect. A Venn diagram showing the relations between the gene sets of CD45^{low} and CD45^{high} identifies the CD11b$^+$CD45^{low} subpopulation as the major contributor to the transcriptional variability observed between WT and Vav-Bcl2 microglia (Figure 6E).

Although Vav-Bcl2 mice had significant alteration in the number and phenotype of microglia through most of their adult life, they did not display gross deficiencies in the astrocyte populations (Figure S5A) or the neuronal populations (Figure S5B) and only showed minor differences in age-dependent changes in synaptic density (Figure S5C). Vav-Bcl2 mice showed no differences in behavioral performance when compared to WT mice (Figures S6A and S6B).

We next analyzed the temporal and spatial relationship between death and proliferation events in vivo. We observed rapid reorganization of the microglial landscape (Figure 7A). This is exemplified by the representation of the history of microglia during the 22-day-long imaging period in a sample field of view, with

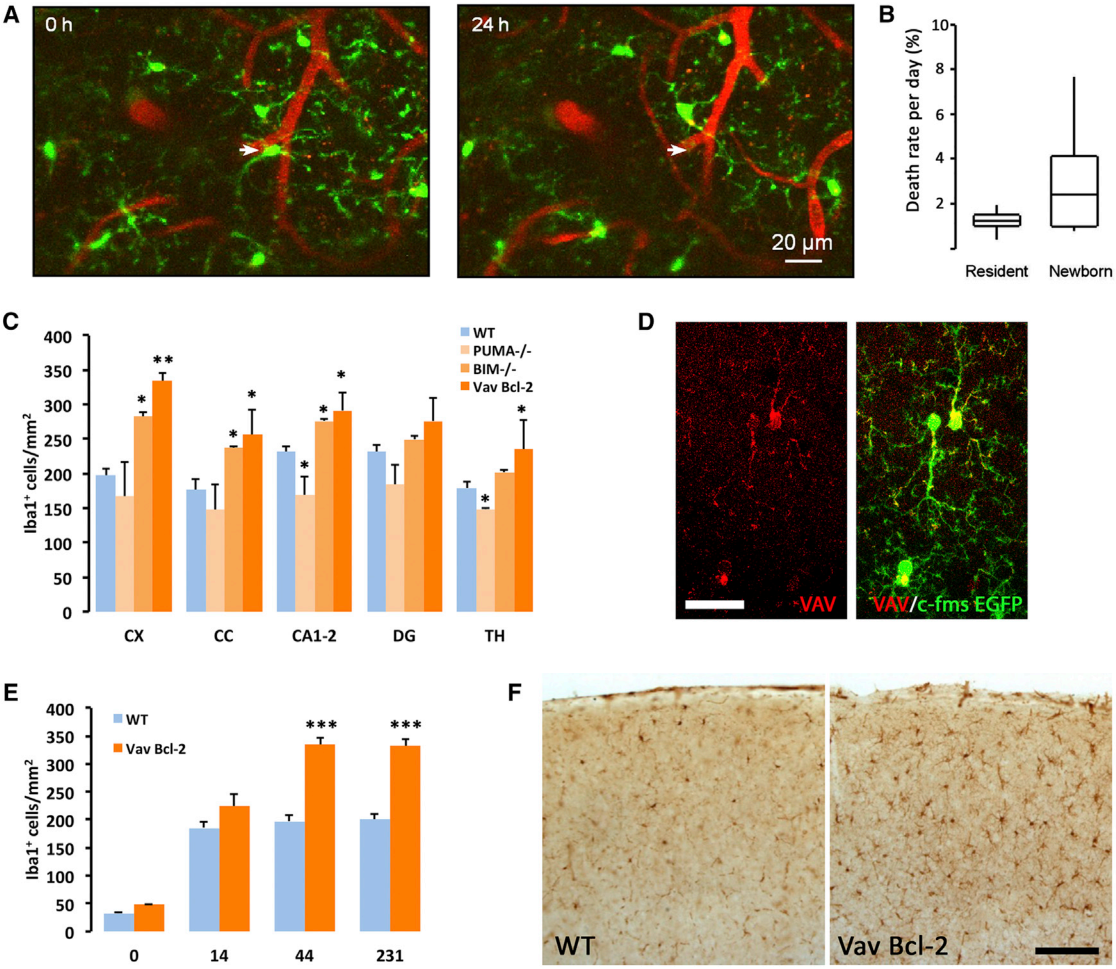

Figure 5. The Turnover of Microglia Is Balanced by Apoptosis

(A) Maximal intensity projection (MIP) images of the same field of view (88–106 μm depth, 2 μm step) in a $CX_3CR1^{GFP/+}$ mouse. Arrows point to a disappearing (i.e., dying) microglial cell.

(B) Death rate of microglia (median ± IQR; n = 669 cells, 9 FOVs, and 4 mice).

(C) Microglial density across regions (CX, cortex; CC, corpus callosum; CA1–2, hippocampal CA1–CA2; DG, dentate gyrus; TH, thalamus) in wild-type (WT), $PUMA^{-/-}$, $BIM^{-/-}$, and Vav-Bcl2 mice.

(D) Expression of Vav (red) in microglia (c-fms EGFP, green), analyzed by confocal microscopy.

(E) Time-course analysis of postnatal (P0–P231) microglial density in wild-type (WT) and Vav-Bcl2 mice.

(F) Representative example of microglial cells ($Iba1^+$) in the cortex of WT and Vav-Bcl2 mice.

Scale bars are 20 μm in (A) and (D) and 100 μm in (F). Data shown in (C) and (E) are represented as mean ± SEM. n = 4 WT mice, 3 $PUMA^{-/-}$ mice, 4 $BIM^{-/-}$ mice, and 7 Vav-Bcl2 mice (C); n = 4 (E). Statistical differences: *p < 0.05, **p < 0.01, ***p < 0.001. Data were analyzed with a two-way ANOVA and a post hoc Tukey test (C and E).

stable cells shown in gray, cells going to die shown in red, and cells going to divide shown in blue (Figure 7B). Microglial proliferation and apoptosis were spatially and temporally coupled, because many more cells proliferated in the vicinity (≤200 μm) of a dying cell immediately after its death (Figure 7C). Some of the proliferating cells were immediately adjacent to the dying cells, whereas others were located more distantly (Figures 7B and 7D). The median distance between the dying and the nearest proliferating cell was 72.95 ± 43.13 μm (n = 19 cell pairs), almost double the distance between the dying and the nearest resident cell (37.94 ± 11.74 μm, n = 31 cell pairs) (Figures 7D and 7E). Overall, proliferating cells were found to be the second-closest neighbors to dying cells.

In summary, our data indicate that the microglial population undergoes constant and rapid remodeling based on temporal and spatial coupling of proliferation and apoptosis, providing a mechanism for the homeostasis of the population through life. This constant renewal causes not only the individual cellular players to change but also their spatial layout to be rapidly modified.

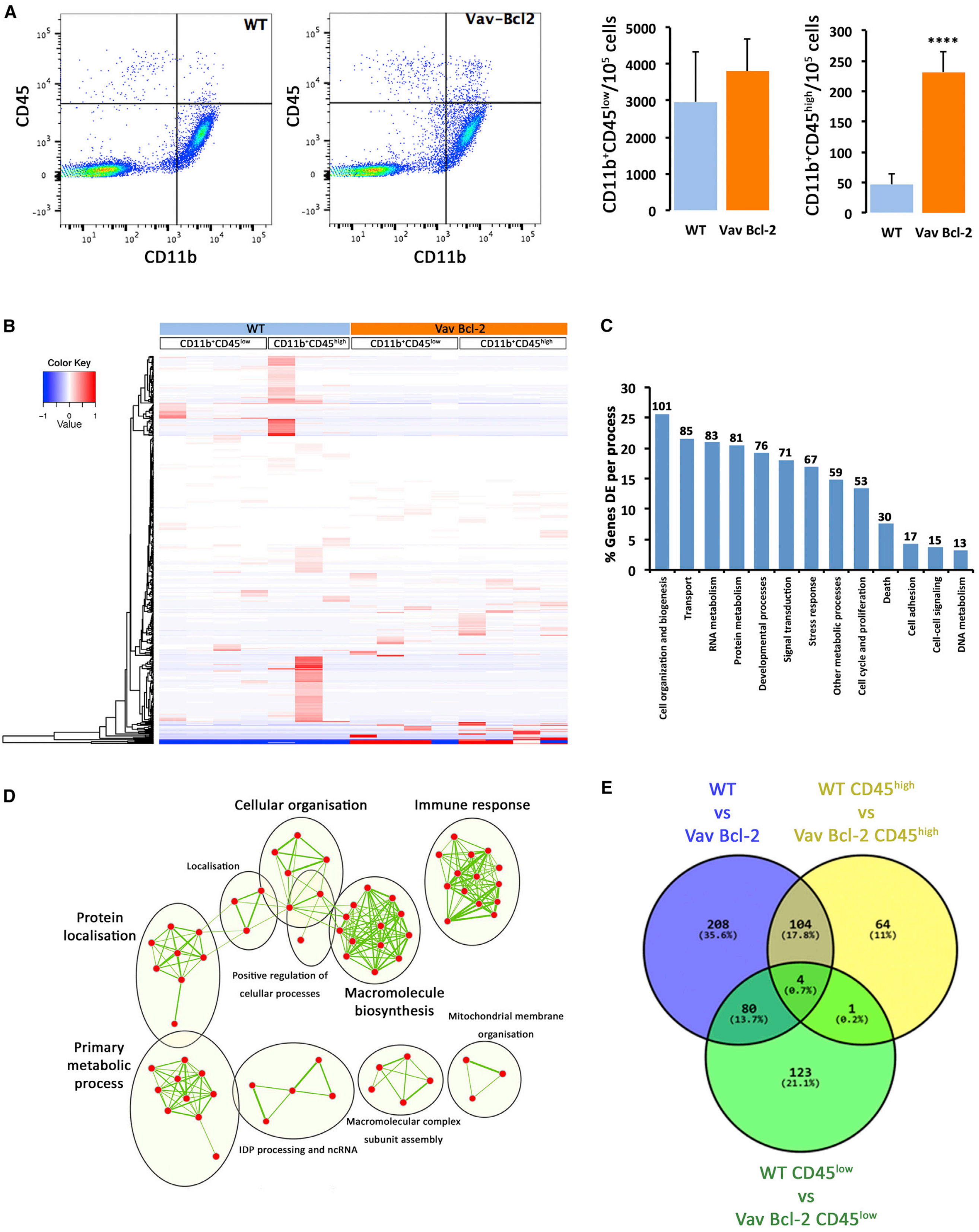

(legend on next page)

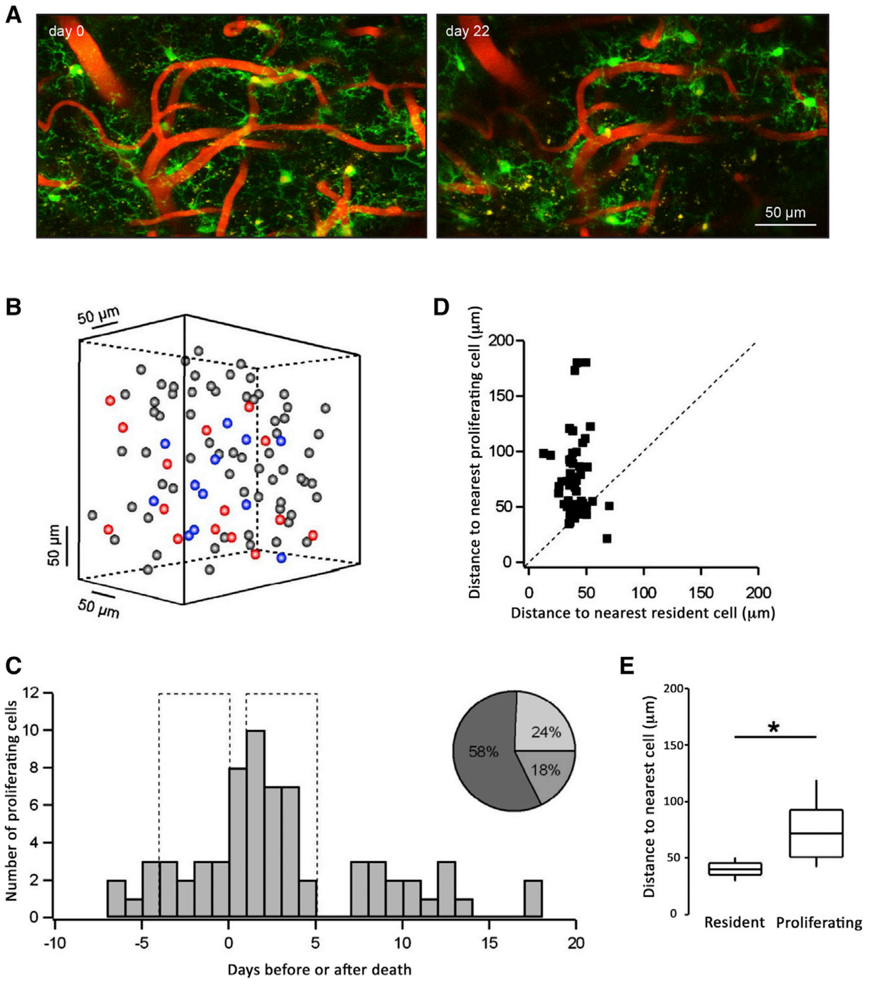

Figure 7. Temporal and Spatial Coupling of Microglial Proliferation and Death

(A) MIP images of a sample field of view (50–80 µm depth, 1 µm step) in a $CX_3CR1^{GFP/+}$ mouse taken at the beginning (left, day 0) and at the end (right, day 22) of the imaging period. Bone growth occurred in the lower right corner of the latter image.

(B) 3D matrix illustrating the history of cells in the sample field of view (317 × 317 × 160 µm) during the 22-day-long imaging period. Stable cells are shown in gray, cells that are going to die are shown in red, and cells that are going to divide are shown in blue. This FOV includes the cells shown in (A).

(C) Temporal relationship between death and proliferation events (n = 68 cells, 9 FOVs, and 4 mice). The time when a cell dies is set as day 0 (reference point), and the relative time when proliferation occurs in its vicinity (≤200 µm) is calculated. The pie chart illustrates the fractions of cells proliferating in the vicinity of a dying cell 4 days before (light gray), during (gray), or 4 days after (dark gray) the death of the reference cell.

(D) Spatial relationship between a dead cell and the nearest proliferating or resident cell (n = 53 dead cells, 9 FOVs, and 4 mice).

(E) Summary of the data shown in (D) (median ± IQR; n = 53 cells, 9 FOVs, and 4 mice).

Statistical differences: *p < 0.001, Wilcoxon signed-ranks test. Scale bar in (A) is 50 µm.

DISCUSSION

During the last decade, the study of microglial cells and neuroinflammation has experienced a revolution. Minimally invasive methods have revealed microglia to be highly dynamic in their interaction with the microenvironment, responding to inflammatory signals (Nimmerjahn et al., 2005; Davalos et al., 2005) and interacting with neuronal circuits at the synaptic level (Tremblay et al., 2010; Wake et al., 2009). Microglia can sculpt the brain and affect its physiology, because they have been observed to contain phagocytic inclusions with features of axonal terminals, dendritic spines, or unneeded neuronal progenitors (Tremblay et al., 2010; Paolicelli et al., 2011; Sierra et al., 2010). Our data demonstrate that microglial cells are actively renewed and that the brain population is maintained by a finely tuned balance of proliferation and apoptosis.

It has long been assumed that microglia are long-lived cells. At the population level, microglia are long lived, but at the individual cell level, they are not. The microglial landscape changes radically within a few weeks, with cells dying, other taking their place, and their absolute position changing. This renewing landscape will likely influence the interpretation of phenomena such as microglial priming, in which the microglial response is exaggerated (stronger than that observed in stimulus-naive microglia) to a secondary insult. This is perhaps best illustrated when the first (priming) and second stimuli are separated by prolonged periods of time in the context of adult responses to early-life infections

Figure 6. Transcriptomic Profiling of Microglia from WT and Vav-Bcl2 Mice

(A) Flow cytometry analysis and sorting of microglia from WT and Vav-Bcl2 mice. Crosshair in flow cytometry analysis and sorting plots shows gating parameters used to define $CD11b^+CD45^{low}$ and $CD11b^+CD45^{high}$ subpopulations and subsequent sorting. Statistical differences: ****p < 0.0001. Data were analyzed with a t test (A).

(B) Heatmap representation of genes showing a significant (p < 0.01; >10-fold change) change in Vav-Bcl2 versus WT microglia (combined $CD45^+$). Clustering of genes by expression profile is shown on the left.

(C) Clustered representation (GO Slim) of GO processes significantly altered in Vav-Bcl2 compared to WT microglia. Number of genes altered per cluster is shown on top of the bars.

(D) Enrichment map of GO terms, where red nodes represent GO terms and green edges represent shared genes (thicker lines indicate more shared genes).

(E) Venn diagram representing the intersection of the transcriptional variability observed when comparing total (blue), $CD45^{low}$ (green), or $CD45^{high}$ (yellow) Vav-Bcl2 to WT microglia.

(Bilbo and Schwarz, 2009), delayed inflammation after traumatic brain injury (TBI) (Johnson et al., 2013), or the onset of age-related amyloid deposition after gestational inflammation (Krstic et al., 2012). There, microglial priming implies the need for a "microglial memory" of the first stimulus to elicit an exaggerated response to the second stimulus. We suggest that our findings could either support the hypothesis that microglial priming is achieved through epigenetic (inheritable) changes (Schwarz et al., 2011) or suggest that microglia memory could be stored elsewhere in the neural or glial network. We believe that the parameters defined here will stimulate a reinterpretation of many of the functions of microglia in health and disease.

That adult microglial population is maintained at least partly by self-renewal has been largely assumed for more than 2 decades, since our group reported on the proliferation of resident microglia (Lawson et al., 1992). Using [3H]-thymidine incorporation and detection with autoradiography, Lawson et al. (1992) described a very low turnover rate for microglia, with ~0.05% of the cells dividing at a time. Although a probable underestimation, acknowledged at the time due to the relative insensitivity of the method, these studies were never revisited using the gold standard in the field: incorporation of BrdU. We now show, using three independent methods, that the proliferation index of microglia is much higher than expected (more than ten times higher), and an average of 0.69% microglial cells are in S phase at a given time. This rate would allow for an estimation of the brain's microglial population being renewed every ~95 days, allowing several cycles of renewal within the lifetime of a mouse. Higher rates are found in the human brain (average 2%), with a more dramatic consequence on the turnover cycles, leading to estimates that microglia would cycle hundreds of times during 80 years of life. However, the estimation of the turnover rate of human microglia would need alternative methods to provide a more accurate calculation and allow extrapolation to the average human population.

This homeostatic microglial proliferation is balanced by the opposing force of microglial apoptosis. The apoptotic cascade controlling microglial death seems to be dependent on the pro-apoptotic molecule BIM but not PUMA, as shown from the analysis of knockout (KO) mice. A report demonstrated that deficiency in PUMA leads to decreased numbers of both retinal and brain microglia because of unexpected roles of this protein in promoting cell survival (Zhang et al., 2012), consistent with our present findings showing a decreased density of microglia in PUMA$^{-/-}$ mice. Microglial death can be counteracted by the inhibition of mitochondrial apoptosis as indicated by the overexpression of Bcl2, leading to increased microglial numbers. Surprisingly, our results support the hypothesis that the brain can only accommodate certain number of microglial cells, because deficient microglial death causes the increased microglial numbers to plateau after postnatal development. In the normal brain, the microglial population displays a mosaic-like organization in which processes of individual cells avoid contact with one another, being disrupted only with the emergence of changes related to pathology, age, or systemic influences (Gomez-Nicola and Perry, 2015). Transcriptomic profiling of microglia from Vav-Bcl2 mice highlights profound alterations of their functional profile, including altered metabolism and immune response, providing a link between homeostatic microglial apoptosis and phenotypic profile. A more detailed future study of the mechanisms by which altered microglia turnover could affect basic microglia functions, including those controlling their inflammatory properties, will provide valuable insights into understanding the maintenance of the microglial population in health and disease.

The sub-regional analysis of microglial turnover highlighted the DG as a particularly active anatomical region. In the DG, microglial proliferation is higher, is quicker, and decays more rapidly with age. An age-dependent decrease in proliferation is also observed in the population of DG neural progenitors (Kuhn et al., 1996), and this can be correlated with the decrease of microglial proliferation, because microglial phagocytosis of progenitors is coupled to neurogenic activity (Sierra et al., 2010). Thus, aging would lead to a decrease in DG microglial proliferation indirectly, through a decline in neurogenesis. A more direct effect of aging on microglia residing within the DG would imply microglial senescence. Replicative senescence, the loss of mitotic potential accompanied by significant telomere shortening, occurs once a cell has undergone approximately 50 replications, the so-called Hayflick limit (Hayflick, 1965). Thus, we hypothesize that the increased microglial turnover in the DG will lead to a quicker extinction of the proliferative capacity of these cells, as observed in our data. The microglial population at the DG seems particularly susceptible to telomere shortening, as highlighted using a mouse model of telomere dysfunction (telomerase RNA component [TERC] KO) (Khan et al., 2015), supporting the hypothesis that increased microglial division can lead to replicative senescence.

In an attempt to reconcile conflicting evidence about the contribution of circulating monocytes to the composition of the microglial population at perinatal stages (Ginhoux et al., 2013), we developed a cell-tracking approach based on the intra-liver tracing of embryonic hematopoiesis. Our results support the existence of a wave of monocytes that infiltrate the brain, peaking at P3, but indicate that these are rapidly depleted by apoptosis and do not contribute to the final microglial population, which we can now confirm is exclusively formed by yolk sac-derived progenitors. The elimination of infiltrated macrophages is coincident with a wave of microglial proliferation, followed by a further selection process before the final number of cells is achieved (Nikodemova et al., 2015). The functional significance of this wave of liver-derived monocytes is unknown, and should be the subject of future research, but the temporal coincidence with the refinement of the microglial numbers prompts speculation that these cells could trigger the death of a subpopulation of yolk sac-derived microglia.

The changes in microglial morphology that occur during aging are well documented; however, a particular morphological change that has received little attention to date is the formation of giant, multinucleated microglial aggregates in aged mice, such as those observed in our study. Previous studies have shown that microglia form aggregates with multiple nuclei, which can include more than 20 individual cells, under certain inflammatory conditions (Fendrick et al., 2007; Perry et al., 1993). Our data show that these aggregates are not generated by failed cytokinesis after division. Similar structures are observed in the context of a repopulation paradigm after genetic ablation of

microglia (Bruttger et al., 2015). However, these clusters are BrdU+ (Bruttger et al., 2015), transient, and not fused, suggesting they serve as pools of repopulating microglia, whereas we here observe a fusion or aggregation of groups of cells. Given the territorial nature and lack of contact between microglial cells in younger brains, this is seen as an aberrant morphological development and represents a significant change in the phenotype and function of microglia in aging. With our data, we can provide clear evidence that these aggregates likely originate from the incorporation of circulating monocytes into the brain parenchyma, but further research is needed to fully understand their function.

One question raised by our data relates to the molecular regulation of the self-renewal process. We provide evidence for a necessary, but not sufficient, role of CSF1R in controlling microglial turnover in homeostasis, as shown from our mRNA studies and from the pharmacological inhibition of the CSF1R tyrosine kinase activity with GW2580. Elimination of microglia can be achieved by acute treatment with a highly potent CSF1R/c-kit/FLT3/PDGFRβ inhibitor, highlighting the relevance of these receptors in maintaining microglial numbers (Elmore et al., 2014). However, other systems may have to be in place to fully control microglial turnover. Recent studies, using genetic ablation of microglia, show that interleukin-1 receptor (IL-1R) plays a crucial role in the replenishment process (Bruttger et al., 2015). Although a repopulation process cannot be compared to the homeostatic maintenance of the microglial population, complementary systems (CSF1R, IL-1R, or others) must be in place to ensure the stability of this important population of non-neuronal cells in the CNS.

In light of the current data, we conclude that the turnover of the microglial population is a highly dynamic process, made possible by the finely tuned temporal and spatial balance of microglial proliferation and apoptosis. Our data question the view of microglia as a long-lived population, almost never renewed in the adult brain, and propose a more dynamic scenario, which will help uncover the key microglial functions in the healthy and the diseased brain.

EXPERIMENTAL PROCEDURES

Experimental Mice

All experimental procedures were either approved by a local ethical review committee and conducted in accordance with personal and project licenses under the UK Animals (Scientific Procedures) Act (1986) or performed in accordance with institutional animal welfare guidelines and were approved by the government of Baden-Wurttemberg, Germany. Details of experimental mice can be found in the Supplemental Information.

To analyze cell proliferation, mice received injections of intraperitoneal (i.p.) BrdU (Sigma-Aldrich; 7.5 mg/mL, 0.1 mL/10 g weight in sterile saline). A dose-response experiment was performed using 3.75, 7.5, or 15 mg/mL BrdU.

Postmortem Human Brain Samples

For immunohistochemical analysis, human brain autopsy tissue samples (n = 15 per group) (temporal cortex, paraffin-embedded, formalin-fixed, 96% formic acid-treated, 6 μm sections) from the National CJD Surveillance Unit Brain Bank were obtained from Alzheimer's disease or variant Creutzfeldt-Jakob disease (vCJD) non-diseased age- and sex-matched young (age 20–35) or aged (age 58–79) controls, from whom consent for use of autopsy tissues for research had been obtained. Ethical permission for research on autopsy materials stored in the National CJD Surveillance Unit was obtained from Lothian Region Ethics Committee.

In Utero Intra-liver Tracing of Embryonic Hematopoiesis

Cell tracking was performed by the administration of VSVG-SFFV-Venus or VSVG-SFFV-mCherry lentiviral vectors. Details on the design, production, and application of these vectors can be found in the Supplemental Information.

Chronic Cranial Window Implantation

The chronic cranial window was installed as previously described (Kovalchuk et al., 2015). A detailed description of the method can be found in the Supplemental Information.

Two-Photon Imaging

Microglia expressing EGFP in the olfactory bulb (OB) of $CX_3CR1^{GFP/+}$ mice were imaged once a day for 10 to 22 days by means of two-photon microscopy. Details of the method can be found in the Supplemental Information.

Immunohistochemistry

Coronal hippocampal sections were cut from paraformaldehyde-fixed, frozen, or fresh brains. Mice perfusion, tissue processing, and immunohistochemical analysis were performed as previously described (Gómez-Nicola et al., 2013), with details found in the Supplemental Information.

Statistical Analysis

Data were expressed as mean ± SEM and analyzed with the GraphPad Prism 5 software package. When normality and homoscedasticity assumptions were reached, we applied the two-tailed Fisher t test, one-way or two-way ANOVA, followed by the Tukey post hoc test for multiple comparisons. For the analysis of two-photon imaging for normally distributed data, mean ± SEM was calculated, and the Student's t test was used for comparison of two groups. For data that were not normally distributed, median ± 1 interquartile range was presented as a boxplot, and 10 to 90 percentiles were shown as whiskers. For comparisons between non-parametrically distributed groups, we used the Mann-Whitney test. The Wilcoxon matched pairs test was used for nonparametric comparison of two paired groups. All statistical tests were two sided. Differences were considered significant for $p < 0.05$.

SUPPLEMENTAL INFORMATION

Supplemental Information includes Supplemental Experimental Procedures, six figures, and one table and can be found with this article online at http://dx.doi.org/10.1016/j.celrep.2016.12.041.

AUTHOR CONTRIBUTIONS

D.G.-N., V.H.P., and O.G. conceived and designed the study. K.A., Y.L., A.O.-A., F.G.-M., K.L., P.R., T.T., M.A.C., and D.G.-N. performed the in vivo experimental work and analyzed the experimental data. K.R., Z.M., A.S., S.B., M.S.C., O.G., and V.H.P. provided reagents, equipment, and/or experimental samples. D.G.-N. supervised the study and wrote the manuscript. All authors edited and approved the manuscript.

ACKNOWLEDGMENTS

We thank the National CJD Surveillance Unit Brain Bank (Edinburgh, UK) for the provision of human samples. We thank Prof. Andreas Strasser (Melbourne, Australia) for the gift of the Vav-Bcl2 mice and Prof. Axel Schambach (MH Hannover, Germany) for the γ-retroviral vector RSF91.GFP.pre* and the packaging plasmid pcDNA3.MLVgp. The research was funded by the Medical Research Council (MR/K022687/1), by a vice-chancellor PhD studentship (K.A.), by grants from the Spanish Ministry of Economy and Competitiveness with FEDER funds (BFU2015-66689 and RYC-2013-12817) and by the Alzheimer Forschung Initiative e.V.

Received: March 6, 2016
Revised: October 28, 2016
Accepted: December 13, 2016
Published: January 10, 2017

REFERENCES

Alliot, F., Godin, I., and Pessac, B. (1999). Microglia derive from progenitors, originating from the yolk sac, and which proliferate in the brain. Brain Res. Dev. Brain Res. *117*, 145–152.

Bilbo, S.D., and Schwarz, J.M. (2009). Early-life programming of later-life brain and behavior: a critical role for the immune system. Front. Behav. Neurosci. *3*, 14.

Bruttger, J., Karram, K., Wörtge, S., Regen, T., Marini, F., Hoppmann, N., Klein, M., Blank, T., Yona, S., Wolf, Y., et al. (2015). Genetic cell ablation reveals clusters of local self-renewing microglia in the mammalian central nervous system. Immunity *43*, 92–106.

Cameron, I.L., and Greulich, R.C. (1963). Evidence for an essentially constant duration of DNA synthesis in renewing epithelia of the adult mouse. J. Cell Biol. *18*, 31–40.

Cunningham, C.L., Martínez-Cerdeño, V., and Noctor, S.C. (2013). Microglia regulate the number of neural precursor cells in the developing cerebral cortex. J. Neurosci. *33*, 4216–4233.

Davalos, D., Grutzendler, J., Yang, G., Kim, J.V., Zuo, Y., Jung, S., Littman, D.R., Dustin, M.L., and Gan, W.B. (2005). ATP mediates rapid microglial response to local brain injury in vivo. Nat. Neurosci. *8*, 752–758.

De Lucia, C., Rinchon, A., Olmos-Alonso, A., Riecken, K., Fehse, B., Boche, D., Perry, V.H., and Gomez-Nicola, D. (2016). Microglia regulate hippocampal neurogenesis during chronic neurodegeneration. Brain Behav. Immun. *55*, 179–190.

Egle, A., Harris, A.W., Bath, M.L., O'Reilly, L., and Cory, S. (2004). VavP-Bcl2 transgenic mice develop follicular lymphoma preceded by germinal center hyperplasia. Blood *103*, 2276–2283.

Elmore, M.R., Najafi, A.R., Koike, M.A., Dagher, N.N., Spangenberg, E.E., Rice, R.A., Kitazawa, M., Matusow, B., Nguyen, H., West, B.L., and Green, K.N. (2014). Colony-stimulating factor 1 receptor signaling is necessary for microglia viability, unmasking a microglia progenitor cell in the adult brain. Neuron *82*, 380–397.

Fendrick, S.E., Xue, Q.S., and Streit, W.J. (2007). Formation of multinucleated giant cells and microglial degeneration in rats expressing a mutant Cu/Zn superoxide dismutase gene. J. Neuroinflammation *4*, 9.

Ginhoux, F., Greter, M., Leboeuf, M., Nandi, S., See, P., Gokhan, S., Mehler, M.F., Conway, S.J., Ng, L.G., Stanley, E.R., et al. (2010). Fate mapping analysis reveals that adult microglia derive from primitive macrophages. Science *330*, 841–845.

Ginhoux, F., Lim, S., Hoeffel, G., Low, D., and Huber, T. (2013). Origin and differentiation of microglia. Front. Cell. Neurosci. *7*, 45.

Gomez-Nicola, D., and Perry, V.H. (2015). Microglial dynamics and role in the healthy and diseased brain: a paradigm of functional plasticity. Neuroscientist *21*, 169–184.

Gómez-Nicola, D., Fransen, N.L., Suzzi, S., and Perry, V.H. (2013). Regulation of microglial proliferation during chronic neurodegeneration. J. Neurosci. *33*, 2481–2493.

Gomez-Nicola, D., Riecken, K., Fehse, B., and Perry, V.H. (2014). In-vivo RGB marking and multicolour single-cell tracking in the adult brain. Sci. Rep. *4*, 7520.

Grabert, K., Michoel, T., Karavolos, M.H., Clohisey, S., Baillie, J.K., Stevens, M.P., Freeman, T.C., Summers, K.M., and McColl, B.W. (2016). Microglial brain region-dependent diversity and selective regional sensitivities to aging. Nat. Neurosci. *19*, 504–516.

Hashimoto, D., Chow, A., Noizat, C., Teo, P., Beasley, M.B., Leboeuf, M., Becker, C.D., See, P., Price, J., Lucas, D., et al. (2013). Tissue-resident macrophages self-maintain locally throughout adult life with minimal contribution from circulating monocytes. Immunity *38*, 792–804.

Hayflick, L. (1965). The limited in vitro lifetime of human diploid cell strains. Exp. Cell Res. *37*, 614–636.

Hoeffel, G., Chen, J., Lavin, Y., Low, D., Almeida, F.F., See, P., Beaudin, A.E., Lum, J., Low, I., Forsberg, E.C., et al. (2015). C-Myb(+) erythro-myeloid progenitor-derived fetal monocytes give rise to adult tissue-resident macrophages. Immunity *42*, 665–678.

Johnson, V.E., Stewart, J.E., Begbie, F.D., Trojanowski, J.Q., Smith, D.H., and Stewart, W. (2013). Inflammation and white matter degeneration persist for years after a single traumatic brain injury. Brain *136*, 28–42.

Kee, N., Sivalingam, S., Boonstra, R., and Wojtowicz, J.M. (2002). The utility of Ki-67 and BrdU as proliferative markers of adult neurogenesis. J. Neurosci. Methods *115*, 97–105.

Khan, A.M., Babcock, A.A., Saeed, H., Myhre, C.L., Kassem, M., and Finsen, B. (2015). Telomere dysfunction reduces microglial numbers without fully inducing an aging phenotype. Neurobiol. Aging *36*, 2164–2175.

Kovalchuk, Y., Homma, R., Liang, Y., Maslyukov, A., Hermes, M., Thestrup, T., Griesbeck, O., Ninkovic, J., Cohen, L.B., and Garaschuk, O. (2015). In vivo odourant response properties of migrating adult-born neurons in the mouse olfactory bulb. Nat. Commun. *6*, 6349.

Krstic, D., Madhusudan, A., Doehner, J., Vogel, P., Notter, T., Imhof, C., Manalastas, A., Hilfiker, M., Pfister, S., Schwerdel, C., et al. (2012). Systemic immune challenges trigger and drive Alzheimer-like neuropathology in mice. J. Neuroinflammation *9*, 151.

Kueh, H.Y., Champhekar, A., Nutt, S.L., Elowitz, M.B., and Rothenberg, E.V. (2013). Positive feedback between PU.1 and the cell cycle controls myeloid differentiation. Science *341*, 670–673.

Kuhn, H.G., Dickinson-Anson, H., and Gage, F.H. (1996). Neurogenesis in the dentate gyrus of the adult rat: age-related decrease of neuronal progenitor proliferation. J. Neurosci. *16*, 2027–2033.

Lawson, L.J., Perry, V.H., Dri, P., and Gordon, S. (1990). Heterogeneity in the distribution and morphology of microglia in the normal adult mouse brain. Neuroscience *39*, 151–170.

Lawson, L.J., Perry, V.H., and Gordon, S. (1992). Turnover of resident microglia in the normal adult mouse brain. Neuroscience *48*, 405–415.

Mignone, J.L., Kukekov, V., Chiang, A.S., Steindler, D., and Enikolopov, G. (2004). Neural stem and progenitor cells in nestin-GFP transgenic mice. J. Comp. Neurol. *469*, 311–324.

Mittelbronn, M., Dietz, K., Schluesener, H.J., and Meyermann, R. (2001). Local distribution of microglia in the normal adult human central nervous system differs by up to one order of magnitude. Acta Neuropathol. *101*, 249–255.

Nikodemova, M., Kimyon, R.S., De, I., Small, A.L., Collier, L.S., and Watters, J.J. (2015). Microglial numbers attain adult levels after undergoing a rapid decrease in cell number in the third postnatal week. J. Neuroimmunol. *278*, 280–288.

Nimmerjahn, A., Kirchhoff, F., and Helmchen, F. (2005). Resting microglial cells are highly dynamic surveillants of brain parenchyma in vivo. Science *308*, 1314–1318.

Olmos-Alonso, A., Schetters, S.T., Sri, S., Askew, K., Mancuso, R., Vargas-Caballero, M., Holscher, C., Perry, V.H., and Gomez-Nicola, D. (2016). Pharmacological targeting of CSF1R inhibits microglial proliferation and prevents the progression of Alzheimer's-like pathology. Brain *139*, 891–907.

Paolicelli, R.C., Bolasco, G., Pagani, F., Maggi, L., Scianni, M., Panzanelli, P., Giustetto, M., Ferreira, T.A., Guiducci, E., Dumas, L., et al. (2011). Synaptic pruning by microglia is necessary for normal brain development. Science *333*, 1456–1458.

Perry, V.H., Matyszak, M.K., and Fearn, S. (1993). Altered antigen expression of microglia in the aged rodent CNS. Glia *7*, 60–67.

Schwarz, J.M., Hutchinson, M.R., and Bilbo, S.D. (2011). Early-life experience decreases drug-induced reinstatement of morphine CPP in adulthood via microglial-specific epigenetic programming of anti-inflammatory IL-10 expression. J. Neurosci. *31*, 17835–17847.

Serbina, N.V., and Pamer, E.G. (2006). Monocyte emigration from bone marrow during bacterial infection requires signals mediated by chemokine receptor CCR2. Nat. Immunol. *7*, 311–317.

Shechter, R., Miller, O., Yovel, G., Rosenzweig, N., London, A., Ruckh, J., Kim, K.W., Klein, E., Kalchenko, V., Bendel, P., et al. (2013). Recruitment of beneficial M2 macrophages to injured spinal cord is orchestrated by remote brain choroid plexus. Immunity *38*, 555–569.

Sheng, J., Ruedl, C., and Karjalainen, K. (2015). Most tissue-resident macrophages except microglia are derived from fetal hematopoietic stem cells. Immunity *43*, 382–393.

Sierra, A., Encinas, J.M., Deudero, J.J., Chancey, J.H., Enikolopov, G., Overstreet-Wadiche, L.S., Tsirka, S.E., and Maletic-Savatic, M. (2010). Microglia shape adult hippocampal neurogenesis through apoptosis-coupled phagocytosis. Cell Stem Cell *7*, 483–495.

Sierra, A., Abiega, O., Shahraz, A., and Neumann, H. (2013). Janus-faced microglia: beneficial and detrimental consequences of microglial phagocytosis. Front. Cell. Neurosci. *7*, 6.

Squarzoni, P., Oller, G., Hoeffel, G., Pont-Lezica, L., Rostaing, P., Low, D., Bessis, A., Ginhoux, F., and Garel, S. (2014). Microglia modulate wiring of the embryonic forebrain. Cell Rep. *8*, 1271–1279.

Tambuyzer, B.R., Ponsaerts, P., and Nouwen, E.J. (2009). Microglia: gatekeepers of central nervous system immunology. J. Leukoc. Biol. *85*, 352–370.

Tremblay, M.E., Lowery, R.L., and Majewska, A.K. (2010). Microglial interactions with synapses are modulated by visual experience. PLoS Biol. *8*, e1000527.

Uitdehaag, J.C., Sünnen, C.M., van Doornmalen, A.M., de Rouw, N., Oubrie, A., Azevedo, R., Ziebell, M., Nickbarg, E., Karstens, W.J., and Ruygrok, S. (2011). Multidimensional profiling of CSF1R screening hits and inhibitors: assessing cellular activity, target residence time, and selectivity in a higher throughput way. J. Biomol. Screen. *16*, 1007–1017.

Varvel, N.H., Grathwohl, S.A., Baumann, F., Liebig, C., Bosch, A., Brawek, B., Thal, D.R., Charo, I.F., Heppner, F.L., Aguzzi, A., et al. (2012). Microglial repopulation model reveals a robust homeostatic process for replacing CNS myeloid cells. Proc. Natl. Acad. Sci. USA *109*, 18150–18155.

Wake, H., Moorhouse, A.J., Jinno, S., Kohsaka, S., and Nabekura, J. (2009). Resting microglia directly monitor the functional state of synapses in vivo and determine the fate of ischemic terminals. J. Neurosci. *29*, 3974–3980.

Zhang, F., Li, Y., Tang, Z., Kumar, A., Lee, C., Zhang, L., Zhu, C., Klotzsche-von Ameln, A., Wang, B., Gao, Z., et al. (2012). Proliferative and survival effects of PUMA promote angiogenesis. Cell Rep. *2*, 1272–1285.

Cell Reports, Volume *18*

Supplemental Information

Coupled Proliferation and Apoptosis Maintain the Rapid Turnover of Microglia in the Adult Brain

Katharine Askew, Kaizhen Li, Adrian Olmos-Alonso, Fernando Garcia-Moreno, Yajie Liang, Philippa Richardson, Tom Tipton, Mark A. Chapman, Kristoffer Riecken, Sol Beccari, Amanda Sierra, Zoltán Molnár, Mark S. Cragg, Olga Garaschuk, V. Hugh Perry, and Diego Gomez-Nicola

Supplemental figures

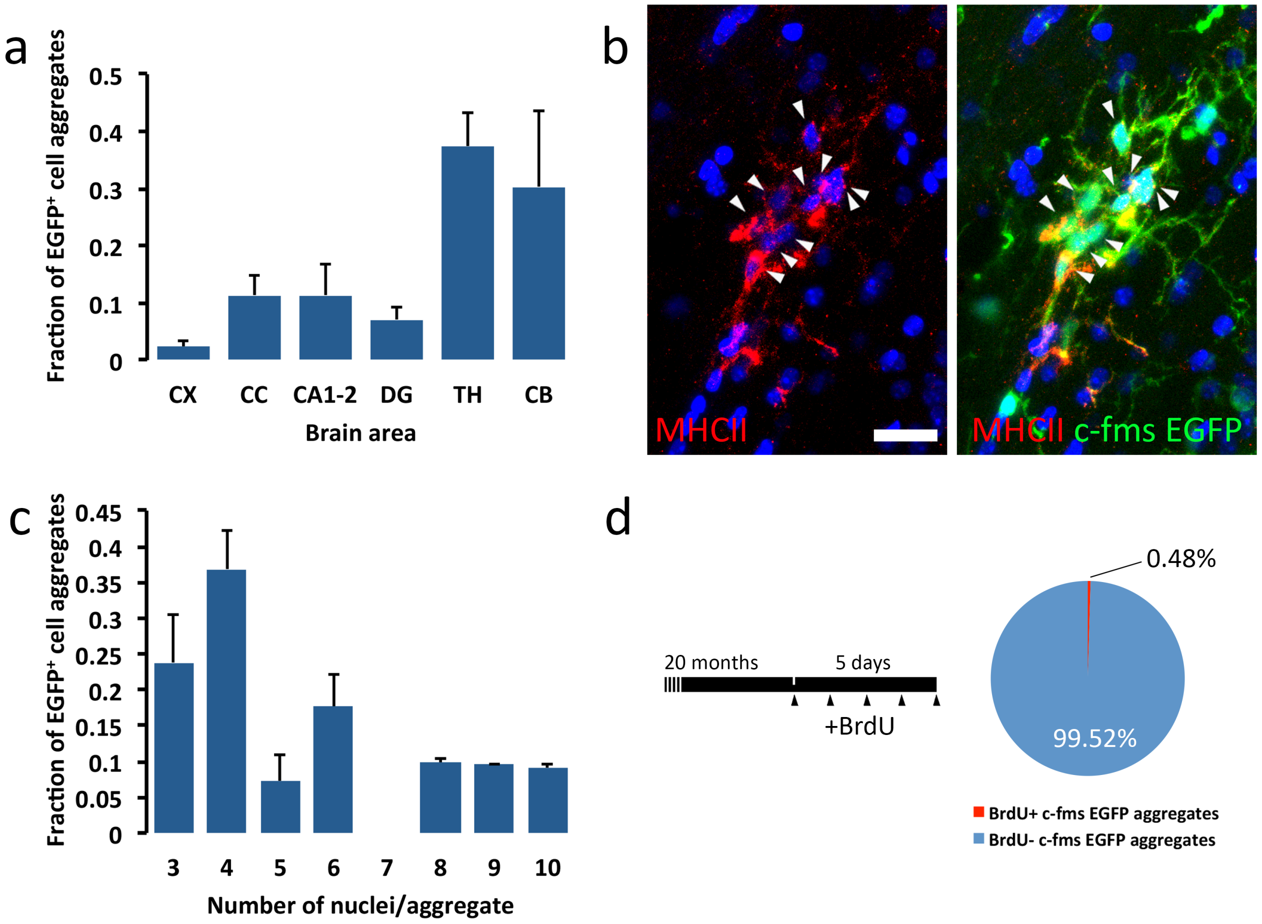

Figure S1 (related to Figure 2). Characterization of multinucleated microglial aggregates in the mouse aged brain.

(a) Distribution of microglial aggregates (c-fms EGFP$^+$) across brain regions (CX, cortex; CC, corpus callosum; CA1-2, hippocampal CA1-2; DG, dentate gyrus; TH, thalamus; CB, cerebellum) in aged (18-24 months) mice. **(b)** Expression of MHCII (red) in microglial aggregates (c-fms EGFP$^+$). Arrowheads indicate microglial nuclei. **(c)** Composition of the microglial aggregates (number of nuclei per aggregate). **(d)** Analysis of cell proliferation in microglial aggregates, after repeated dosing of BrdU (see experimental design). Scale bar in (**b**) 20mm.

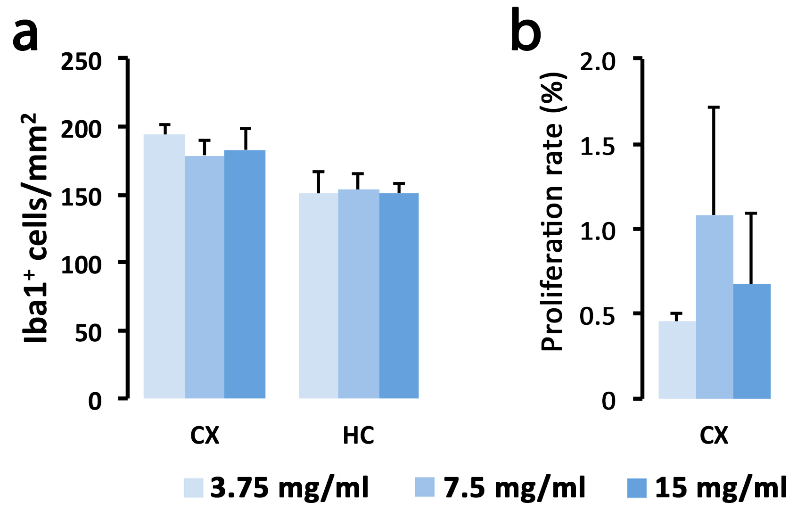

Figure S2 (related to Figure 3). Dose-response analysis of the incorporation of BrdU in microglial cells.

(a) Microglial density in the cortex (CX) or hippocampus (HC) after dosage with increasing doses of BrdU (3.75, 7.5 or 15 mg/kg). **(b)** Microglial proliferation ($Iba1^{+}BrdU^{+}$; proliferation rate, %) in the cortex (CX) or hippocampus (HC) after dosage with increasing doses of BrdU (3.75, 7.5 or 15 mg/Kg). N=4.

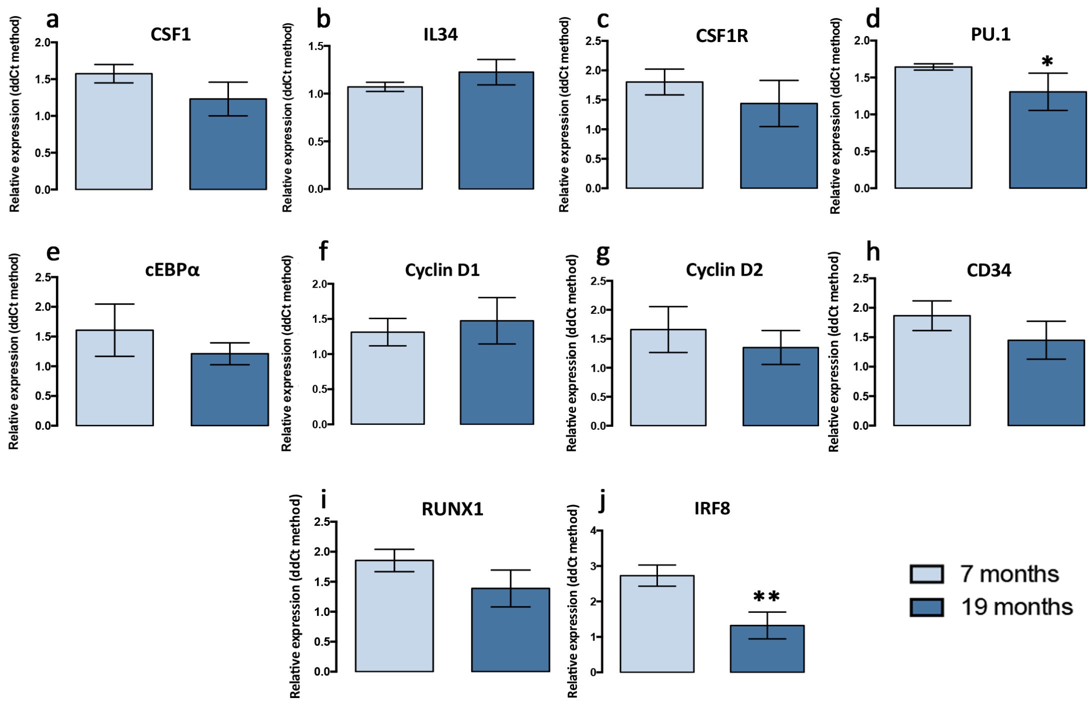

Figure S3 (related to Figure 3). Gene expression of the components of the CSF1R pathway and proliferation markers in the aged mouse brain.

(**a-j**) RT-PCR analysis of the mRNA expression of CSF1 **(a)**, IL34 **(b)**, CSF1R **(c)**, PU.1 **(d)**, C/EBPα **(e)**, Cyclin D1 **(f)**, Cyclin D2 **(g)**, CD34 **(h)**, RUNX1 **(i)** and IRF8 **(j)** in the brains of young (7 months) or aged (19 months) WT mice. Expression of mRNA represented as mean±SEM and indicated as relative expression compared to the housekeeping gene (GAPDH) using the 2-ΔΔCT method. Statistical differences: *$p<0.05$, **$p<0.01$. Data were analysed with a T-test. N=6.

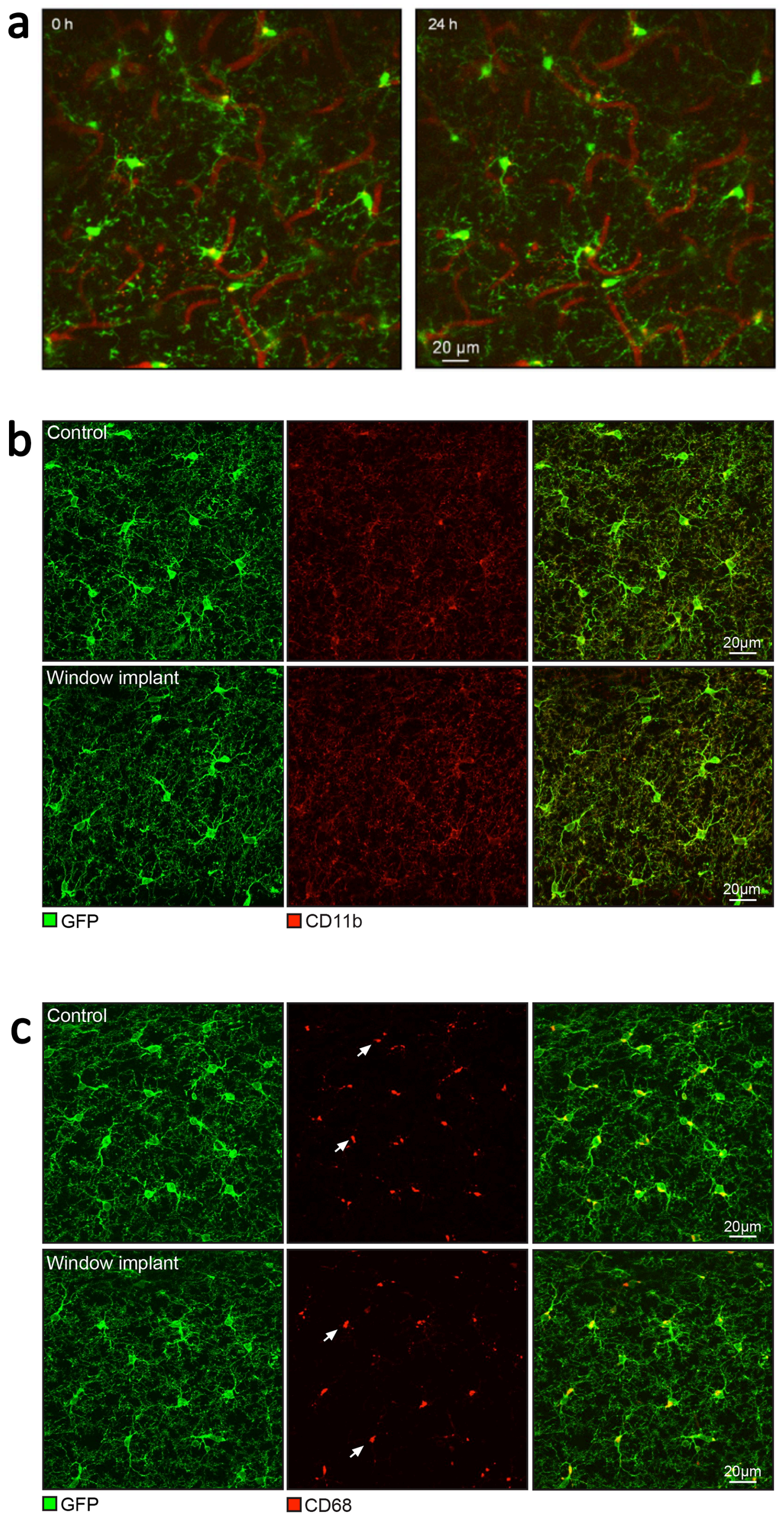

Figure S4 (related to Figure 3). Chronic in vivo imaging of olfactory bulb microglia.

(**a**) Maximal intensity projection (MIP) images of the same field of view (95 - 115 µm depth, step 1 µm) in the olfactory bulb of a $CX_3CR1^{GFP/+}$ mouse taken 24 hours apart. For repeated blood vessel imaging, sulforhodamine B (0.2 ml, 1 mM in PBS) was injected intraperitoneally at the beginning of each imaging session (as described in Kovalchuk et al., 2015). Scale bar 20mm. (**b, c**) Maximum intensity projection images (0-30 µm, 1 µm step) of the glomerular layer of the olfactory bulb in fixed slices obtained from control (upper panels) and window-implanted (lower panels)

$CX_3CR1^{GFP/+}$ transgenic mice. The GFP signal was enhanced by anti-GFP antibody and a secondary antibody AF 488 (green). Anti-CD11b (**a**) and anti-CD68 (**b**) antibodies are coupled to a secondary antibody AF 594 (red). White arrowheads point to CD68-positive lysosomes (Safaiyan et al., 2016). Images are representative for 20-30 fields of view imaged in 2 mice per group. All mice were littermates from the same litter. Scale bar is 20 µm.

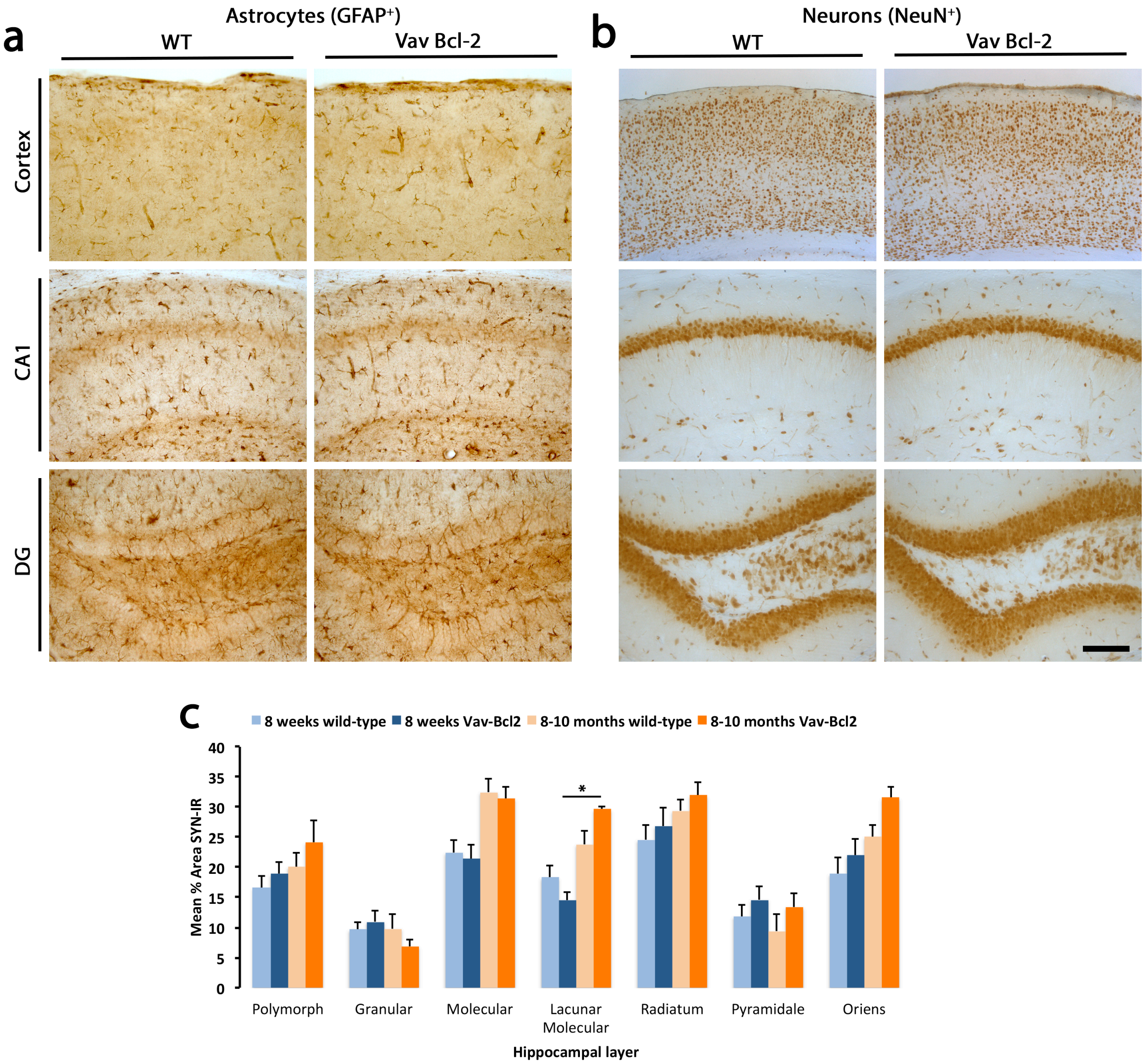

Figure S5 (related to Figure 5). Characterization of the astrocyte and neuronal populations in Vav-Bcl2 mice. **(a)** Analysis of the distribution and morphology of astrocytes ($GFAP^+$) in the cortex, hippocampal CA1-2 and dentate gyrus (DG) of wild-type and Vav-Bcl2 mice. **(b)** Analysis of the density and layering of neurons ($NeuN^+$) in the cortex, hippocampal CA1-2 and dentate gyrus (DG) of wild-type and Vav-Bcl2 mice. Scale bar in (**a, b**) 100mm (in **b**). (**c**) Immunohistochemical analysis and quantification of protein levels of synaptophysin in the hippocampus of WT and Vav-Bcl2 mice at 8 weeks and 8-10 months of age. Synaptophysin levels represented as mean±SEM of % $Synaptophysin^+$ area. Statistical differences: *$p<0.05$. Data were analysed with a two-way ANOVA and a post-hoc Tukey test. N=5.

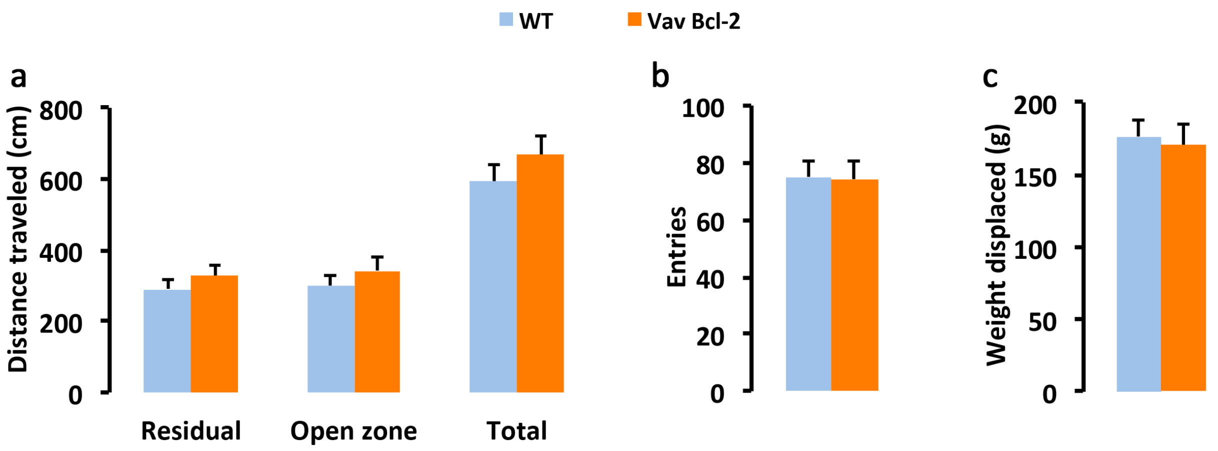

Figure S6 (related to Figure 5). Analysis of the behaviour of Vav-Bcl2 mice.

(**a, b**) Analysis of the behaviour in the open field, measured as total distanced travelled (**a**) or number of entries in the open zone (**b**) of WT and Vav-Bcl2 mice. Exploratory activity was measured as distance travelled (cm) in the open field test, analysing the locomotor activity in an open zone vs residual zone as a correlate of anxiety. (**c**) Burrowing behaviour, a measure of anhedonic behaviour, was measured as weight displaced (g) out of the tube in 24h. N=6

Table S1 (related to Figure 6). List of 396 genes differentially expressed between Vav Bcl-2 and WT.

logFC = log fold change (where positive values indicate VAV expression upregulated relative to WT and negative values indicate VAV expresssion downregulated). logCPM = log counts per million. P value - raw P value, FDR = False discovery rate corrected P value.

Supplemental Experimental Procedures

Experimental mice

C57BL/6, c-fms EGFP (macgreen)(Sasmono et al., 2003), $CCR2^{-/-}$(Menzies et al., 2012), $PUMA^{-/-}$ (Villunger et al., 2003), $BIM^{-/-}$ (Bouillet et al., 1999) and Vav Bcl2 mice (Egle et al., 2004) were bred and maintained at the local facilities of the University of Southampton (UK), while nestin-EGFP (Encinas et al., 2006) were maintained at the University of the Basque Country EHU/UPV (Leioa, Spain). Young (4-6 months) and aged (18-24months) mice were used in this study. For *in utero* intra-liver tracing experiments adult C57BL/6 mice were obtained from a local breeding colony at the University of Oxford (based on the Harlan (UK) strain). The day when vaginal plug was detected was referred to as E0. For live imaging experiments, we used 8 to 12 month-old $CX_3CR1^{GFP/+}$ mice (Jung et al., 2000), bred and maintained at the University of Tübingen (Germany).

All mice were maintained on a 12/12-hour light/dark cycle (7 AM, lights on) and provided with *ad libitum* access to food and water.

For the evaluation of the effects of treatment with GW2580 mice were 6 months of age when treatment began (N=6). Mice were fed with a control diet (RM1) or a diet containing GW2580 (Modified LabDiet® PicoLab EURodent Diet 14%, 5L0W (5LF2) with 0.1% (1000 ppm) GW2580 (LC Laboratories); TestDiet) for 3 months.

Sample size for each experiment was determined after performing power calculations, in order to achieve a significant difference of $p<0.05$, in light of a retrospective analysis of our previous results, to reach a power between 0.80-0.90, depending on the specific experimental conditions. The calculations are the customary ones based on normal distributions and were performed following statistical advice from the Research design and methodology Department of the University of Southampton.

The experiments were designed in compliance with the ARRIVE guidelines, including control groups for all experiments, randomizing the procedures and applying double-blinded analysis when possible.

***In utero* intra-liver tracing of embryonic haematopoiesis**

Design, production and application of the vectors was performed as previously described in detail(Gomez-Nicola et al., 2014) and derives from the previously described lentiviral gene-ontology vectors (LeGO (Weber et al., 2008)). Vector maps and sequence data for all vectors are available upon request; more information can be found at http://www.LentiGO-Vectors.de. Viral vectors were prepared with fast green, to allow visualization upon delivery. Exposure of living embryos was performed as previously described for *in utero* electroporation (Garcia-Moreno et al., 2014). Briefly, E14 pregnant mice were anesthetized by inhalation of isofluorane administered in conjunction with 100% oxygen. After midline laparotomy, the uterine horns were exposed out of the abdominal cavity and constantly warmed

and hydrated with pre-warmed PBS. The embryos were trans-illuminated. Embryonic livers were distinguished by their characteristic dark brown colour in the embryos' abdomen and injected with a glass electrode until fast green solution was visible. Each embryo was injected with a volume less than 1 µl comprising the mixture of viral particles (titer 10^9 particles/mL). In control experiments, the viral solution was injected in the amniotic sac, observing no subsequent cell labelling in the brain. Buprenorhpine (vetergesic; 0.05 mg/kg) and meloxicam (metacam; 0.2 mg/kg) were administered to the pregnant mice prior to and after surgery, respectively. Injected embryos were examined at different postnatal stages (P0, P3, P6, P21, P43), focusing our analysis on the cortex, hippocampus and cerebellum.

Chronic cranial window implantation

Briefly, mice were anaesthetized through intraperitoneal injection (i.p.) of ketamine/xylazine (80/4 µg/g body weight, BW, FAGRON, Barsbuettel, Germany, and Sigma, Sigma-Aldrich, St Louis, MO, respectively). Anesthetic depth was monitored by toe pinch throughout the surgery and additional ketamine/xylazine (40/2 µg/g of BW) was injected when necessary. Dexamethasone (2 µg/g of BW) was administered i.p. before the surgery and lidocaine (50 µl, AstraZeneca, Wedel, Germany) was applied subcutaneously before removing the scalp over the OB. The skull covering both OBs was cleaned and dried. A round groove (3 mm in diameter) was made with a microdrill over the two hemispheres of the OB through repeated drilling. Then the skull overlying both OBs was carefully removed, leaving the dura intact. The surface of the dura was rinsed with sterile extracellular solution (125 mM NaCl, 4.5 mM KCl, 26 mM $NaHCO_3$, 1.25 mM NaH_2PO_4, 2 mM $CaCl_2$, 1 mM $MgCl_2$ and 20 mM glucose, pH 7.4) and was then covered with a 3 mm glass coverslip (Warner Instruments, Hamden, CT). The margin between the coverslip and the skull was sealed with cyanoacrylate glue. The remaining exposed area over the OBs was covered with dental cement. Postoperative care included an analgesic dose of carprofen (5 µg/g of BW) for 3 days subcutaneously and the antibiotic baytril (Bayer, Leverkusen, Germany; 1:100 v/v) in drinking water for 10 days. After the surgery, animals were allowed to recover for at least 3 weeks and were subsequently examined for window clarity. For mice with good quality windows, a metal bar for head fixation was glued to the caudal part of the skull with dental acrylic and cement.

To assess the impact of chronic window implantation on the microglial phenotype, we performed staining on free-floating cryoslices (thickness 50 µm) at room temperature. The sections were treated with a blocking solution (5% normal donkey serum and 1% Triton-X 100 in PBS) for 2h to prevent non-specific background staining. After blocking the slices were exposed overnight to primary antibodies diluted in the blocking solution as indicated below. The following primary antibodies were used: anti-GFP (1:1000; Rockland), anti-CD11b (1:1500; AbD SeroTec), anti-CD68 (1:1000; AbD SeroTec). Thereafter the sections were rinsed in PBS three times for 10 min and incubated with AF 488- and AF 594-conjugated secondary antibodies (1:1000 in PBS +2% bovine serum albumin; Invitrogen) for 2h in

darkness. Afterwards, the sections were washed three times in PBS, transferred to Superfrost Plus charged glass slides (Langenbrink) and mounted in Vectashield Mounting Medium (Vector Laboratories).

Tracing of proliferation with γ-retroviral vectors

The delivery of Eco-SFFV mCherry γ-retroviral vectors was used to trace microglial proliferation in macgreen mice. The viral vector is a derivative of RSF91.GFP.pre* (Schambach et al., 2006), design and production was performed as previously described(Gomez-Nicola et al., 2014). Mice were anaesthetised with a ketamine/rompun mixture (85 and 13 mg/kg), and 5μl (10^9 particles/ml) of the viral particles were injected stereotaxically in the lateral ventricle at the following coordinates from bregma: anteroposterior, −0.1 mm; lateral, ±0.9 mm; depth, -2.2 mm.

Two-photon Imaging

Mice with cranial windows were anesthetized with isofluorane (2.5% in oxygen) and placed on a warming pad. The head of the mouse was fixed to X–Y microscope stage. During the experiment, concentration of isoflurane was between 0.8 and 1.5% enabling a breathing rate at ~100 breaths/min. The body temperature of the animal was kept between 36 and 37°C. Two-photon imaging was performed with a two-photon laser-scanning microscope (Olympus Fluoview 1000, Olympus, Tokyo, Japan) coupled to a Mai Tai Deep See Laser (Spectra Physics, Mountain View, CA). The emitted light was collected with a Zeiss ×20 water-immersion objective (NA 1.00). For precise cell identification across imaging sessions, angiography was performed through i.p. injection of a fluorescent dye sulforhodamine B (1mM in PBS, 0.1ml per 10g of BW, Sigma-Aldrich). 900nm excitation light (with laser power of 10-40 mW on the top of the specimen) was used for simultaneous excitation of eGFP and sulforhodamine B. The emission signals were split by a 570 nm dichroic mirror.

Behavioural tests

Vav Bcl2 or WT mice were tested on behavioural tasks at 4 months of age: open-field locomotor and exploratory activity and burrowing activity (anhedonic behaviour). The open-field tests were carried out using activity monitor software (Med Associated Inc.). These tests were performed as previous described (Gomez-Nicola et al., 2013).

Immunohistochemistry

We used the following primary antibodies: rabbit anti-Iba1 (Wako), mouse anti-human Ki67 (Dako), mouse anti-BrdU (DSHB), goat anti-Vav (Santa Cruz Biotechnologies), chicken anti-GFP/Venus (Aves Labs), rabbit anti-cleaved caspase 3 (Millipore), rat anti-MHCII (EBioscience), rabbit anti-GFAP (Dako), rabbit anti-Olig2 (Santa Cruz Biotechnologies), rabbit anti-NG2 (Millipore), rat anti-CD206 (AbD Serotec), mouse anti-NeuN (Millipore), mouse

anti-synaptophysin (SY38; Merck Millipore) and rabbit anti-PU.1 (Cell Signalling). Following primary antibody incubation, the sections were washed and incubated with the appropriate biotinylated secondary antibody (Vector Labs), and/or with the appropriate Alexa 405, 488 or 568 conjugated secondary antibody or streptavidin (Molecular Probes). For co-labelling of Iba1/Ki67 or Iba1/BrdU, following primary antibody sections were incubated with an anti-rabbit biotinylated secondary antibody (for Iba1 detection) and the ImmPRESS-AP Anti-Mouse (alkaline phosphatase) Polymer Detection Kit (for Ki67 or BrdU detection). For light microscopy, the sections were visualized using diaminobenzidine (DAB) precipitation or BCIP/NBT AP reaction, in a Leica CTR 5000 microscope, coupled to a Leica DFC300FX microscope camera. For PU.1 visualization, the DAB signal was enhanced with 0.05% Nickel Ammonium Sulfate, producing a black precipitate. After immunofluorescence labelling, nuclei were visualized by DAPI staining and the sections were mounted with Mowiol/DABCO (Sigma-Aldrich) mixture. The sections were visualized on a Leica TCS-SP5 confocal system, coupled to a Leica CTR6500 microscope.

The general immunohistochemistry protocol was modified for the detection of BrdU, adding a DNA denaturation step with 2N HCl (30min, 37°C), as previously described(Gomez-Nicola et al., 2013).

The protocol used for immunohistochemistry on human sections was a modification of the general protocol (DAB+AP), with antigen unveiling in citrate buffer being performed for 25min, as previously described(Olmos-Alonso et al., 2016).

Analysis of gene expression by RT-PCR

Young or aged mice WT mice (n=8/group) were processed to obtain samples from the hippocampus by dissection under a microscope, after intracardiac perfusion with heparinized 0.9% saline. RNA was extracted using Trizol (Life Technologies), quantified using Nanodrop (Thermo Scientific), to be retrotranscribed using the iScript cDNA Synthesis Kit (Bio-Rad), following manufacturer's instructions, after checking its integrity by electrophoresis in a 2% agarose gel. cDNA libraries were analysed by qPCR using the iTaq Universal SYBR Green supermix (Bio-Rad) and the following custom designed gene-specific primers (Sigma-Aldrich): csf1 (NM_007778.4; FW, agtattgccaaggaggtgtcag, RV, atctggcatgaagtctccattt), il34 (NM_001135100.1; FW, ctttgggaaacgagaatttggaga, RV, gcaatcctgtagttgatggggaag), csf1r (NM_001037859.2; FW, gcagtaccaccatccacttgta, RV, gtgagacactgtccttcagtgc), pu.1 (NM_011355.1; FW, cagaagggcaaccgcaagaa, RV, gccgctgaactggtaggtga), c/ebpa (NM_007678.3; FW, agcttacaacaggccaggtttc, RV, cggctggcgacatacagtac), runx1 (NM_001111021; FW, caggcaggacgaatcacact, RV, ctcgtgctggcatctctcat), irf8 (NM_008320; FW, cggggctgatctgggaaaat, RV, cacagcgtaacctcgtcttc), cd34 (NM_133654.3; FW, gccctacaggagaaaggctgggt, RV, gcccctcgggtcacattggc), cyclin D1 (NM_007631.2; FW, ggctcctctcatggcgctgc, RV, gtggcatgcacaacaggccg) and cyclin D2 (NM_009829; FW, tcgatgggctgcgttgcgtt, RV, gggagcctgcgtcaaagggg). Quality of the primers and the PCR reaction were evaluated by electrophoresis in a 1.5% agarose gel, checking the PCR product

size. Data were analysed using the 2-ΔΔCt method with Primer Opticon 3 software, using gapdh (NM_008084.2; FW, tgaacgggaagctcactgg, RV, tccaccaccctgttgctgta) as a housekeeping gene.

Microglial Fluorescent activated cell sorting (FACS) and RNAseq

Mice were terminally anaesthetised with an overdose of sodium pentobarbital and transcardially perfused with 0.9% saline solution. Brains were harvested and dissociated mechanically followed by enzymatic digestion using the MACS Neural Tissue Dissociation Kit (Miltenyi Biotech) then passed through a cell strainer of 70µm mesh (BD2 Falcon) with FACS buffer (PBS with 1% Fetal Bovine Serum (FBS) and 1mM EDTA (Sigma Aldrich)). The cell suspension was separated by 37% Percoll gradient centrifugation at 500 g for 30 min at 18°C (no brake).

Samples were immunostained with primary antibodies directed against CD11b (clone: M1/70) and CD45 (clone: 30-F11) (BD Biosciences) at 1:500 dilution at 4°C for 30 minutes. Cells were washed then analysed and sorted using a FACS Aria II (BD Biosciences). Data were acquired with FACSDiva software (Becton Dickinson). Post acquisition analysis was performed using FlowJo software version 10.2 (Tree Star).

cDNA synthesis from small pools of cells was performed at the Oxford Genomics Centre (Wellcome Trust Centre for Human Genetics) following the Smart-seq2 method (Picelli et al., 2013) and libraries prepared using Nextera XT (Illumina) with 0.25ng cDNA input and 12 PCR cycles. All libraries were pooled and sequenced on one lane HiSeq4000 at 75bp paired end. Data have been uploaded to the NCBI Short Read Archive under project PRJNA356215. Adapter-trimmed paired fastq reads were aligned to the mouse genome (GRCm38) using Hisat (Kim et al., 2015) with option --dta (increasing the stringency of transcript anchors) and outputted to a bam file using samtools (ver 1.1; (Li et al., 2009)). MarkDuplicates in Picard (https://broadinstitute.github.io/picard/) was used to identify and remove duplicate reads and featureCounts (Liao et al., 2014) within the Subread package (Liao et al., 2013) was used to provide gene counts. Gene counts from each of the libraries were combined and used to investigate differential expression (DE) with edgeR (Robinson et al., 2010). Comparisons were made between all WT and all VAV mice as well as between WT ($CD45^{high}$) and VAV ($CD45^{high}$), and WT ($CD45^{low}$) and VAV ($CD45^{low}$) and genes with a P value < 0.01 and a fold change of >10 were considered significantly DE. Applying an FDR considerably limited the number of genes identified as significantly DE, probably because of the low sample size. This was deemed too strict given our goal was to identify GO pathways which were affected in the VAV mice and was not to identify individual genes. Heatmaps were generated using the script *analyze_diff_expr.pl* in Trinity (Haas et al., 2013) and a matrix of TMM-normalised counts. Gene Ontology (GO) terms over-represented in the list of DE genes were identified using the MGI Gene Ontology Term Finder (http://www.informatics.jax.org/gotools/MGI_Term_Finder.html). Clustering of GO terms was created using Enrichment map v 2.1 (Merico et al., 2010) in Cytoscape 3.4.0 (Cline et al., 2007).

Image Analysis

For the analysis of two-photon imaging experiments Fluoview 3.0 Viewer (Olympus) was used to view stacks from the same FOV. Angiography through sulforhodamine B provides a stable landmark to distinguish immobile cells from cells that are new-born or gone. The death and proliferation rate in each FOV was presented as values normalized by the total number of cells and the number of days of observation in the same FOV. The centre to centre distance between two microglia cells was calculated by 3D ROI manager (Ollion et al., 2013) plugin of ImageJ software (http://rsb.info.nih.gov/ij/, National Institutes of Health). The centre-to-centre distance between the twin cells w was measured from the first day of the separation of the two cell bodies. The 3D matrix of the sample FOV showing the starting position of each microglia cell was generated by TrakEM2 (Cardona et al., 2012) ImageJ plugin.

For the analysis of Venus$^+$ cells in the postnatal brain (cortex, hippocampus and cerebellum), all cells were counted in a minimum of 5 sections per mouse, n=4 mice per groups. For the histological methods, the quantification of antigen positive cells (i.e. Iba1$^+$) in the different areas (n=4 fields/mouse, n=4-8 mice/group) was performed after DAB immunohistochemistry. The number of double positive cells (i.e. Iba1$^+$BrdU$^+$) in the specific area (n=4 fields/mouse, n=4-8 mice/group) was performed after double immunohistochemistry with DAB-AP. Data were represented as number of positive cells/mm^2. The quantification of antigen positive cells (i.e. Iba1$^+$Ki67$^+$ or Iba1$^+$) in human brains was performed in the white or grey matter of the temporal cortex after DAB/AP immunohistochemistry (n=20 fields/brain, n=9-10 brains/group). The proliferative rate of microglia was calculated as the % Iba1$^+$/BrdU$^+$ cells out of the total number of Iba1$^+$ cells. The quantification of the intensity of signal (i.e. synaptophysin) was performed after immunofluorescence, and presented as % stained area. All quantifications were performed with the help of the ImageJ image analysis software.

Supplemental references

BOUILLET, P., METCALF, D., HUANG, D. C., TARLINTON, D. M., KAY, T. W., KONTGEN, F., ADAMS, J. M. & STRASSER, A. 1999. Proapoptotic Bcl-2 relative Bim required for certain apoptotic responses, leukocyte homeostasis, and to preclude autoimmunity. *Science,* 286**,** 1735-8.

CARDONA, A., SAALFELD, S., SCHINDELIN, J., ARGANDA-CARRERAS, I., PREIBISCH, S., LONGAIR, M., TOMANCAK, P., HARTENSTEIN, V. & DOUGLAS, R. J. 2012. TrakEM2 software for neural circuit reconstruction. *PLoS One,* 7**,** e38011.

CLINE, M. S., SMOOT, M., CERAMI, E., KUCHINSKY, A., LANDYS, N., WORKMAN, C., CHRISTMAS, R., AVILA-CAMPILO, I., CREECH, M., GROSS, B., HANSPERS, K., ISSERLIN, R., KELLEY, R., KILLCOYNE, S., LOTIA, S., MAERE, S., MORRIS, J., ONO, K., PAVLOVIC, V., PICO, A. R., VAILAYA, A., WANG, P. L., ADLER, A., CONKLIN, B. R., HOOD, L., KUIPER, M., SANDER, C., SCHMULEVICH, I., SCHWIKOWSKI, B., WARNER, G. J., IDEKER, T. & BADER, G. D. 2007. Integration of biological networks and gene expression data using Cytoscape. *Nat Protoc,* 2**,** 2366-82.

EGLE, A., HARRIS, A. W., BATH, M. L., O'REILLY, L. & CORY, S. 2004. VavP-Bcl2 transgenic mice develop follicular lymphoma preceded by germinal center hyperplasia. *Blood,* 103**,** 2276-83.

ENCINAS, J. M., VAAHTOKARI, A. & ENIKOLOPOV, G. 2006. Fluoxetine targets early progenitor cells in the adult brain. *Proc Natl Acad Sci U S A,* 103**,** 8233-8.

GARCIA-MORENO, F., VASISTHA, N. A., BEGBIE, J. & MOLNAR, Z. 2014. CLoNe is a new method to target single progenitors and study their progeny in mouse and chick. *Development,* 141**,** 1589-98.
GOMEZ-NICOLA, D., FRANSEN, N. L., SUZZI, S. & PERRY, V. H. 2013. Regulation of microglial proliferation during chronic neurodegeneration. *J Neurosci,* 33**,** 2481-93.
GOMEZ-NICOLA, D., RIECKEN, K., FEHSE, B. & PERRY, V. H. 2014. In-vivo RGB marking and multicolour single-cell tracking in the adult brain. *Sci Rep,* 4**,** 7520.
HAAS, B. J., PAPANICOLAOU, A., YASSOUR, M., GRABHERR, M., BLOOD, P. D., BOWDEN, J., COUGER, M. B., ECCLES, D., LI, B., LIEBER, M., MACMANES, M. D., OTT, M., ORVIS, J., POCHET, N., STROZZI, F., WEEKS, N., WESTERMAN, R., WILLIAM, T., DEWEY, C. N., HENSCHEL, R., LEDUC, R. D., FRIEDMAN, N. & REGEV, A. 2013. De novo transcript sequence reconstruction from RNA-seq using the Trinity platform for reference generation and analysis. *Nat Protoc,* 8**,** 1494-512.
JUNG, S., ALIBERTI, J., GRAEMMEL, P., SUNSHINE, M. J., KREUTZBERG, G. W., SHER, A. & LITTMAN, D. R. 2000. Analysis of fractalkine receptor CX(3)CR1 function by targeted deletion and green fluorescent protein reporter gene insertion. *Mol Cell Biol,* 20**,** 4106-14.
KIM, D., LANGMEAD, B. & SALZBERG, S. L. 2015. HISAT: a fast spliced aligner with low memory requirements. *Nat Methods,* 12**,** 357-60.
LI, H., HANDSAKER, B., WYSOKER, A., FENNELL, T., RUAN, J., HOMER, N., MARTH, G., ABECASIS, G., DURBIN, R. & GENOME PROJECT DATA PROCESSING, S. 2009. The Sequence Alignment/Map format and SAMtools. *Bioinformatics,* 25**,** 2078-9.
LIAO, Y., SMYTH, G. K. & SHI, W. 2013. The Subread aligner: fast, accurate and scalable read mapping by seed-and-vote. *Nucleic Acids Res,* 41**,** e108.
LIAO, Y., SMYTH, G. K. & SHI, W. 2014. featureCounts: an efficient general purpose program for assigning sequence reads to genomic features. *Bioinformatics,* 30**,** 923-30.
MENZIES, F. M., KHAN, A. H., HIGGINS, C. A., NELSON, S. M. & NIBBS, R. J. 2012. The chemokine receptor CCR2 is not required for successful initiation of labor in mice. *Biol Reprod,* 86**,** 118.
MERICO, D., ISSERLIN, R., STUEKER, O., EMILI, A. & BADER, G. D. 2010. Enrichment map: a network-based method for gene-set enrichment visualization and interpretation. *PLoS One,* 5**,** e13984.
OLLION, J., COCHENNEC, J., LOLL, F., ESCUDE, C. & BOUDIER, T. 2013. TANGO: a generic tool for high-throughput 3D image analysis for studying nuclear organization. *Bioinformatics,* 29**,** 1840-1.
OLMOS-ALONSO, A., SCHETTERS, S. T., SRI, S., ASKEW, K., MANCUSO, R., VARGAS-CABALLERO, M., HOLSCHER, C., PERRY, V. H. & GOMEZ-NICOLA, D. 2016. Pharmacological targeting of CSF1R inhibits microglial proliferation and prevents the progression of Alzheimer's-like pathology. *Brain,* 139**,** 891-907.
PICELLI, S., BJORKLUND, A. K., FARIDANI, O. R., SAGASSER, S., WINBERG, G. & SANDBERG, R. 2013. Smart-seq2 for sensitive full-length transcriptome profiling in single cells. *Nat Methods,* 10**,** 1096-8.
ROBINSON, M. D., MCCARTHY, D. J. & SMYTH, G. K. 2010. edgeR: a Bioconductor package for differential expression analysis of digital gene expression data. *Bioinformatics,* 26**,** 139-40.
SAFAIYAN, S., KANNAIYAN, N., SNAIDERO, N., BRIOSCHI, S., BIBER, K., YONA, S., EDINGER, A. L., JUNG, S., ROSSNER, M. J. & SIMONS, M. 2016. Age-related myelin degradation burdens the clearance function of microglia during aging. *Nat Neurosci,* 19**,** 995-8.
SASMONO, R. T., OCEANDY, D., POLLARD, J. W., TONG, W., PAVLI, P., WAINWRIGHT, B. J., OSTROWSKI, M. C., HIMES, S. R. & HUME, D. A. 2003. A macrophage colony-stimulating factor receptor-green fluorescent protein transgene is expressed throughout the mononuclear phagocyte system of the mouse. *Blood,* 101**,** 1155-1163.
SCHAMBACH, A., MUELLER, D., GALLA, M., VERSTEGEN, M. M., WAGEMAKER, G., LOEW, R., BAUM, C. & BOHNE, J. 2006. Overcoming promoter competition in packaging cells improves production of self-inactivating retroviral vectors. *Gene Ther,* 13**,** 1524-33.
VILLUNGER, A., MICHALAK, E. M., COULTAS, L., MULLAUER, F., BOCK, G., AUSSERLECHNER, M. J., ADAMS, J. M. & STRASSER, A. 2003. p53- and drug-induced apoptotic responses mediated by BH3-only proteins puma and noxa. *Science,* 302**,** 1036-8.
WEBER, K., BARTSCH, U., STOCKING, C. & FEHSE, B. 2008. A multicolor panel of novel lentiviral "gene ontology" (LeGO) vectors for functional gene analysis. *Mol Ther,* 16**,** 698-706.
